# Spine biomechanical testing methodologies: The controversy of consensus vs scientific evidence

**DOI:** 10.1002/jsp2.1138

**Published:** 2021-01-05

**Authors:** John J. Costi, Eric H. Ledet, Grace D. O'Connell

**Affiliations:** ^1^ Biomechanics and Implants Research Group, Medical Device Research Institute, College of Science and Engineering Flinders University Adelaide Australia; ^2^ Department of Biomedical Engineering Rensselaer Polytechnic Institute Troy New York USA; ^3^ Research and Development Service Stratton VA Medical Center Albany New York USA; ^4^ Department of Mechanical Engineering University of California‐Berkeley Berkeley California USA; ^5^ Department of Orthopaedic Surgery University of California‐San Francisco San Francisco California USA

**Keywords:** biomechanics, consensus, controversy, in vitro, mechanical testing, methodology, spine

## Abstract

Biomechanical testing methodologies for the spine have developed over the past 50 years. During that time, there have been several paradigm shifts with respect to techniques. These techniques evolved by incorporating state‐of‐the‐art engineering principles, in vivo measurements, anatomical structure‐function relationships, and the scientific method. Multiple parametric studies have focused on the effects that the experimental technique has on outcomes. As a result, testing methodologies have evolved, but there are no standard testing protocols, which makes the comparison of findings between experiments difficult and conclusions about in vivo performance challenging. In 2019, the international spine research community was surveyed to determine the consensus on spine biomechanical testing and if the consensus opinion was consistent with the scientific evidence. More than 80 responses to the survey were received. The findings of this survey confirmed that while some methods have been commonly adopted, not all are consistent with the scientific evidence. This review summarizes the scientific literature, the current consensus, and the authors' recommendations on best practices based on the compendium of available evidence.

## 1 INTRODUCTION

In vitro cadaveric tissue, testing plays a crucial role in the understanding of human spinal biomechanics, and can be an effective means for predicting the in vivo response to mechanical stimuli or determining the effects of disease or clinical interventions. However, the ability for in vitro tests to have clinical relevance and predict in vivo performance is predicated on the quality of the study design and experimental techniques. Many factors can introduce artifacts into the data and outcomes. Careful attention to experimental methods is necessary to minimize the differences between the in vitro experimental conditions and the analogous in vivo environment to yield data with high predictive value.

Biomechanical testing methodologies for spinal motion segments have developed over the past 50 years. During that time, there have been several paradigm shifts in techniques, which evolved by incorporating state‐of‐the‐art engineering principles, in vivo measurements, anatomical structure‐function relationships, and the scientific method. While the goal of innovative testing methods is to enhance clinical relevance, the evolution of techniques has made the comparison of results across studies more complex. As a result, there has been a push toward harmonization of methods to allow for the comparison of findings between experiments.^1^, ^2^

Despite previous efforts to define best practices for in vitro biomechanical testing of spinal motion segments, there remains a wide variation in experimental approaches. However, multiple parametric studies have demonstrated that experimental techniques can significantly affect outcomes. Because of the lack of uniformity in experimental methods, we conducted a review of the literature to summarize the scientific evidence related to best practices in mechanical testing of spinal motion segments. We also surveyed spine researchers to gauge current opinions on the best methods used for testing motion segment biomechanics.

## 2 METHODS

**FIGURE 1:**
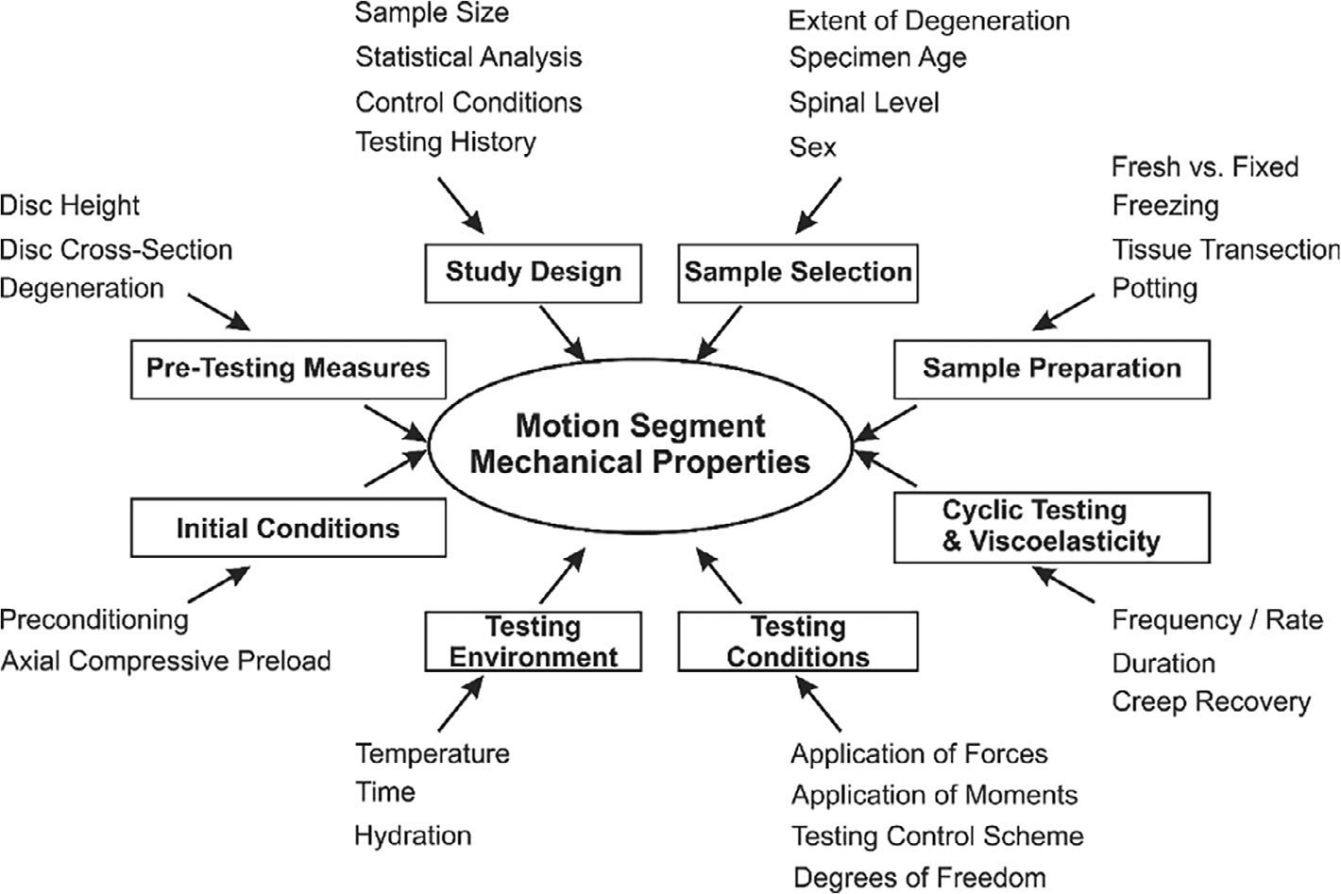
Multiple parameters related to experimental methods can affect the measured mechanical properties of spinal motion segments during in vitro testing

A literature review was conducted using the keywords “spine biomechanics.” Articles, which included in vitro testing from 1990 to 2019, were reviewed in the following categories: Sample Selection and Preparation, Pre‐Testing Measures (eg, measuring disc anatomy before testing), Initial Conditions, Testing Environment, Test Conditions, Cyclic Testing, Viscoelasticity, and Study Design (Figure 1).

Based on inconsistencies in experimental techniques found in the literature, survey questions were developed to determine if there is a consensus on techniques for biomechanical testing of spinal motion segments. While the literature review broadly encompasses motion segment biomechanics, survey questions specifically focused on methods used for human cadaver testing (Google Forms; Supporting Information S1).

The survey link was emailed to members of the Orthopaedic Research Society Spine Section, The International Society for the Study of the Lumbar Spine, and the broader international spine biomechanics research community with a request to forward the survey among colleagues.

Scientific evidence from the literature review was compared to survey responses for each category. Based on the compendium of the available evidence, the authors made recommendations on best practices for each sub‐category.

## 3 RESULTS

### 3.1 Summary of survey responses

There were 83 responses to the survey, which included 57 researchers (engineers or scientists), 23 physicians, and 3 responses from other disciplines. From this cohort, 10 researchers, 10 physicians, and 3 from other disciplines had not performed in vitro biomechanics research were excluded from the study, leaving 60 respondents with experience related to spine biomechanics. Results from the specific survey questions are reported in each relevant section below.

### 3.2 Summary of the literature

#### 3.2.1 Sample selection

Appropriate specimen selection is a critical factor for in vitro biomechanical testing of human cadaveric tissue. Factors such as the extent of degeneration, specimen age, sex, or spinal level can confound results significantly.^3^ Selection of nonhuman specimens can eliminate some of the variability inherent in human cadaveric specimens, but may also introduce additional confounding factors based on the differences in anatomy and tissue properties between human and nonhuman species.^4^

While single‐level specimens are typically used to characterize the effect of pathology or therapy on the spine, only multi‐segment constructs can be used to assess the more global effects (such as adjacent level disease) of an intervention on the kinematics and mechanics of the spine.

##### Sample selection: extent of degeneration

Intervertebral disc: Healthy discs respond to loading differently than pathologic discs. The extent of disc pathology affects inherent motion segment mechanical properties, including ROM, stiffness, and neutral zone.^5^ Mechanical properties have been measured intra‐operatively using a sensor‐instrumented vertebral distractor (spinal stiffness gauge) which flexes the spine while measuring resistance to motion. Results demonstrated a nonlinear correlation between motion segment stiffness and degeneration.^6^, ^7^ Initially, motion segment stiffness decreases in early stages of degeneration,^8^ but later increases with more advanced stages of degeneration.^9^, ^10^, ^11^, ^12^, ^13^ With increasing degeneration, the loss of proteoglycans results in a desiccated, less mobile disc, while osteophyte formation and hypertrophy of ligaments may later increase motion segment stiffness.^12^

The response of a motion segment to compressive axial load is also affected by the extent of disc degeneration.^14^, ^15^ In his pioneering work, Perey showed that the distribution of forces through the intervertebral disc to the adjacent endplate is dependent on the disc's ability to develop internal hydrostatic pressure.^16^ A healthy disc, when loaded axially, develops hydrostatic pressure in the nucleus pulposus, which results in tensile forces along with the circumferential direction of the annulus fibrosus. The net compressive force imposed on the endplates adjacent to the annulus is subsequently reduced.^14^ In degenerated discs, hydrostatic pressure is reduced due to a loss of glycosaminoglycans in the nucleus and inner annulus and applied compressive forces are transmitted directly to the endplates, which affects the response of the motion segment to axial loading.^16^, ^17^ In this way, the amount of glycosaminoglycans in the disc affects stress and strain distribution and motion segment mechanical behavior.^18^, ^19^, ^20^

Concentric and radial tears and rim lesions are common in degenerated discs,^21^, ^22^, ^23^ and there is a correlation between loss of disc height and annular fissures in degenerative discs.^7^ These changes in disc structure result in a decrease in torsional stiffness and an increase in flexion and extension stiffness when lesions are present.^7^, ^23^ Anterior rim lesions reduce peak moments in extension, lateral bending, and axial rotation.^24^ Torsional stiffness is also affected with increasing degeneration resulting in increased torsional stiffness.^25^ In the lumbar spine, there is an increase in axial rotation and lateral bending range of motion (ROM) with an increased grade of degeneration.^7^, ^26^ In the cervical spine, degenerative discs have a smaller cross‐sectional area and a more posterior instantaneous axis of rotation than their healthy counterparts,^27^ which affects both the kinematics and mechanics of the motion segment.

Degree of disc degeneration affects compressive viscoelastic properties, under dynamic and static creep loading conditions.^28^, ^29^, ^30^ The viscous modulus and viscosity are reduced with increasing degeneration. When subjected to dynamic loading, healthy discs are much more deformable and lose more height than degenerated discs, likely due to fluid egress.^31^ Radial tears have been shown to reduce the hysteresis of the disc in flexion/extension and lateral bending.^24^

In a compromised disc, such as after nucleotomy, both disc pressure and endplate strains decrease.^32^ The distribution of endplate strains is also altered in a compromised disc.^32^ Thus, the response of the disc to mechanical loading is different depending on the extent of degeneration.

*Spinal ligaments*: Similar relationships have been found between aging, degeneration, and the mechanical properties of spinal ligaments. The stress at rupture and modulus of elasticity of the ligamentum flavum decrease linearly with age^33^ and the stress at the failure of both the anterior^34^ and posterior^35^ longitudinal ligament also decreases with age. The mechanical properties (strength, modulus) of spinal ligaments also decrease with increasing disc degeneration and facet pathology.^34^, ^36^ In the initial stages of spinal degeneration, there is a decrease in ligament stiffness and strength, however, with advanced degeneration, hypertrophy of the ligaments can increase both stiffness and strength.^36^

*Facet joints*: If the facets are altered during the preparation of a specimen or if they are pathologic, the mechanical properties of the motion segment changes significantly, as facet joints contribute significantly to motion segment mechanical properties.^37^, ^38^, ^39^ Pathology of the facet joints affects not only stability and stiffness but also the motion coupling characteristics of the cervical spine.^40^ Approximately 100% of cervical spine specimens over age 50 years have facet pathology, and similar rates have been noted in the lumbar spine.^41^, ^42^

In the lumbar spine, facet joints contribute primarily to stability in axial rotation and, to a lesser extent, lateral bending. Facet joints make a substantial contribution to anterior shear load‐bearing during the initial 2 mm of displacement, and their contribution increases with increasing displacement.^43^ Changes in the orientation of the facet joints affects spine kinematics and mechanics.^44^, ^45^

*Bone*: There is a broad range of bone mineral density (BMD)in vertebrae, which correlates strongly with age,^46^ where BMD and bone mechanical properties decline with increasing age.^23^ These decreases are significant because small differences in BMD can cause significant differences in the likelihood of fracture under load.^46^, ^47^ BMD is a key factor in dictating the failure load of a motion segment.^48^ BMD also affects the mechanical properties of the bone‐implant interface.^48^ An implant may be predisposed to subsidence or loosening if tested in osteopenic bone, as the ultimate strength and fatigue properties of the bone are strongly correlated with BMD.^46^, ^49^, ^50^ Quantitatively, increased bone mineral content has a protective effect whereby a 1 g increase in bone mineral content leads to a 12% increase in cycles to failure.^46^ In torsion, the maximum load at failure of a motion segment is affected by BMD.^25^

##### Sample selection: specimen age

Age has a very significant effect on motion segment mechanical properties.^50^, ^51^ Both static^16^ and dynamic^24^ mechanical properties depend on age. Age also correlates to BMD, which affects the mechanical performance of individual vertebrae and motion segments.^46^, ^52^ Compressive strength of vertebrae is reduced to approximately half in 60‐ to 79‐year‐old specimens relative to 20‐ to 39‐year‐old specimens.^53^ Spinal ligament mechanical properties also correlate to age with a decrease in tensile strength and modulus with increasing age.^36^

The extent of degeneration correlates with age that further affects mechanical properties.^6^ Disc size and height also change with age.^54^ Tensile stiffness of the disc is decreased significantly in 60‐ to 79‐year‐old specimens relative to 20‐ to 39‐year‐old donors.^53^ Fatigue failure of motion segments is also age‐dependent with younger specimens having increased fatigue life relative to older specimens.^46^, ^55^ In anterior‐posterior shear, young specimens creep more than older specimens before failure.^52^ This is important because creep affects stiffness.^52^

##### Sample selection: spinal level

The size, shape, orientation, and proportions of the discs, vertebrae, and facets is dependent on spinal level.^54^, ^56^, ^57^ In vivo, the magnitude of the loads, the motion, and the orientation of the disc at each level of the spine relative to the transverse axial plane is also unique.^58^, ^59^, ^60^ Data from in situ measurements using a spinal stiffness gauge indicated that there is a significant difference in stiffness between levels of the lumbar spine in flexion; L_5_‐S_1_ had the highest stiffness which was significantly higher than L_2_‐L_3_, L_3_‐L_4_, and L_4_‐L_5_.^6^ The variation in stiffness by spinal level has been demonstrated with multiple in vitro studies, both in the lumbar and cervical spine.^11^, ^56^, ^61^, ^62^, ^63^ BMD also varies by spinal level which can affect mechanical properties.^64^, ^65^, ^66^

##### Sample selection: sex

Differences in lumbar motion segment stiffness, range of motion, and hysteresis have been observed with sex. Motion segments from male donors have higher stiffness, lower ROM, and less hysteresis.^6^, ^13^, ^67^ In the lumbar spine, disc area and height are 25% and 15% smaller, respectively, in discs from females than males,^54^ which can affect internal stresses, pressures, and load transfer to the vertebral endplates.^68^ Disc height has a significant effect on the rotational stiffness, with taller discs having lower stiffness.^23^, ^69^, ^70^, ^71^ Cervical discs from male specimens generally fail at higher loads, as much as 25% greater than discs from females.^51^ In the lumbar spine, female motion segments have significantly more segmental motion than male segments.^13^ However, it is unclear whether there are intrinsic differences in mechanical properties between motion segments from male and female donors or whether the observed differences are primarily due to size differences.^13^, ^68^ In the cervical spine, the response to dynamic loading is also sex‐dependent.^51^

Extent and timing of degeneration are also sex‐dependent. Degenerative changes in females lag behind males by approximately 10 years.^72^ The prevalence of endplate lesions is higher in males than females, which can impact disc mechanics as described above.^73^

##### Sample selection: survey results

The majority of respondents (65%) indicated that when investigating therapies for disc degeneration, specimens should be “among a spectrum of healthy to degenerated, the extent of which should be reported for each specimen” (Supporting Information S1, Q29). Almost all respondents (95%) agreed that the extent of degeneration should be reported (Supporting Information S1, Q30). Several respondents indicated that the selection of specimens is often limited by what is available and that selecting specimens with specific properties is prohibitive based on the limited supply.

Respondents indicated that the most important properties to report are donor age (97%), disc level (97%), grade or stage of degeneration (95%), bone mineral density (67%), disc dimensions including height and area (65%), donor weight or body mass index (55%; Supporting Information S1, Q30). A majority of respondents indicated that some specimen properties are important to control including grade or stage of degeneration (90%), disc level (82%), donor age (65%), and BMD (55%; Supporting Information S1, Q31).

#### 3.2.2 Sample Preparation

##### Sample preparation: fresh vs fixed

Autolysis degrades tissues, which will ultimately affect their mechanical properties. Autolysis may be significant during extended exposure times, particularly for high cycle mechanical testing or long‐term creep testing. Fixing of specimens can slow or eliminate autolysis, but the fixation process may significantly alter mechanical properties.

Although neutral buffered formalin does not affect bone mineral content, it does alter the structure of collagen fibers.^74^ Formalin fixation significantly increases stiffness and decreases the range of motion in flexion/extension, lateral bending, and torsion of motion segments by as much as 96%.^75^ Results indicate that biomechanical testing of formalin‐fixed tissue is not representative of in vivo conditions.

More recently, alternatives to formalin fixation have been implemented. Many of these newer fixation techniques have been developed to “feel” like fresh tissue during dissection. Because of the natural feel, it is intuitive that the tissue may also have similar mechanical properties to fresh tissue. Although few researchers would consider using formalin‐fixed tissue for mechanical testing, researchers or surgeons might be tempted to use tissue for mechanical testing that has been fixed with newer techniques. Thiel fixation maintains nonlinear load‐deformation characteristics of motion segments, but increases the ROM and has a destabilizing effect on tissues.^9^, ^76^ Similarly, the “Fix for Life” embalming technique significantly increased motion segment stiffness in all loading directions relative to nonembalmed tissue.^77^

##### Sample preparation: freezing

The effects of freezing and thawing specimens before use have been studied extensively.^78^, ^79^, ^80^, ^81^, ^82^, ^83^ Drying out of specimens during the freeze‐thaw process can alter their mechanical properties, however, wrapping specimens in saline‐soaked gauze and placing them in sealed double plastic bags for freezing mitigates these effects.^78^, ^80^ Passive freezing at −20°C or colder temperature has minimal effect on the elastic properties or dynamic properties of bone and disc tissue.^29^, ^80^, ^81^ While freezing can affect the range of motion, stiffness, and neutral zone of the porcine intervertebral disc after a single freeze‐thaw cycle,^79^, ^82^ the effects of similar protocols are not significant in human specimens.^29^, ^30^ The effects of up to four freeze‐thaw cycles at −20°C are minimal on mechanical properties of fresh‐frozen human cadaveric motion segments.^83^

##### Sample preparation: tissue transection or removal

Harvesting and preparation of specimens often necessitate tissue transection or resection. Specimens are often tested with the posterior elements removed, which is significant biomechanically because there is normally load‐sharing between the disc and facet joints in both the cervical^84^, ^85^ and lumbar spine.^86^, ^87^ Removal of facets in the lumbar spine inherently changes disc biomechanics, resulting in an increase in ROM and decrease in stiffness in flexion/extension, lateral bending, and torsion.^37^, ^38^ Effect of facet removal in the lumbar spine is dependent on the mode of testing,^88^ where the effects of facet joint removal are most prominent in torsion and less so for axial loading.^89^ Removal of facet joints does not significantly affect creep properties of lumbar motion segments,^89^ indicating that the viscoelastic response of a motion segment is primarily dictated by the disc. In the cervical spine, the facet joints are loaded during flexion, extension, lateral bending, and torsion^90^ and the facet joints carry as much as 45% of the force when a motion segment is loaded in axial compression.^91^ Removal of the facets in the cervical spine significantly reduces stiffness in extension and increases range of motion.^91^

Each of the major ligaments (supraspinous, interspinous, ligamentum flavum, intertransverse, posterior longitudinal, and anterior longitudinal) also contributes to the mechanical properties of a motion segment.^92^, ^93^ Transection of the posterior ligaments generally decreases stiffness and increases ROM primarily in flexion.^94^ The anterior longitudinal ligament resists axial rotation and also extension.^94^

##### Sample preparation: potting

To facilitate mechanical testing, specimens must be potted in a way that eliminates relative motion between the specimen and testing apparatus in all six degrees of freedom. Mechanical properties of the potting material can also confound measurements of specimen mechanical properties if the potting material deforms significantly during loading.

Specimens have been potted in polymers, low melting temperature alloys (LMA), or even Plaster of Paris.^2^, ^81^, ^95^ Fast setting epoxies such as Bondo (Bondo Corp., Atlanta, GA) are attractive because they are inexpensive and easy to use. Dental acrylic and poly(methyl) methacrylate (PMMA) are commonly used for potting specimens. One limitation of all of the polymers is that none allow for unpotting and repotting.

Low melting temperature alloys (often referred to as “Wood's Metal”) with melting temperatures as low as 47°C (Cerrolow‐117, McMaster‐Carr Supply Company, Elmurst, IL) minimize thermal necrosis and solidify within minutes. Specimens potted in LMA can be unpotted by rewarming the LMA for reuse.^96^ One limitation of LMA is that it is very dense and can add significant inertia to testing fixtures.

When comparing PMMA, dental acrylic, and LMA, research has shown that filler materials can confound measurements on vertebral body stiffness by more than 9%.^97^One study concluded that LMA is superior for minimizing the confounding effects of potting materials based on its higher modulus of elasticity and repeatability during reuse.^95^ Materials with a higher modulus of elasticity deform less and, in turn, reduce the potential confounding effect of potting material deformation on the overall measurement of the specimen deformation during loading.

##### Sample preparation: survey results

With respect to sample preparation, only 5% of respondents indicated that only fresh samples should be used for testing while 82% of respondents felt that freezing specimens before use was acceptable (Supporting Information S1, Q11). However, 50% of respondents felt that only a single freeze‐thaw cycle was appropriate.

#### 3.2.3 Pre‐testing measures

American Society of Testing and Materials (ASTM) and the International Organization for Standardization (ISO) provides standardized approaches for testing synthetic materials. However, applying these approaches to the spine is challenging and often inappropriate due to variations in specimen anatomy and the condition of the tissues (eg, normal or degenerated). Normalizing specimen anatomy can be achieved by measuring disc height and area prior to testing and can also be used to facilitate load‐ or stress‐controlled protocols; however, limited access to imaging can prohibit disc measurement and can result in inconsistency in testing methods and reporting of specimen properties. Moreover, procedures for procuring and imaging spine specimens may differ depending on whether the researcher has access to fresh tissue and imaging equipment, as may be the case in research labs connected to research hospitals.

Clinical based imaging, such as magnetic resonance (MR) imaging, computed tomography (CT), or X‐ray, are commonly used to assess disc health and can be used to measure disc anatomy. Imaging of specimens after harvest but before testing accounts for the release of residual stresses from spinal ligaments and muscles, which may cause in vitro disc height measurements to be greater than in vivo measurements.^30^, ^98^, ^99^, ^100^, ^101^, ^102^, ^103^ Johnstone et al. showed that fluid content of the inner annulus increases after autopsy compared to discs with the same degenerative grade during surgery, resulting in more uniform intradiscal pressure throughout the disc.^104^

Each imaging modality provides different assessments that may be important for defining mechanical testing parameters or interpreting results. Sagittal plain X‐rays of intact spines are commonly used in the clinical assessment of spine and disc health, where osteophytes and disc height narrowing can be viewed. CT provides a three‐dimensional reconstruction but is not as commonly used for characterizing motion segments for in vitro testing, due to challenges in imaging soft tissues.^105^ CT imaging is more commonly applied in bone research to provide bone mineral density as an assessment of bone quality and strength.^106^

Soft tissues are easier to visualize through MR imaging, with relative disc health being assessed with the Pfirrmann scale.^107^ There is also a growing body of work showing the relationship between disc function and the quality of tissues surrounding the disc, including musculature, and cartilaginous endplates.^28^, ^108^, ^109^, ^110^, ^111^ Specifically, MR imaging has been used to evaluate the relationship between endplate pathology and nutrient diffusion into the disc as well as the relationship with lower back pain.^112^, ^113^, ^114^ Quantitative MR imaging can also provide a biochemical composition, such as water or glycosaminoglycan content.^115^ Quantitative MR imaging with T1ρ‐mapping is able to identify early‐stage disc degeneration,^116^, ^117^ which may be ideal specimens for assessing treatment strategies that aim to prevent the progression of degeneration. Recent work showed that quantitative MR is sensitive to assess changes in water content due to diurnal loading in vivo and mechanical loading in vitro.^118^, ^119^

Regardless of the imaging modality used, disc height and area can be measured prior to mechanical testing. The wedge‐shaped nature of the disc makes it difficult to identify a single point for measuring disc height, resulting in a variety of approaches and significant variations in reported values. For example, human lumbar disc heights can vary from 5 mm in the posterior region to more than 10 mm in the anterior region.^118^

Disc heights taken from two‐dimensional images may be acquired at a single location (eg, center of the disc)^120^ or averaged by outlining the area of the disc space on a 2D image and dividing the area by its anterior‐posterior or lateral dimension in a mid‐sagittal or mid‐coronal image, respectively.^121^ Three‐dimensional images, acquired with MR imaging or CT, can be used to create a planar map of disc height throughout the disc, which can then be averaged.^122^ Researchers without access to noninvasive imaging may use calipers to measure disc height either before testing^79^ or once the disc is removed from the vertebral bodies after testing. This approach is confounded by potential tissue loss during dissection and further reductions in residual stresses which may allow the disc to expand further, thus overestimating the disc height at the beginning of the mechanical test.^20^

##### Pre‐testing measures: survey results

Approximately 60% of survey responders stated that they never or only sometimes measure disc area or height prior to testing, with approximately 35% of responders always measuring disc area and approximately 40% always measuring disc height prior to testing (Supporting Information S1, Q7, Q9). There was no consistency regarding the stage of specimen preparation when imaging was conducted with approximately 25% of responders imaging the intact spine vs those who imaged the prepared motion segment (~35% for disc area and ~ 40% for disc height; Supporting Information S1, Q8, Q10). Depending on the researcher's institution, noninvasive imaging can be quite costly (eg, $600/hour), which may partially explain the lower percentage of researchers who image motion segments prior to testing compared to imaging the intact spine.

#### 3.2.4 Initial conditions

##### Initial conditions: preconditioning

Like all soft tissues, the intervertebral disc exhibits hysteresis with cyclic loading.^123^ Hysteresis is greatest in the first cycle, relative to subsequent cycles.^124^ Once a specimen has completed enough cycles for its mechanical response to loading to become repeatable, it is considered *preconditioned*. Preconditioning tends to increase neutral zone and decrease stiffness relative to the first cycle or few cycles of testing.^125^ Hysteresis is also highly dependent on loading rate; the disc exhibits strain rate dependence, which can affect hysteresis and alter effective stiffness by up to 20%.^81^, ^126^

There are advantages to preconditioning from a study design standpoint in that the properties of preconditioned tissue are reproducible and eliminate the potential confounding effects of cycle number and load history (provided that the number of cycles does not result in additional creep). If the goal of a study is to compare different treatment conditions in the same specimen or across specimens, then preconditioning is advantageous.^127^

There are a number of preconditioning protocols that have been used for spine biomechanical testing. Commonly, for a range of motion testing, two cycles of testing are completed to precondition the specimens before collecting data on the third cycle for analysis.^75^, ^76^, ^77^, ^80^, ^128^, ^129^, ^130^, ^131^, ^132^, ^133^, ^134^, ^135^ However, similar protocols with one cycle of preconditioning,^43^, ^124^, ^136^, ^137^ three to four cycles of preconditioning,^39^, ^138^, ^139^, ^140^ or 10 or more cycles of preconditioning have been reported.^11^, ^141^, ^142^, ^143^ Alternatively, specimens are tested one cycle at a time and the data are analyzed in real‐time to determine if additional cycles of loading are required to produce a repeatable response.^24^, ^140^, ^142^

##### Initial conditions: survey results

The vast majority of respondents (81%) indicated that preconditioning of specimens should be conducted before collecting data for mechanical testing (Supporting Information S1, Q16). Most commonly (33%), respondents indicated that 3‐5 cycles of preconditioning were sufficient. Fewer (17%) indicated that specimens should be preconditioned cyclically until steady state is achieved. An equal number of respondents (12%) indicated that specimens are best conditioned 2 cycles or 6‐10 cycles of loading before collecting data.

##### Initial conditions: preload

Reproducing in vivo spinal loads in vitro remains a challenge.^129^ There is currently no consensus as to the appropriate magnitude or means of applying these physiologic loads in vitro.^144^ The loads developed across the disc space of a motion segment in vivo are the result of three factors: body weight, muscle force, and externally applied loads.^56^ Bodyweight (the weight of the head acting on the cervical spine or weight of the torso acting on the lumbar spine) causes an axial load when the spine is vertical. To account for the forces that result from body weight, axial compressive preloads are commonly applied during in vitro mechanical testing.^1^

The magnitude and direction of applied axial compressive preloads can significantly affect the mechanical and kinematic properties of a motion segment.^145^, ^146^ Axial preloads strongly influence a spectrum of load‐deformation characteristics, including stiffness, ROM, and neutral zone (NZ).^128^ Stiffness and hysteresis of lumbar spine specimens in bending and rotation appear greater at higher axial preloads than lower axial preloads.^89^, ^147^, ^148^, ^149^ In the lumbar spine, the magnitude of preload affects ROM significantly when applying a 0 N vs 200 N vs 400 N axial load. With increasing preload, ROM decreases in torsion.^137^ In degenerated lumbar spines, increasing axial preload results in higher stiffness in all directions of testing.^150^ Both axial compression and distraction cause an increase in torsional stiffness of motion segments up to 150% of the no axial load values.^151^, ^152^ When discs are isolated, preload magnitude affects displacement and stiffness during dynamic loading.^153^ The relative increase in stiffness with compressive preload is nonlinear in the lumbar spine^145^, ^153^ but becomes more linear above 250 N.^151^In the cervical spine, increasing preload results in a decreased neutral zone, higher stiffness at low loads, and better reproduction of in vivo ROM.^62^, ^154^

Multiple techniques have been used to apply axial preloads. However, the method of application of the preload, specifically magnitude and direction, are key to mimicking the in vivo environment.^137^ As first described by Patwardhan, axial loads should be applied tangent to the curve of the spine while passing through the center of rotation of each motion segment.^155^ This paradigm‐shifting methodology, the “follower load,” addresses many of the limitations of other techniques for applying an axial load through the center of rotation of one or multiple motion segments.^155^ Various versions of the follower load technique have been evaluated with variations including fixed upper cups with vertical hanging weights, fixed upper cups with weights guided at the lower cup, and fixed upper cups with weights guided at the disc level.^137^ Results demonstrated that other techniques introduce confounding effects into mechanical testing, but the artifact from the follower load technique was minimal.

While the follower load technique is generally considered to facilitate high fidelity reproduction of in vivo loading, it is often not used in vitro because (a) it is time‐consuming and technically challenging to position the necessary cable guides appropriately, (b) if multiple conditions of a specimen are being tested (ie, intact, injured, and instrumented) the axis of rotation may change for each condition and thus the application of the cable guides must change, and (c) it is impossible to test the same specimens in multiple directions of loading (ie, flexion‐extension and lateral bending). Further, to replicate in vivo physiologic motion, the magnitude of the preload should vary depending on the extent of motion; in the cervical spine, an axial preload is not necessary at the ends of motion but must be maximum near the middle of motion to replicate in vivo motion.^156^

##### Lumbar spine axial compressive preload

The magnitudes of lumbar axial compressive preloads have been indirectly determined by measuring the weight of the body above each level of the spine, during in vivo nucleus pressure measurements,^144^, ^157^ directly measured using telemeterized spinal implants,^158^ or calculated using muscle‐driven musculoskeletal and finite element models.^144^ The appropriate magnitude of axial compressive preload depends on the in vivo scenario being modeled.

In his seminal work in 1950, Ruff determined the fraction of body weight imposed across each level of the thoracolumbar spine.^58^ In this study, the thoracolumbar spines of human subjects were radiographed while standing, and the disc heights were measured. Subjects were then positioned supine and a yoke was applied at the shoulders. Weights were added to the yoke system which caused axial compression of the thoracolumbar spine. With each incrementally increasing weight, the thoracolumbar spine was radiographed, and disc heights measured. Disc heights subjected to applied axial loads in the supine position were compared to disc heights while standing to determine the fraction of body weight at each level of the spine during neutral standing. These data provide magnitudes for axial preload during neutral standing at each level of the spine (Table 1). For a typical American male of weight 900 N,^159^ Ruff's data indicate an axial preload of approximately 540 N at the L_4_‐L_5_ disc.

**TABLE 1:** Axial preload applied to the spine is a result of the weight of the head in the cervical spine and torso in the lumbar spine

Spine	Force
Level	[% BW]
T_5_	21
T_6_	25
T_7_	29
T_8_	33
T_9_	37
T_10_	40
T_11_	44
T_12_	47
L_1_	50
L_2_	53
L_3_	56
L_4_	58
L_5_	60

*Note:* Body weight (BW) fractions above each level of the thoracic and lumbar spine have been determined by Ruff.^58^

The static axial compressive preload can be estimated from upper body weight measurements; however, these can underestimate the actual loads generated from muscle recruitment during dynamic activities. Physiological axial compressive preload magnitudes vary with the type of activity, where minimal compressive loads are present during lying down when compared to sitting, standing, and lifting activities (Table 2).^144^

**TABLE 2:** Compressive loads to replicate physiological loading

	Applied load (N)		Stress (L4L5)
	L3L4	L4L5	MPa
Lying supine	100	106	0.06
Sitting slouched	270	286	0.16
Sitting relaxed	460	488	0.27
Standing	***500***	***530***	0.29
Sitting with actively straightening back	550	583	0.32
Mid‐range during walking	590	625	0.34
Holding 20 kg close to body	1100	1166	0.64

*Note:* in vitro testing by Dreiscarf et al determined the load needed to replicate intradiscal pressures as measured by Wilke et al.^129^, ^153^ Values reported by Dreiscarf et al were used as the baseline values for L_3_‐L_4_ and L_4_‐L_5_ discs (bold values). Data from Wilke et al were used to calculate the relative difference in activity. Finally, the applied stress for the L_4_‐L_5_ disc was calculated by using an average disc area of 1826 mm^2^.^99^, ^121^

While a 400 N axial preload is commonly applied to the lumbar spine during in vitro ROM testing, a range of loads have been used depending on the in vivo loading condition being replicated. Axial compressive preloads may range from 0 to 250 N,^23^, ^145^, ^160^, ^161^, ^162^, ^163^, ^164^, ^165^, ^166^ 350 to 500 N,^11^, ^43^, ^55^, ^126^, ^137^, ^142^, ^145^, ^148^, ^165^, ^167^, ^168^ and greater than 500 N.^26^, ^55^, ^92^, ^95^, ^150^, ^151^, ^155^, ^167^, ^169^, ^170^, ^171^, ^172^, ^173^ For conditions to simulate in vivo bending of the lumbar spine, axial compressive preloads above 500 N are generated^144^ depending on disc cross‐sectional area. Low magnitude axial loads (0‐250 N) may simulate lying down, but several studies have applied pure moments with no axial compressive preload.^39^, ^134^, ^137^, ^174^, ^175^, ^176^, ^177^, ^178^ In other studies, the decision on the magnitude of preload is often without justification.^23^, ^43^, ^55^, ^137^, ^142^, ^160^, ^161^, ^162^, ^164^, ^166^

##### Lumbar spine axial compressive preload: survey results

From the survey responses, 74% of researchers stated that applying an axial compressive preload was either absolutely critical or somewhat important with a higher preference for absolutely critical (Supporting Information S1, Q17). Less than 10% reported that applying a preload was either somewhat unimportant or not important at all. Almost 20% chose “Other” where most indicated that it depended on the research/clinical question. For the magnitude of lumbar spine axial preload, most researchers surveyed would apply between 351 and 500 N (29%), followed by 251‐300 N (25%), 0‐250 N (20%), and greater than 500 N (14%; Supporting Information S1, Q18). All researchers supported the application of a preload. Of the 12% of responders who chose “Other”, their comments suggested that the magnitude of the preload depended on the research question, the disc area, the donor's bodyweight, or should be on data from studies that measured the in vivo nucleus pressure.

##### Cervical spine axial compressive preload

The biomechanical protocols for ROM testing in the cervical spine also vary from study to study. Critical parameters such as axial preload and magnitude of applied forces and applied moments vary widely across different studies with no universally accepted protocol. Some specimens have axial compressive preloads applied while others do not. The magnitude of axial preload ranges from 0 to 125 N. Some axial loads are applied using the follower load concept and others are not.

In the cervical spine, axial preload ranges from 20 to 50 N.^139^, ^146^, ^179^ To simulate physiologic loading conditions, axial loads have ranged from 50 to 125 N. The lower forces (50 N) represent axial loading from the weight of the head and neck in the neutral position, whereas the higher forces (100 N and greater) represent axial loads due to the weight of the head and muscle forces during physiologic motion.^180^ Recently, ROM testing of the cervical spine with a follower load of 100 N and an applied moment of 2.0 Nm in flexion‐extension demonstrated the highest fidelity and reproducibility relative to in vivo range of motion when compared to other combinations of preloads and applied moments.^180^, ^181^

##### Cervical spine axial compressive preload: survey results

The survey results found that most researchers (42%) applied a cervical axial compressive preload of between 51 and 150 N, followed by 0‐50 N (29%) and 151‐250 N (9%; Supporting Information S1, Q19). Eighteen percent chose “Other”, however, half of these indicated that they have not tested cervical spines before, and the remaining half recommended considering the disc area, donor bodyweight, and the research question.

#### 3.2.5 Testing environment

##### Testing environment: time and temperature

Environmental exposure time affects the mechanical properties of the spine.^2^, ^80^Increasing exposure time to an ambient temperature significantly alters motion segment ROM. Data from the lumbar spines of pigs and sheep demonstrate ROM increases of 30%‐50% with ambient exposure up to 72 hours, although the increase is less than 10% in the first 10‐20 hours.^2^ Storage in a refrigerator between thawing and testing reduces the effects of exposure time and results in minimal changes in tissue properties up to 14 days of refrigerated storage.^80^

The effect of time‐temperature of exposure of the human thoracic spine was quantified by Panjabi as 0.009 mm/day of change in translation and 0.022°/day change in rotational ROM.^80^ The effects of environmental exposure are amplified at higher temperatures. At 37°C, the cellular autolytic processes are accelerated, and the specimen's biomechanical properties are compromised rapidly reducing the viable testing window. ROM, neutral zone, stiffness, and hysteresis are all affected by testing temperature. In the lumbar spine, both ROM and neutral zone are reduced at room temperature testing relative to body temperature.^11^ This is particularly significant in axial rotation but less significant in flexion and extension.^142^ In axial rotation, motion segments demonstrate a significantly reduced stiffness at body temperature relative to room temperature.

To help preserve specimen longevity with minimal changes to tissue mechanical properties during long‐duration or elevated temperature testing, protease inhibitors are added to a hydrating solution.^182^ Protease inhibitors reduce the rate of putrefaction and tissue autolysis and are often used during long‐term testing.^126^, ^183^, ^184^, ^185^

##### Testing environment: hydration

Disc mechanical properties are dictated in part by its internal osmotic pressure, which is dependent on the salt‐based or sugar‐based hydrating solution used.^21^, ^186^, ^187^, ^188^ Exposure to ambient air can affect disc hydration, so measures to mitigate dehydration are critical for minimizing confounding effects of tissue hydration during testing.^2^, ^124^, ^189^ Maintenance of physiologic hydration is particularly challenging in long‐duration tests, such as high cycle or creep testing. With increasing exposure to air (without rehydration), porcine and ovine lumbar motion segment stiffness is reduced and ROM is increased.^2^, ^133^ Likewise, hyperphysiologic swelling of human lumbar motion segments can increase disc water content and confound mechanical testing results.^78^, ^124^ While there is a substantial body of research on the effects of disc hydration on in vitro mechanical properties, there is no universally accepted protocol for maintaining physiologic hydration.

Wrapping specimens in saline‐soaked gauze, spraying or dripping irrigation on specimens, conducting testing in 100% humid environments, or testing specimens within a hydrating solution are all methods to minimize the effects of air exposure.^2^ When specimens are maintained in a 100% humid environment, the change in water content of the disc is minimal during testing.^124^ Submerging specimens in a bath is also effective at reducing dehydration. However, if specimens are allowed to swell unconstrained, the hydration becomes hyperphysiologic.^124^, ^190^ This can occur in as little as 1 hour of immersion.^191^ Hyperphysiologic tissues absorb more energy than physiologic tissues which can alter their dynamic properties and stiffness.^190^, ^191^

Submerging tissue in saline while applying a constraining load (or first submerging tissue unconstrained then applying a compressive load) allows for tissue hydration while minimizing the likelihood of hyperphysiologic swelling.^189^ However, the duration of immersion and the magnitude of the constraining loads that have been used varies widely. With a broad range of justifications, lumbar motion segments have been immersed in saline with axial loads ranging from 150 to 500 N,^43^, ^52^, ^82^, ^192^ immersed in saline under axial stresses ranging from 0.1 to 1 MPa compression,^26^, ^143^, ^189^ immersed in saline unconstrained,^78^, ^138^, ^149^, ^153^, ^182^ or immersed in water unconstrained.^139^ The osmolarity of the bath, which is often not considered, may greatly alter fluid flow into and out of the disc, impacting measured mechanical properties. A recent study showed that hydration in saline may not be appropriate for maintaining swelling of excised tissues, whereas adjusting saline bath osmolarity with polyethylene glycol (PEG) may restrict fluid flow into biological tissues, such that the water content remains comparable to fresh tissues.^187^

A specimen's load history also affects its hydration, altering disc height, disc volume, and mechanical properties.^119^, ^193^ Compressive properties measured during short‐duration tests (<90 minutes) are not affected significantly by the testing environment (ie, air, saline‐soaked gauze, or submerged in a bath),^193^ but long‐duration tests in the air do result in an increase in stiffness when compared to tests performed in a bath.^191^ Moreover, if specimens are evaluated using protocols that include multiple loading conditions (eg, dynamic loading or compression, bending, rotation, etc.), the change in disc anatomy will alter normalized mechanical properties as evaluated using classical mechanics, such as strain, stress, and, therefore, modulus. Immersion in saline between test cycles improves disc recovery between tests.^194^

##### Testing environment: survey results

Like the wide range of current practices reported in the literature, survey results were variable with respect to testing environment. A majority of respondents (61%) indicated that testing at 37°C is more physiologically relevant than room temperature testing, however, 17% indicated that there is no difference in relevance between room temperature and 37°C testing (Supporting Information S1, Q12).

A majority of respondents (62%) indicated that specimens should be kept moist during testing with wet gauze or spray and 7% preferred testing in a 95% humid environment for the maintenance of hydration (Supporting Information S1, Q13). Interestingly, only 22% indicated that immersion in a bath was most appropriate for specimen hydration. For respondents who submerge specimens in a bath, 77% indicated that they apply a preload (Supporting Information S1, Q14). With respect to hydration solution, 94% of respondents use saline (Supporting Information S1, Q15).

#### 3.2.6 Test conditions

##### Lumbar spine testing strategies

The goal of in vitro biomechanical testing is to evaluate the response of specimens, both intact and after various interventions, in a manner that approximates in vivo performance.^195^ The closer in vitro biomechanical testing of the spine simulates in vivo loading regimes, the more confident we can be when evaluating the biomechanical response of the native segment, its treatments and future tissue repair, replacement, and regeneration strategies. This goal is currently unachievable due to unknown in vivo 6DOF force and moment magnitudes, and due to limitations of testing systems to reproduce in vivo conditions.^2^, ^3^, ^171^, ^196^, ^197^

The application of pure moments^198^ during load‐controlled testing vs displacement‐controlled testing has been the subject of much debate^199^ over the past 40 or more years. A pure moment is a pure rotation that applies only torque to a specimen without any axial or shear loads and can be applied independently or in combination with other loads, such as axial compression. Applying individual unconstrained pure moments does not simulate in vivo loads,^2^ however, this technique presents a method for standardized testing for comparison across laboratories.^2^, ^200^ Displacement‐controlled testing may more closely replicate the measured in vivo translations and rotations of vertebrae about a fixed axis of rotation,^199^, ^201^, ^202^ however, this may also introduce nonphysiological coupling effects.^199^ The testing apparatus required for the application of pure moments is often considered to be more straightforward for developing in‐house when compared to the testing systems required to replicate complex 6DOF in vivo translations and rotations. There is substantial work based on both the application of pure moments, either with axial loads^128^, ^136^, ^137^, ^142^, ^150^, ^155^, ^160^, ^161^, ^163^, ^164^, ^166^, ^167^, ^168^, ^171^, ^179^, ^197^, ^203^, ^204^, ^205^, ^206^ or without axial loads,^7^, ^39^, ^63^, ^134^, ^137^, ^162^, ^174^, ^175^, ^176^, ^177^, ^207^, ^208^, ^209^, ^210^, ^211^, ^212^, ^213^, ^214^ and with using displacement control/stiffness test methods also being commonly used.^23^, ^32^, ^43^, ^125^, ^126^, ^148^, ^165^, ^215^, ^216^, ^217^, ^218^

There have been many novel methods and systems developed to facilitate testing under load‐control and displacement‐control, and some have represented paradigm shifts in advancements towards replicating physiological loading. A “universal spine tester” was developed by Wilke et al,^219^ which represented a significant breakthrough in continuous (~1°/s),^220^ unconstrained 6DOF pure moment testing where single or multiple spine segments could be loaded in each DOF without repositioning. This system included the ability to independently apply muscle forces, either unilaterally or bilaterally during the simultaneous application of 6DOF loads. Patwardhan et al introduced a paradigm shift for the application of larger magnitude, more‐physiological preloads, in the form of a compressive follower load, particularly for increasing the load‐carrying capacity of the whole lumbar spine without buckling.^155^ Before these studies, new developments in the application of a combination of load and displacement (hybrid) control strategies were employed for 6DOF testing of knee joints using a serial (articulated) robot manipulator.^221^ Gilbertson et al further developed this hybrid control strategy for spine segment testing, based on measuring the specimen stiffness in” real‐time”, to identify the path of passive motion (ie, least resistance and zero off‐axis forces/moments) and follow the segment's center of rotation.^215^ This control strategy essentially replicated unconstrained, pure moment testing systems, and has been implemented by others, all of which operate at quasi‐static speeds.^204^, ^205^ Using these techniques, the kinematic path was recorded and could be played back in position control for testing at faster speeds.

Another paradigm shift in techniques for 6DOF testing was the development of a novel Stewart platform, or parallel (hexapod) robot by Stokes et al.^148^ Spine segments were placed inside the workspace, as opposed to on top, as used with conventional platforms. The design allowed a fluid bath to be conveniently fitted around the inferior specimen mounting pillar. The robot primarily operated in 6DOF position control with the ability to apply load control in 1DOF. The Stewart platform concept is well known for its high load carrying capacity, good dynamic performance, precise positioning, and high structural stiffness when compared to serial robots, at the expense of a smaller overall envelope of motion.^222^ For the first time, Thompson, Barker, and Pearcy used a serial robot to more closely simulate in vivo physiological lumbar segment kinematic motion through their ROM,^201^ and about their IAR,^202^ as measured in humans.^125^ Displacement control was used to apply the in vivo kinematic motions in flexion‐extension, lateral bending, and axial rotation.

In 2007, Panjabi developed a “Hybrid” test method where an unconstrained pure moment was first applied to the intact spine and the total ROM measured, then after an intervention, an unconstrained pure moment was applied until the same intact total ROM was achieved. This method allowed for the standardized comparison of the kinematics of each vertebra between the intact and treated states to study adjacent level effects.^223^, ^224^

Goertzen and Kawchuk used a commercial parallel robot (hexapod) to implement a novel velocity‐based force control approach for unconstrained 6DOF spine testing.^208^ Velocity‐based force control is commonly used in serial robots and had not previously been implemented for biomechanical testing. This control was performed at a constant angular velocity of 0.25°/s, which is quasi‐static. Lawless et al, in 2014, developed a new approach to achieving closer to real‐time unconstrained 6DOF load control^197^ using a custom‐developed hexapod robot.^225^ This system used an adaptive velocity‐based 6DOF load control strategy together with the simultaneous, independent application of a compressive preload. This improved control system achieved unconstrained 6DOF load control at speeds of up to two orders of magnitude greater than previous systems with continuous loading at rates up to 4.8 Nm/s. In 2016, Wilke et al developed a 6DOF dynamic testing system capable of applying very fast angular velocities of 707 °/s in position control in lateral bending and axial rotation, and 400 °/s in flexion‐extension.^171^ These speeds are appropriate for studying the sudden overload failure mechanisms of the disc, such as during herniation and endplate failure during combined, complex loading.^226^, ^227^

##### Lumbar spine testing strategies: survey results

Seventy‐five percent of all researchers identified the importance of applying pure bending to specimens during in vitro ROM testing as being either “absolutely critical” or “somewhat important,” with a relatively even split between each (Supporting Information S1, Q20). Ten percent of researchers chose “somewhat unimportant” or “not important at all.” Fourteen percent chose the “other” category, where half of these respondents noted that their decision depended on the research question or that they used displacement control tests.

When surveyed on how loads should be applied during bending and rotation testing, researchers revealed that the application of pure bending moments combined with axial loads was most common (58%), followed by 23% who chose other methods (Supporting Information S1, Q21). Within the other methods, three‐quarters suggested that the way in which loads should be applied depended on the research question with the remaining respondents using displacement control/stiffness test methods. Sixteen percent chose pure moments with no combined loading.

Survey responses for the best techniques for applying bending and rotation found that the majority of responders (41%) recommended either load or position control, depending on the task (Supporting Information S1, Q22). The next ranked recommendation was by a combination of load control and position control (30%). Only a small minority of respondents indicated that load control only (14%) or position control only (11%) were appropriate.

##### Lumbar spine bending moment

The magnitudes of bending moments that are produced in vivo in the lumbar spine remain unknown. Estimates of physiologic bending moments have been indirectly determined by EMG‐assisted kinematic chain models,^228^, ^229^, ^230^ measured using telemeterized spinal implants,^231^, ^232^, ^233^ or calculated using finite element models.^234^

While the magnitude of in vivo moments has never been directly measured, the magnitudes of in vivo vertebral rotations and their instantaneous centers of rotation have been determined.^202^, ^235^, ^236^, ^237^ Using these in vivo rotational data, in vivo moments can be estimated through in vitro testing. Using 6DOF load control systems and testing at near‐physiological loading rates^26^, ^197^, ^225^ with physiologically relevant axial preloads,^202^, ^238^ and with hydration at 37°C,^26^ estimations of bending moments can be made. For example, the lateral bending stiffness of mildly degenerated cadaver lumbar motion segments (L_1_‐L_2_, L_3_‐L_4_) using the above‐described loading regime, under an average axial compressive preload of approximately 600 N (to create an 0.5 MPa equivalent in vivo intradiscal pressure), when rotated 3° in one direction, was approximately 3 Nm/°.^26^ For similar specimens, another study reported a stiffness of 1.2 Nm/° but had applied a pure moment of 7.5 Nm and an axial preload of 440 N.^11^ At a lateral bending angle of 3°, the applied moment would need to be 9 Nm in the first study^26^and 3.6 Nm in the second study.^11^

Using a finite element model of the whole lumbar spine, Dreischarf et al^170^ determined, based on comparison to in vivo intradiscal pressure measurements, that the best agreement with *in vivo* values was a moment of 7.8 Nm under a 700 N compressive follower load for maximum physiologic loading in lateral bending.

Based on these indirect measurements, applied bending moments for in vitro lumbar spine mechanical testing range from 2.6 to 7.5 Nm^11^, ^23^, ^128^, ^137^, ^142^, ^164^, ^174^, ^177^, ^178^, ^210^, ^239^ and 7.6 to 10 Nm.^7^, ^134^, ^137^, ^155^, ^160^, ^161^, ^176^, ^215^, ^240^ Some studies have applied greater than 10 Nm,^39^, ^150^, ^166^, ^167^ and other studies measured bending moments while conducting tests in position or hybrid position‐load control.^8^, ^26^, ^32^, ^95^, ^125^, ^126^, ^148^, ^165^, ^172^, ^185^, ^216^, ^241^

##### Lumbar spine bending moment: survey results

The majority (50%) of survey respondents suggested that applying between 2.6 and 7.5 Nm of the bending moment would mimic physiological loading, followed by 29% recommending applying between 7.6 and 10 Nm (Supporting Information S1, Q23). Thirteen percent chose “Other” where responders indicated that the magnitude of applied bending moment should depend on the research question, or be scaled based on anthropometric parameters, or that the tests should be conducted under position control for achieving realistic motion, or that physiological loading cannot be replicated without including the contributions from muscles.

##### Cervical spine bending moment

The techniques used to load the cervical spine in flexion, extension, lateral bending, and torsion are highly variable. Techniques range from pure moment application,^84^, ^242^, ^243^, ^244^, ^245^, ^246^ to pure moment with axial load,^247^, ^248^ to axial load only,^244^ to combined bending with compression.^175^, ^249^, ^250^, ^251^, ^252^, ^253^ Testing apparatuses range from those that facilitate unconstrained motion^254^ to techniques where specimen motion is limited by the testing apparatus^247^, ^251^ and may introduce artifacts into the ROM measurement.^37^ Like the lumbar spine, some specimens are loaded with pure moments^214^, ^242^, ^243^, ^244^, ^255^, ^256^ while others are loaded using combined compression/bending protocols where the magnitude of the compression and the magnitude of the bending cannot be independently controlled.^175^, ^249^, ^250^, ^251^, ^252^ Magnitude of applied moments ranges from 1.0 Nm^249^, ^250^ to 5.0 Nm.^251^ Axial preloads typically range from 0 N (no axial load) to 125 N^156^, ^181^, ^257^ and have been as high as 300 N.^258^ ROM testing with a follower load of 100 N and applied moments in flex/extension of 2.0 Nm demonstrated highest fidelity and reproducibility relative to in vivo measurements.^180^, ^181^

##### Cervical spine bending moment: survey results

For applied cervical bending moment magnitude, the majority of respondents would apply between 1.6 and 2.5 Nm (53%), followed by 2.6‐3.5 Nm (15%), 0‐1.5 Nm (9%), and greater than 3.5 Nm (6%; Supporting Information S1, Q24). As for the preload survey responses, half of the 17% in the “Other” category have not tested cervical spines, and the remaining half suggested 5 Nm, position control testing, scaling by donor bodyweight, and the research question.

#### 3.2.7 Cyclic testing

Monotonic testing is commonly used as a means to assess disc mechanics. However, monotonic loading has limited fidelity with respect to physiological loading conditions. Early spine biomechanics studies used cyclic loading to assess disc “fatigue” properties. From a mechanics perspective, fatigue loading refers to applying cyclic loading until failure. Achieving, a disc failure in vitro is a significant challenge, often requiring hyper‐physiological loads and moments.^55^, ^172^, ^259^, ^260^ Therefore, what is commonly described as “fatigue” loading is more descriptively extended or high cycle loading.^261^ Dynamic properties of the disc have been assessed under single and multiple loading modalities, including tension‐compression,^99^, ^262^ compression‐only,^261^, ^263^ flexion or flexion‐extension bending,^264^, ^265^ and torsional loading with or without compression.^24^, ^89^ The use of multiple loading modalities provides a closer representation of in vivo loading during activities of daily living.

Differences in testing protocols (eg, frequency used, or applying load‐ or displacement‐control) increases complexity in comparing findings between studies. Often load‐controlled protocols are applied when disc height is not known a priori, with loading rates from 0.0005 to 5 Hz, which is roughly equivalent to 1‐10 kN/s.^120^, ^126^, ^189^, ^193^, ^266^ In vivo, the rate and frequency of disc loading are highly variable and activity‐dependent. Walking is often used as a baseline for guiding cyclic loading protocols, where the average walking pace has been shown to range between 1.4 and 2.1 Hz.^267^ This may be doubled for spine loading (eg, the spine is loaded for foot strike on both the left and right sides). Loading rates between 0.5 and 5 Hz has been shown to have relatively small impacts on disc stiffness (<5%).^120^, ^193^ However, differences in disc stiffness measurements have been observed when testing at hyper‐physiological or hypo‐physiological loading rates.^126^, ^189^

As described with preconditioning, hysteresis stabilizes by the third cycle, with less than 2% change in stiffness and hysteresis for subsequent cycles.^193^, ^261^ However, nonfailure properties, such as storage and loss modulus, have been evaluated over a wide range of loading cycles, with analysis being performed up to the 50th cycle.^182^, ^261^, ^263^, ^268^, ^269^ While relatively small changes are observed from one cycle to the next, creep deformation accumulates with each dynamic loading cycle(ie, accumulation of <2% change between cycles),^193^ which can confound comparisons between studies.

Due to the nonlinear behavior of the disc, multiple approaches have been developed for data analysis (eg, calculating neutral zone and linear region stiffness).^269^, ^270^ Limited consensus regarding data analysis methods has been shown to cause significant differences in reported values.^271^ Calculating a neutral zone may not be clinically relevant, as this is the point in vitro where the disc has zero resistance to load between tension and compression. The magnitude of preload also dictates the linearity of the response to dynamic loading. Studies that have evaluated axial rotation and 6DOF observed more linear behavior (or pseudo‐nonlinear) when a larger compressive preload was applied.^145^, ^149^, ^151^, ^272^

For long‐duration cyclic loading or fatigue loading, faster loading rates have been used,^263^, ^264^ but the relevance of hyper‐physiologic rates is questionable. Although higher loading rates shorten the testing time for failure testing (eg, within ~1000 cycles), increasing loading frequency from quasi‐static to hyper‐physiological causes a 5‐ to 6‐fold increase in stiffness.^189^ Moreover, disc joint (vertebra‐disc‐vertebra) failure at higher frequency has been shown to include both vertebral body failure in addition to endplate failure, which is more commonly observed at lower frequencies (<2 Hz or 3000 N/s).^172^, ^266^, ^273^

#### 3.2.8 Viscoelasticity

##### Viscoelastic loading

The intervertebral disc is a viscoelastic composite structure with time‐dependent mechanical properties, but few studies concurrently measure both static and dynamic properties. However, a limited number of studies have measured disc behavior under both conditions.^103^, ^262^, ^269^, ^274^ In vivo, the disc is subjected to some amount of compression throughout the diurnal loading cycle, due to the weight of the body and muscle engagement, making creep testing relevant to in vivo biomechanics. However, studies that evaluated creep‐recovery behavior showed that creep mechanics differ between the first cycle and subsequent cycles.^275^ This suggests that creep loading protocols may also need a preconditioning phase of either cyclic loading or multiple creep‐recovery cycles before the disc response achieves a steady‐state condition.^30^, ^113^, ^275^, ^276^, ^277^ Multiple creep‐recovery cycles essentially acts as a low‐frequency cyclic loading test with a square waveform.

Similarly, hold times for creep tests vary significantly, from 5 minutes to 24 hours (Table 3). For human discs, very long duration creep tests rarely achieve creep displacement equilibrium in vitro (eg, greater than 8 hours),^278^ which differs from findings with healthy bovine discs that have reported equilibrium after 15 hours of loading.^279^ However, achieving intradiscal pressure equilibrium in vitro required an additional 20 hours for the internal pressure to decrease towards 0 MPa.^279^

**TABLE 3:** Summary of studies that applied axial disc compression

	Time (hrs)	Applied load (N)	Applied stress (MPa)	Species	Comparable in vivo activity
Adams^260^	4		***	Human	Body weight (BW)
Koeller^281^, ^282^	0.08	950	*0*.*52*	Human	Holding <20 kg near body
Kolditz^283^	24		0.60	Human	Holding 20 kg near body
			1.20		High Loading
Keller^28^	0.5	***	***	Human	Adjusted by BW
Ohshima^284^	24	49‐294	0.06‐0.34	Porcine	Lying supine to walking
Holmes^285^	0.5‐6	1600	0.88	Human	High loading
Li^286^	1	***	***	Human	Adjusted by BW
Ekstrom^287^	0.22	50	0.06	Porcine	Lying supine
		100	0.11		Less than slouched sitting
Riches^288^	0.33		1.00	Human	High loading
Palmer^289^	0.33		0.40	Murine	Mid‐range during walking
			0.80		High loading
Sarver^290^	0.5	0.25	0.25	Murine	Sitting relaxed
Boxberger^122^	0.75	4.5	*0*.*28*	Rat	Standing
Johannessen^262^	2	200	0.50	Ovine	Holding <20 kg near body
Heuer^130^	0.25	500	*0*.*27*	Human	Sitting relaxed
Luo^111^	2	1000	*0*.*55*	Human	Holding <20 kg near body
Masuoka^103^	0.15		1.00	Rat‐tail	High loading
	1.5				
	15				
O'Connell^121^, ^291^, ^292^	0.33	1000	*0*.*55*	Human	Holding <20 kg near body
Korecki^293^	1		0.20	Bovine	Sitting
Barbir^274^	0.5	12.5	*0*.*78*	Rat	High loading
Pollintine^278^	0.5	1150	0.85	Human	High loading
	1				
	2				
Campana^294^	0.17	400	0.27	Human	Sitting Relaxed
Hwang^295^	1.67		1.00	Rat‐tail	High Loading
Holguin^296^	1	6	0.37	Rat	Mid‐range during walking
Martin^297^	1	1.5	1.19	Murine‐tail	High Loading
van der Veen^298^	24		0.80	Human	High Loading
Bailey^299^	0.33		0.50	Murine	Holding <20 kg near body
Pei^300^	0.08	200	0.32	Ovine	Sitting with straight back
		600	0.95		High Loading
		1000	1.59		High Loading
Bezci^190^	4	200	*0*.*41*	Bovine	Mid‐range during walking
		1000	*2*.*04*		High loading
Schmidt^277^	8		0.50	Bovine	Holding <20 kg near body
Russo^141^	1		0.25	Ovine	Sitting Relaxed

*Note:* For studies that did not report stress, applied stress was calculated by using either the average disc area reported in the paper or species‐specific disc area from data in O'Connell et al and/or Beckstein et al (italicized stress values).^99^, ^121^ Applied stress was then compared to in vivo loading conditions using data provided in Table 2. Bovine discs are acquired from the caudal region of the spine due to cuts made in the lumbar spine for the meat industry. All other discs were taken from the lumbar region unless specified (eg, for rat or mouse).

There is also a range of applied load magnitudes used during creep testing (Table 3). Often load‐control protocols are employed due to challenges in measuring disc geometry a priori; therefore, disc area or applied stress is rarely reported, making it difficult to compare between studies (Table 3, italicized stress values). Alternatively, if disc area and height can be measured a priori, stress‐controlled protocols can be employed and based on physiological activities (Table 2).

Creep stress is reported in the range from as low as 0.06 MPa, which is equivalent to the intradiscal pressure experienced while lying in a supine position, to over 2 MPa, which is greater than expected in vivo pressure but not high enough to cause damage (Table 2).^238^, ^280^

##### Viscoelastic recovery

While creep testing has mainly been used to evaluate fluid flow out of the disc, there has been a shift towards using similar techniques at low loading conditions to investigate the recovery behavior of fluid flow into the disc.^288^, ^301^ Because disc behavior is dependent on loading history, disc recovery will be affected by the creep loading protocol that was applied (magnitude and duration). A recent study with healthy bovine discs showed that disc recovery from creep was dependent on the magnitude of applied load, where the initial elastic recovery behavior was greater for discs that experienced higher loads during creep.^302^

Stress magnitudes applied during recovery range from no loading (0 MPa) to 0.04 MPa, which is slightly lower than the estimated stress on a lumbar disc during supine lying (Table 4).^29^, ^30^, ^275^, ^276^, ^301^, ^302^, ^303^, ^304^, ^305^, ^306^ Studies on disc recovery face similar challenges with reaching equilibrium, where full disc height recovery is often not achieved in 0.15 M phosphate‐buffered saline, even after 24 hours of unloaded or low‐load recovery.^275^, ^302^ Recent studies have shown that the recovery environment and previous loading history will greatly alter disc recovery mechanics, and the ability to achieve equilibrium during recovery.^303^, ^307^ That is, disc recovery does achieve equilibrium when under higher osmotic conditions that prevent fluid flow into biological tissues, but the direct representation of in vivo osmotic loading remains unknown.

**TABLE 4:** Summary of studies that applied axial compression to disc joints, followed by recovery

	Time (hr)	Applied load (N)	Applied stress (MPa)	Species	Comparable in vivo activity	Recovery time (hr)	Recovery load (N)	Recovery stress (MPa)	Comparable in vivo activity
Burns^301^	8	178	*0*.*10*	Human	Less than slouched sitting	16	44	0.02	Less than lying
Bass^30^	0.33	100	0.11	Porcine	Less than slouched sitting	0.67	0	0	Not comparable (NC)
Dhillon^29^	0.33		1.00	Human	High Loading	0.67	0	0	NC
MacLean^306^	4	2.5	0.20	Rat‐tail	Sitting	6		0.04	Supine lying
van der Veen^276^	0.25		2.00	Porcine	High Loading	0.5		0.001	NC
Hsieh^305^	0.25		0.30	Rat‐tail	Standing	0.5		0.003	NC
Chuang^304^, ^308^	1	750	1.01	Bovine	High Loading	24		0	NC
O'Connell^275^	4	1000	*0*.*55*	Human	Holding <20 kg near body	Up to 24	20	0.01	Less than lying
Bezci^303^	2	300	0.61	Bovine	Holding 20 kg near body	12	20	0.04	Supine lying
Bezci^302^	24	100‐1200	0.15‐2.00	Bovine	Sitting to High Loading	18	10	0.02	Less than lying

*Note:* For studies that did not report stress, applied stress was calculated by using either the average disc area reported in the paper or species‐specific disc area from data in O'Connell et al and/or Beckstein et al (italicized stress values).^99^, ^121^ Applied stress was then compared to in vivo loading conditions using data provided in Table 2. Bovine discs are acquired from the caudal region of the spine due to cuts made in the lumbar spine for the meat industry. All other discs were taken from the lumbar region unless specified (eg, for rat or mouse).

For analyzing creep data, often rheological models are used to curve‐fit to experimental results. These models may use three to five parameters, which do not have physical interpretation, to describe the overall nonlinear response.^28^, ^262^, ^302^ Since these tests are not likely to achieve equilibrium, the model parameters are useful for comparing data between groups, but the models will likely overestimate predictions of equilibrium, due to insufficient data.^298^

##### Cyclic and viscoelastic testing: survey results

There was a strong consensus in the survey that mechanical properties from both static and dynamic loading are equally important (70% of responders; Supporting Information S1, Q25). Approximately one‐third of survey responders stated that they did not have prior experience with static (either creep or stress‐relaxation) loading (Supporting Information S1, Q26). Of survey respondents with static loading experience, there was no consensus regarding the testing time (Supporting Information S1, Q27). Approximately 30% of responders stated that tests should be conducted for up to 2 hours, while 39% of responders suggesting tests be conducted 8 hours or longer, which reflects the lack of consensus in the literature (Tables 3 and 4).

For study designs that require the same specimen to be tested repeatedly, 83% of respondents indicated that specimens should be rehydrated between testing (Supporting Information S1, Q32). Approximately 50% of survey responders stated that specimens should be rehydrated for a specified amount of time before retesting the specimen. Of respondents, 34% indicated that specimens should be rehydrated until disc height has recovered.

#### 3.2.9 Study design

In addition to the above reported findings, which are highly relevant for experimental spine biomechanics, there are many study design aspects that are common for all scientific research, which also applies to spine mechanics.^309^ The importance of designing a study that is adequately powered based on a priori sample size calculations, and utilizes a repeated measures study design (each sample acts as its own control for normalizing data), if feasible, are critical for reducing sample size and the chances of false‐negative findings.^310^, ^311^ Estimations of effect sizes for a priori analyses is challenging without in‐house pilot data or from equivalent studies in the literature.^311^ Of greatest challenge is the determination of clinically relevant effect sizes, which may be of a different magnitude when compared to in vitro data.^6^ When no significant differences are found, post hoc power analyses are critical for determining whether the study is underpowered. Finally, an analysis of repeatability of methodologies, within or between laboratories is important to consider.^168^, ^177^, ^193^, ^311^ A number of these aspects were explored in the survey, which are summarized below.

##### Study design: survey results

For the best approach to reporting the effect of a treatment on segment mechanical properties, most survey responders (50%) chose a repeated measures (paired) study design where the treatment data were normalized to the same specimen in its intact state (Supporting Information S1, Question 28). The next common choice (22%) was to normalize data from the treatment group to an untreated control group. Twelve percent chose not to normalize and just report the raw data. Another 9% wrote in other choices that emphasized the need to report both the raw data and data normalized to both the treatment and intact condition.

For eliminating bias from testing history, most respondents (43%) chose to randomize testing order (Supporting Information S1, Q33). However, 36% chose to use a control group to undergo repeat testing without injury/treatment if randomization cannot be used. Ten percent would increase their sample size to minimize bias, and 7% chose “Other” where they supported combinations of the two most preferred options and included conducting an a priori power calculation.

With regards to conducting an a priori power analysis, most responders (51%) do so before commencing a study, although 37% indicated that they sometimes perform this analysis, while 12% do not conduct this analysis (Supporting Information S1, Q34). Most responders (35%) indicated that they used data from their laboratory from similar studies as the basis for their a priori analysis, and a similar proportion (31%) would use data from an equivalent published study (Supporting Information S1, Q35). Approximately 15% would conduct a pilot study for the project from which to calculate their a priori sample size. Almost 20% chose the “Other” category where most indicated that they would choose either of the first three options. For the choice of sample size, the overwhelming majority (79%) of researchers would use a sample size of between 6 and 10 specimens (Supporting Information S1, Q36). Less than 10% would use greater than 10 samples, and 7% would only use 3‐5 samples. The remaining 5% chose “Other” where they indicated that they would conduct an a priori sample size calculation first.

Most researchers (66%) perform tests for normality on their data before selecting either a parametric or nonparametric statistical analysis, with another 25% indicating that they would sometimes perform this test (Supporting Information S1, Q37). Seven percent do not test for normality.

Almost 50% of researchers would use a clinically relevant difference between groups as the basis of their interpretation of statistical findings with a further 37% indicating that they would sometimes do this (Supporting Information S1, Q38). A small proportion (11%) do not compare to clinically relevant differences, and 5% (Other) pointed out that clinically relevant differences are not always available and would use them if they were.

The responses from researchers regarding how they determined the clinically relevant difference varied across the first three options (Supporting Information S1, Q39). The majority (64%) would use in vivo data from either the same or a related treatment, and a further 19% would use in vivo data from an unrelated treatment but the same spine region. Almost 15% chose “Other” were most preferred the same three options and noted that this also depended on the research question.

The majority of respondents (53%) would sometimes treat each spinal level as separate groups in their statistical analysis, and a further 34% said they always would (, Q40). Ten percent would not separate each spinal level, and one in the “Other” category would first test for difference and then pool the levels if no differences were present.

Most researchers (59%) indicated that they had validated their findings by either repeating a study or collaborating with another lab to repeat their study. However, 39% indicated that they have not validated their findings (Supporting Information S1, Q41). One researcher in the “Other” category had not validated their findings due to limited availability of funding, however, they noted the importance of validating when possible.

## 4 CONCLUSIONS FROM LITERATURE REVIEW

The broad range of experimental techniques found in the literature highlights the importance of finding a consensus on factors that can confound mechanical testing data. When designing a research study, it is important to address the specific research question(s) and to justify the chosen methods using evidence from the peer‐reviewed literature. If best practices are beyond the ability of specific labs, it may be more appropriate to seek collaborations with colleagues who have access to best practices or avoid conducting a study that has limited physiological relevance altogether. There are many subtle variables that can significantly confound testing results. Each of these variables should be reported in detail and, if the sample size is sufficient, be included as an independent factor in the data analysis. Ultimately, the goal of experimental design is to reduce the confounding effects of these factors sufficiently so that measured differences in outcomes are due to the treatments and not secondary factors related to variation in techniques. Moving toward a consensus will greatly improve the ability to compare findings across studies and evaluate potential therapeutic strategies.^312^, ^313^

## 5 RECOMMENDED BEST PRACTICES

Decades of experimental spine biomechanics research have enhanced our understanding of the effects that experimental techniques can have on outcomes. This has resulted in a substantial body of work explicitly focused on how to optimize in vitro spine biomechanical testing so that it best predicts in vivo performance of spinal tissue—both native and post‐intervention. Despite the wealth of information on technique, it is impossible to define a universal template for in vitro testing methods because the goals of spine biomechanics studies vary widely. The specific methods most appropriate for a study largely depend on the specific scientific questions of that study. However, the following list of best practices was developed based on the scientific rationale summarized from the available literature for mechanical testing of motion segments.

### 5.1 Sample selection

- The most appropriate specimens for any given study are those that mimic the patient population or tissue properties that are most relevant to the hypothesis being tested.
- Reporting of individual specimen demographics is critical.
- Specimen characteristics, including age, extent of degeneration, sex, spinal level, and bone quality should be determined and reported.
- Studies should be adequately powered to include these characteristics as independent factors in statistical analyses. Alternatively, variations in these parameters should be controlled by either screening or distributing them evenly among treatment groups as much as possible to minimize their potential confounding effects.

### 5.2 Sample preparation

- Formalin‐fixed tissue and its alternatives should not be used for biomechanical testing.
- If specimens are not used immediately after harvesting, they should be wrapped in saline‐soaked gauze, sealed in double plastic bags, and frozen at −20°C or colder until the time of use.
- Specimens should not be subjected to more than 4 freeze‐thaw cycles at −20°C.
- Resection or transection of tissue structures should be reported.
- A high modulus material should be used for specimen potting and care should be taken to eliminate any relative motion between the specimen and the testing apparatus.
- Disc dimensions (eg, height) should be measured and reported as well as whether they were measured before or after mechanical testing. The manner by which dimensions were measured (eg, calipers, CT, MRI) should also be reported.

### 5.3 Testing environment

- Testing duration, temperature, and specimen hydration should be controlled and documented.
- Prior to testing, specimens should be hydrated by constrained immersion in saline under an appropriate preload (detailed below).
- During testing, specimens should be maintained moist at all times either in a 100% humid environment, by wrapping specimens in saline‐soaked gauze or plastic, with periodic irrigation, or with constrained immersion in a bath.

### 5.4 Initial conditions: preconditioning

- Preconditioning protocols should be controlled and reported.
- For ROM testing, two cycles of testing are commonly sufficient to precondition specimens. However, when the intent of preconditioning is to minimize the cycle‐to‐cycle variation in the mechanical response of a specimen, individual specimens should be tested one cycle at a time and the data analyzed after each cycle to determine when preconditioning has been achieved.
- As an alternative to cyclic loading, preconditioning can be achieved by the application of a static load based on the spinal level (Tables 1 and 2).

### 5.5 Initial conditions: preloading

- For ROM testing in load control, during testing, axial compressive preloads should be applied through the axis of rotation of the motion segment (or segments) utilizing the follower load or similar technique.
- The magnitude of axial compressive preload should be dictated by the analogous in vivo intradiscal pressure from which the equivalent axial compressive force can then be calculated using the disc area and an appropriate correction factor (Table 2).^144^

### 5.6 Spine testing strategies

- Unconstrained 6DOF load control, unconstrained 6DOF hybrid position‐load control, or application of unconstrained pure moments with axial compressive preloads are acceptable techniques for ROM testing.
- In the lumbar spine, maximum bending moments in the range of 7.6‐10 Nm are most representative of physiological loading.
- In the cervical spine, maximum bending moments of 2.0 Nm reproduce in vivo motion.

### 5.7 Cyclic and viscoelastic testing

- Cyclic loading tests should be performed until changes in displacement plateau reach an equilibrium (eg, the rate of change in displacement is less than some predetermined threshold).
- Due to extended testing times for creep or recovery (ie, greater than 8 hours), experiments need to be performed within a bath; however, care should be taken to ensure that specimens are not overhydrated prior to testing (Section 5.5).
- In long term static (creep) testing, axial compressive loads range between 460 N and 530 N in the lumbar spine to approximate sitting or standing (Table 2).
- Combined loading protocols (eg, compression with bending or rotation) better represent in vivo loading.
- The order of the applied loading should be controlled and reported to minimize fluid‐flow effects.

### 5.8 Study design

- An a priori sample size calculation, and the rationale for the choice of sample size should be undertaken and justified.
- Repeatability of methodology from prior studies within the same laboratory (or from other external published studies), and/or between other laboratories should be considered.
- Independent validation of laboratory findings to confirm that alternative tests demonstrate general equivalence in results, for example, measurement of disc area compared between using calipers vs X‐ray/CT/MRI, should be performed.
- Normalization of results to an appropriate control, such as a repeated‐measures (paired) study design, if appropriate, should be performed.
- Post hoc power analyses for nonsignificant findings to determine if there truly are no differences between treatment groups, or if the study is merely underpowered, are required.
- Interpretation of results in a clinical context should be presented, including estimations of this with justifications and evidence: how does the experimental effect size relate to the clinically relevant effect size?

## CONFLICT OF INTEREST

The authors have nothing to disclose and have no conflicts of interest.

## Supporting information


**Appendix S1**: Final questionnaire responses

**Appendix S2**: List of survey responders
