# Supplementary material for: Spine biomechanical testing methodologies: The controversy of consensus vs scientific evidence
Source: JOR Spine. 2021 Jan 5;4(1):e1138. doi: 10.1002/jsp2.1138 (PMC7984003; doi:10.1002/jsp2.1138)
Supplement: Supplementary file 1 — Appendix S1: Final questionnaire responses [file JSP2-4-e1138-s002.pdf]

Supplemental Material S1: Google Survey Responses

Dear Colleagues,

We would like to invite you to complete the following survey that will be used to determine the current consensus on spine biomechanical testing across the international spine research community. Your responses will be summarized in an issue of JOR Spine and we hope that you are able to contribute your valuable expertise to this important consensus paper. The survey is anonymous, however, if you would like your name and affiliation listed as a contributor to the survey then you can include your details after the final question.

Thank you for your important contribution towards improving the quality of scientific research on spine segment biomechanics.

Kind regards,

John Costi
Grace O'Connell
Eric Ledet

Survey Questions - Please skip any questions that are not relevant to you, or for which you have no opinion, or for which you have no experience. All questions are to be answered with respect to testing of HUMAN CADAVER motion segments unless specified otherwise. If you respond "Other", a comment box is provided. This survey will take approximately 15 minutes to complete.

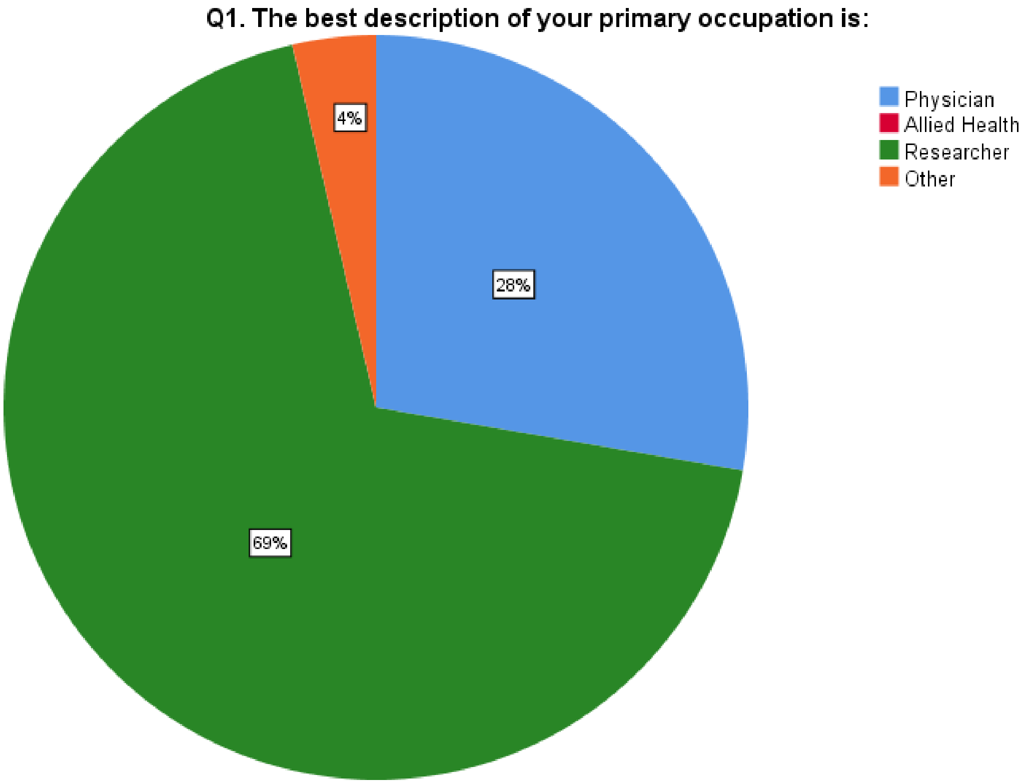

Q2. [Physicians Only] How often do you refer to basic science studies on spine biomechanics to guide your decisions in clinical practice?

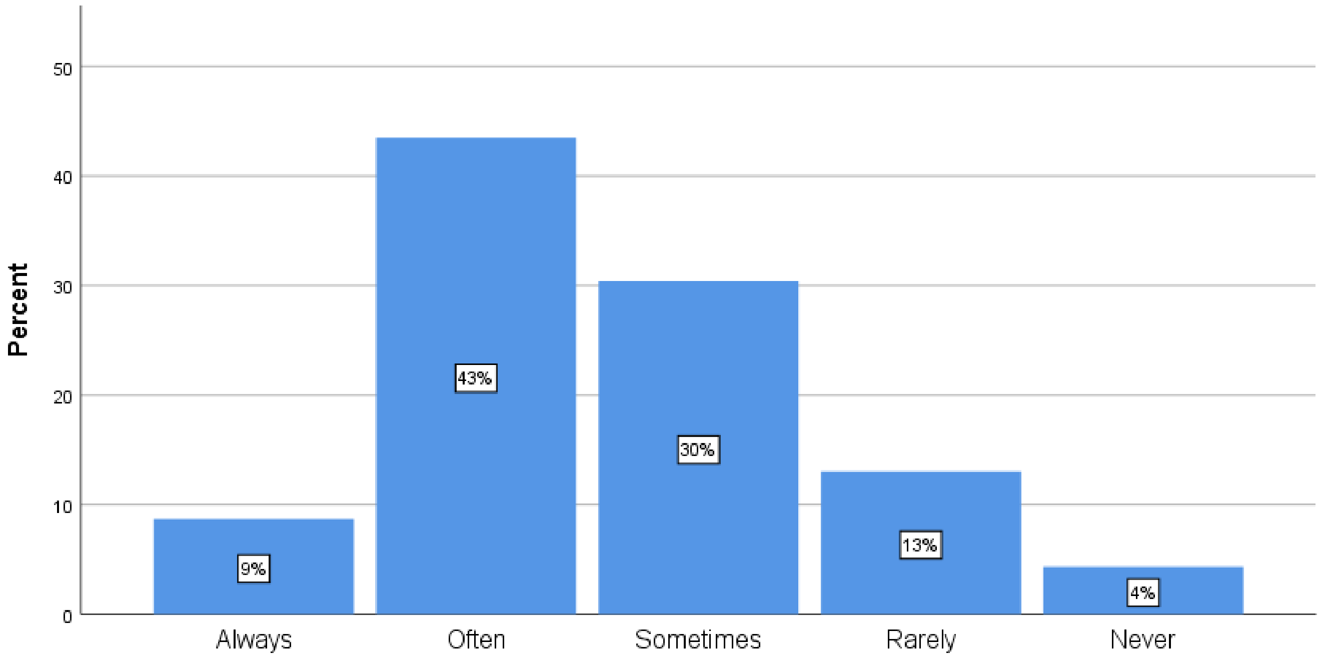

Q3 [Clinical Relevance] [Physicians only] Which types of studies generally provide the highest value in predicting clinical performance of a new spinal therapy, implant, or technique? (23 responses)

1. Computer simulations Including Finite Element Analysis
2. In Vivo Small Animal Studies
3. In Vivo Large Animal Studies
4. In Vitro (Cadaver) Studies
5. In Vitro (Cell) Studies
6. Bench Mechanical Testing (Non-Tissue Based, i.e. ASTM)

Which types of studies generally provide the highest value in predicting clinical performance of a new spinal therapy, implant, or technique? (Choose one for each.)

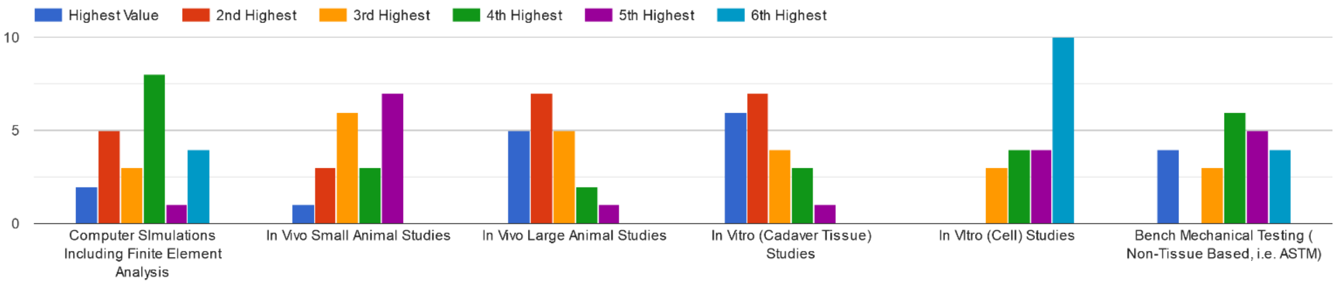

Q4. [Physicians Only] With respect to clinical relevance, what is your comfort level with interpreting biomechanical studies on motion segments?

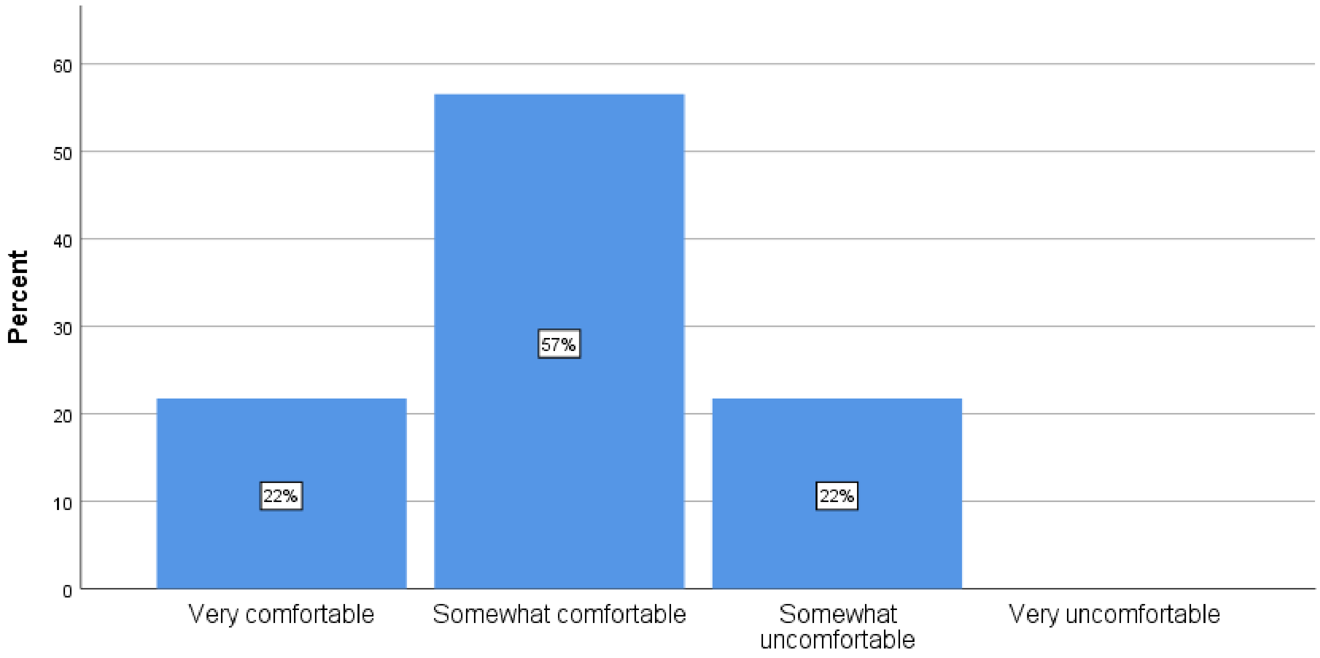

Q5. [Physicians Only] Using non-human specimens for in vitro biomechanical testing (i.e. bovine, porcine, etc.):

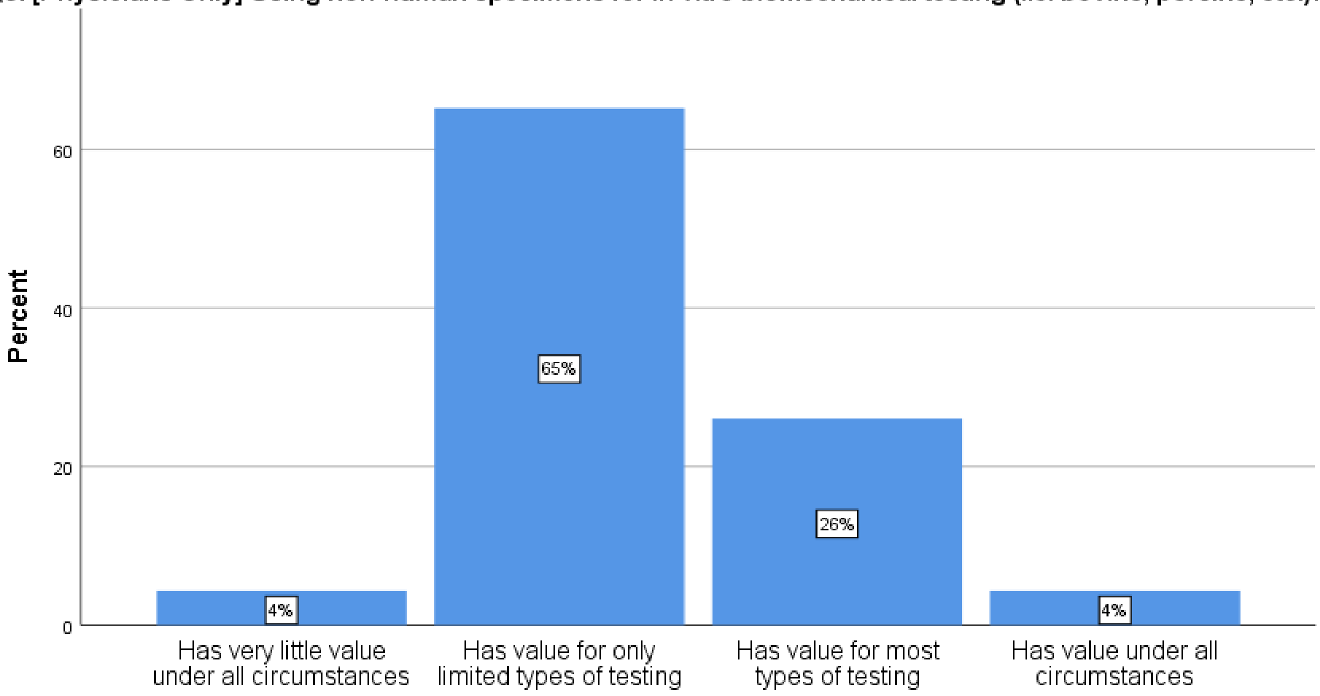

Q6. How many years experience do you have performing in vitro biomechanics research?

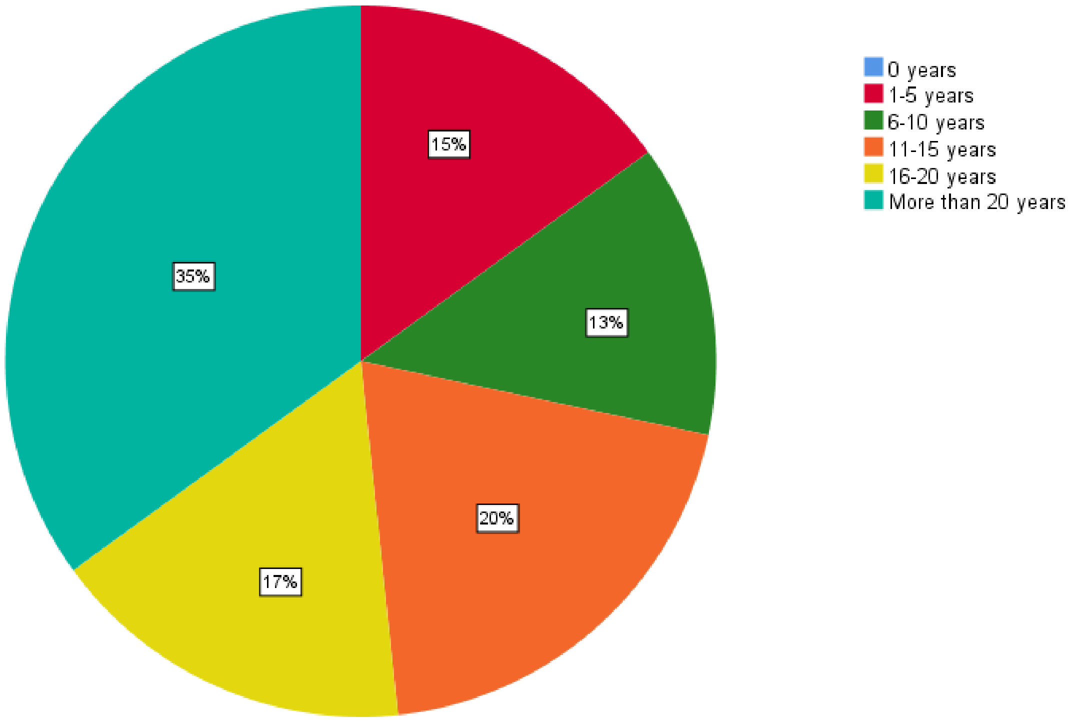

Q6. How many years experience do you have performing in vitro biomechanics research?

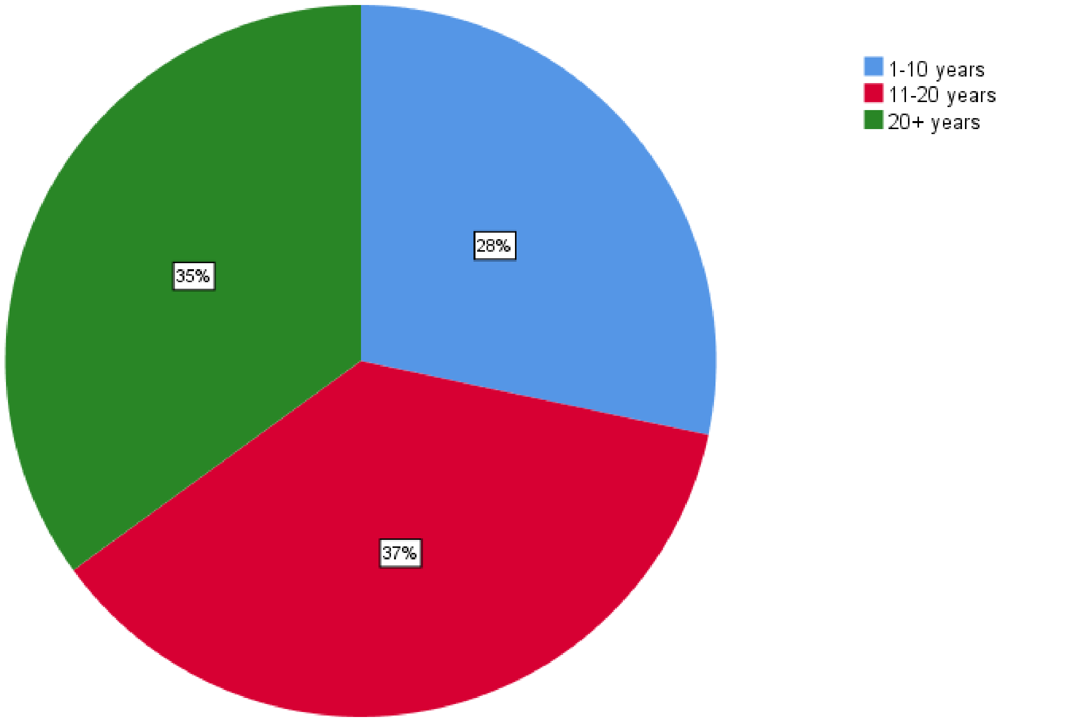

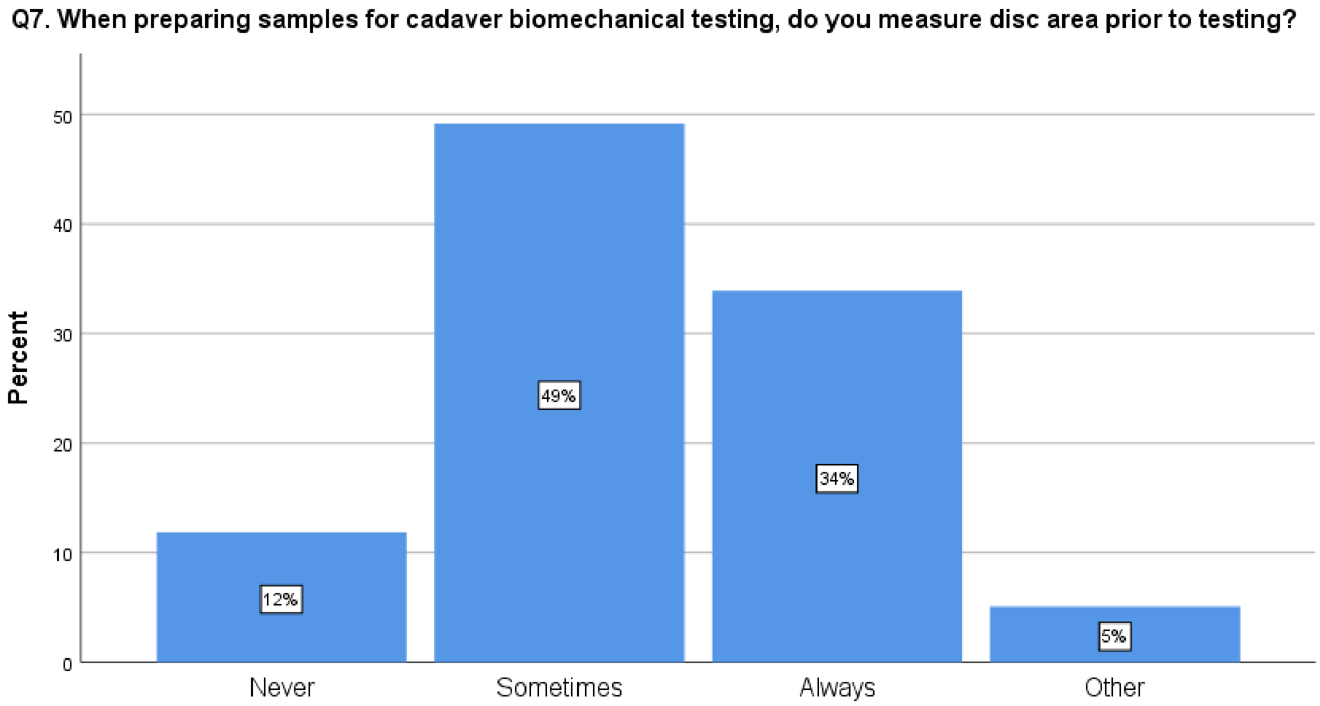
Q7. When preparing samples for cadaver biomechanical testing, do you measure disc area prior to testing?
Percent
0
10
20
30
40
50
12%
49%
34%
5%
Never
Sometimes
Always
Other

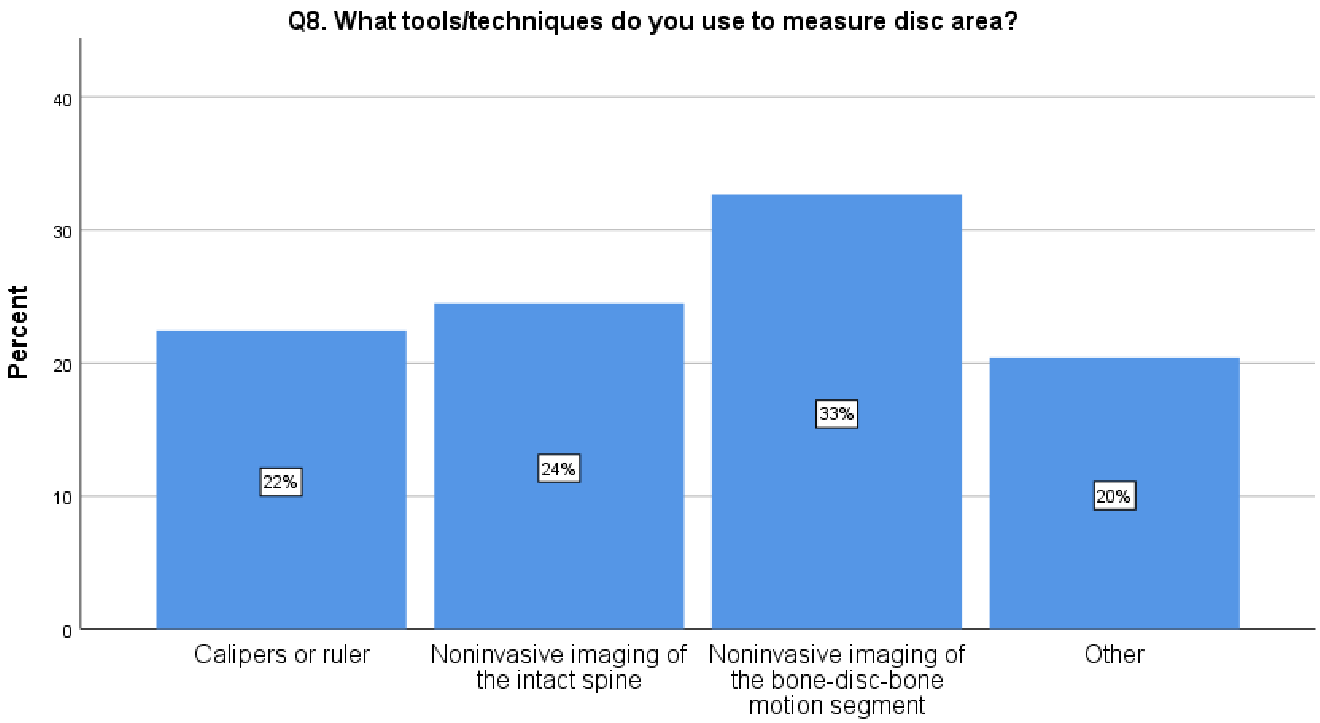
Q8. What tools/techniques do you use to measure disc area?
Percent
0
10
20
30
40
22%
24%
33%
20%
Calipers or ruler
Noninvasive imaging of the intact spine
Noninvasive imaging of the bone-disc-bone motion segment
Other

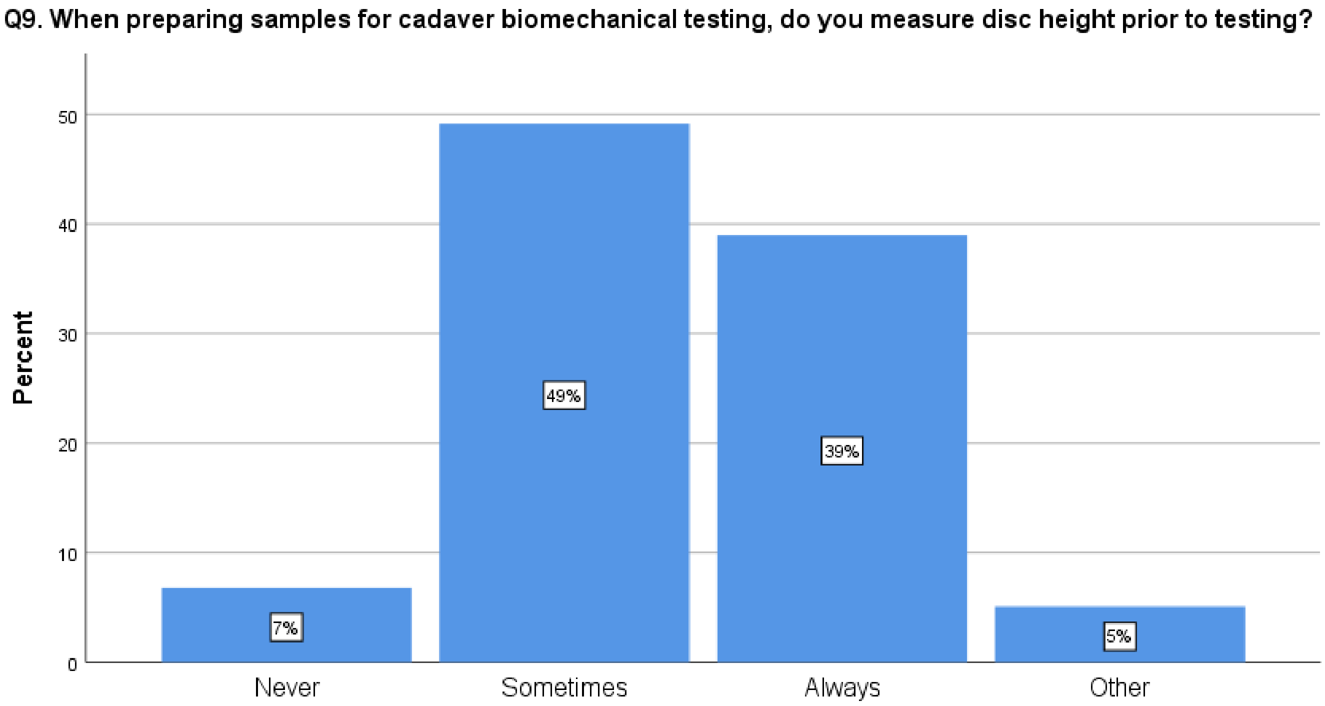
Q9. When preparing samples for cadaver biomechanical testing, do you measure disc height prior to testing?
Percent
0
10
20
30
40
50
7%
49%
39%
5%
Never
Sometimes
Always
Other

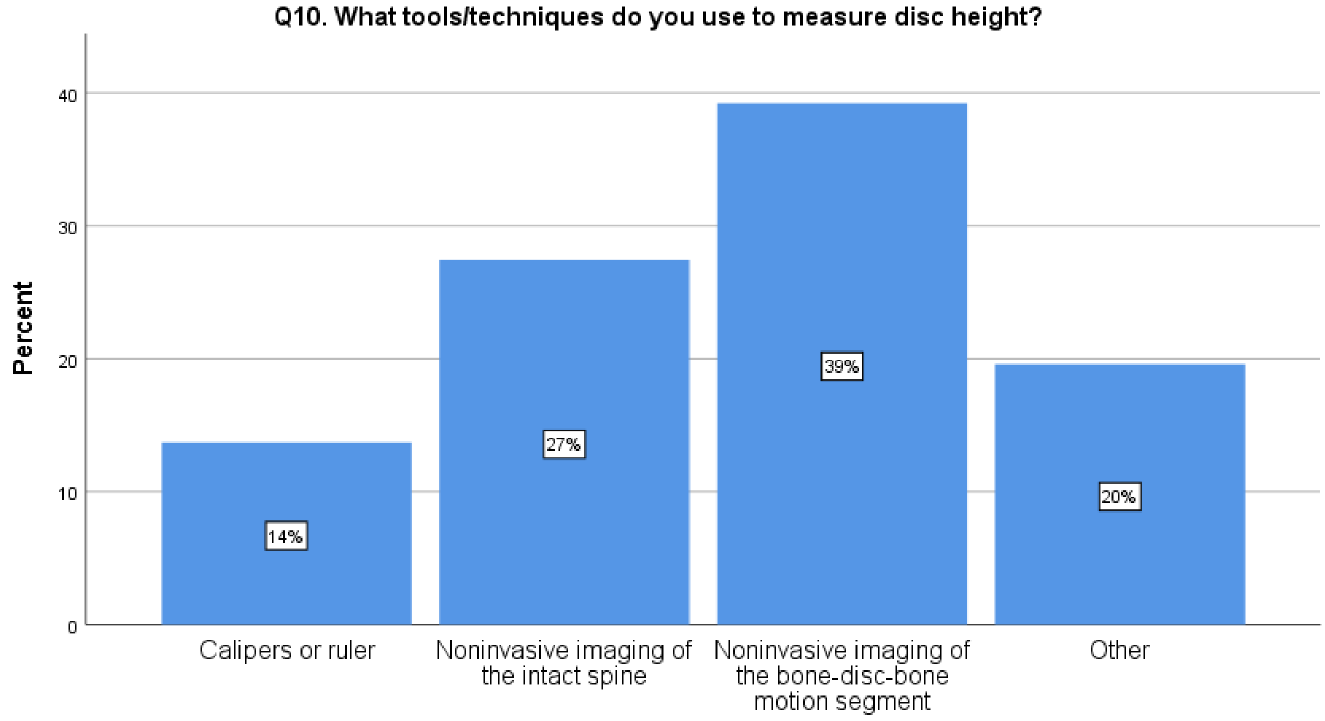
Q10. What tools/techniques do you use to measure disc height?
Percent
0
10
20
30
40
14%
27%
39%
20%
Calipers or ruler
Noninvasive imaging of the intact spine
Noninvasive imaging of the bone-disc-bone motion segment
Other

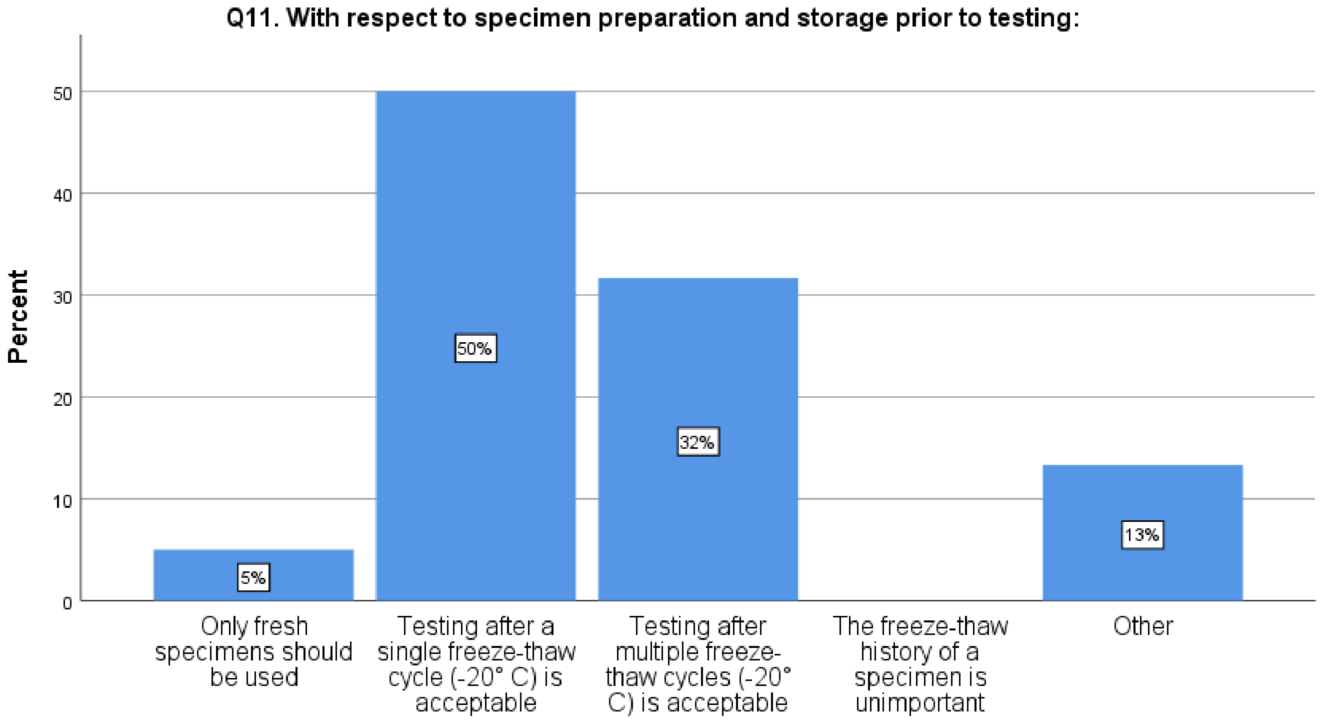
Q11. With respect to specimen preparation and storage prior to testing:
Percent
0
10
20
30
40
50
5%
50%
32%
13%
Only fresh specimens should be used
Testing after a single freeze-thaw cycle (-20° C) is acceptable
Testing after multiple freeze-thaw cycles (-20° C) is acceptable
The freeze-thaw history of a specimen is unimportant
Other

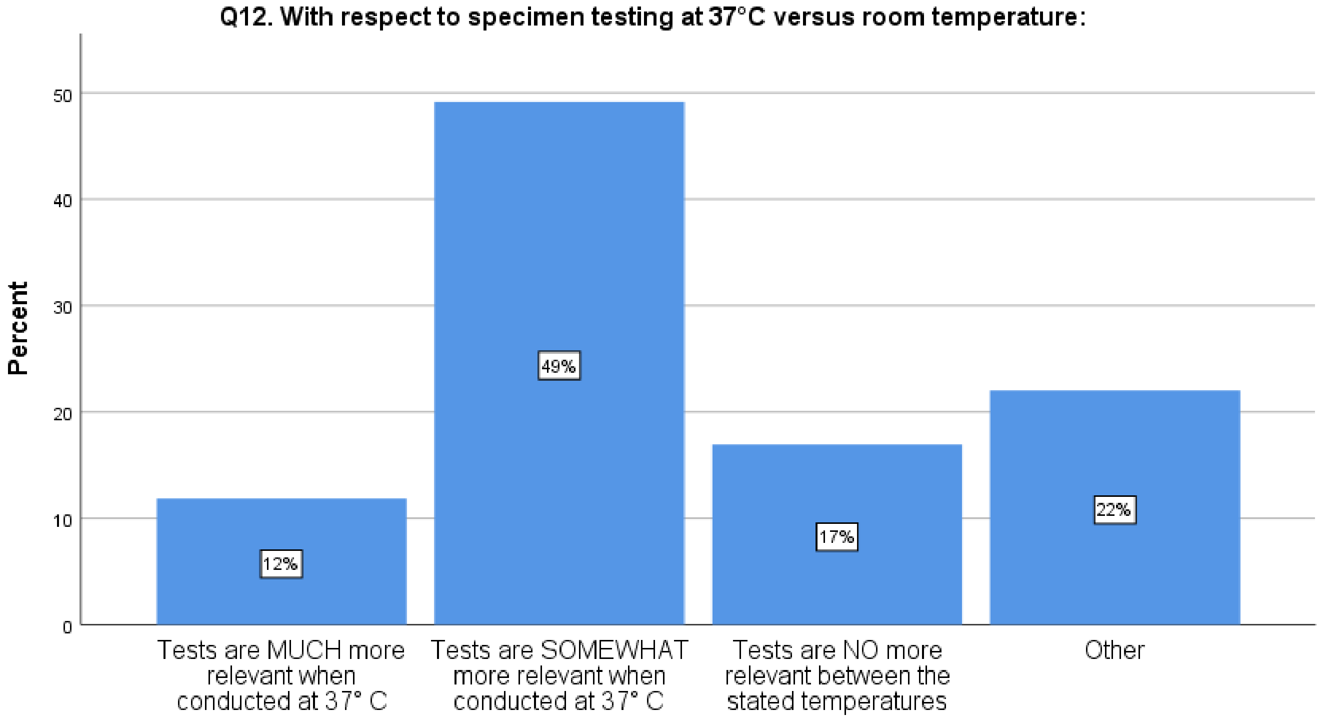
Q12. With respect to specimen testing at 37°C versus room temperature:
Percent
0
10
20
30
40
50
12%
49%
17%
22%
Tests are MUCH more relevant when conducted at 37° C
Tests are SOMEWHAT more relevant when conducted at 37° C
Tests are NO more relevant between the stated temperatures
Other

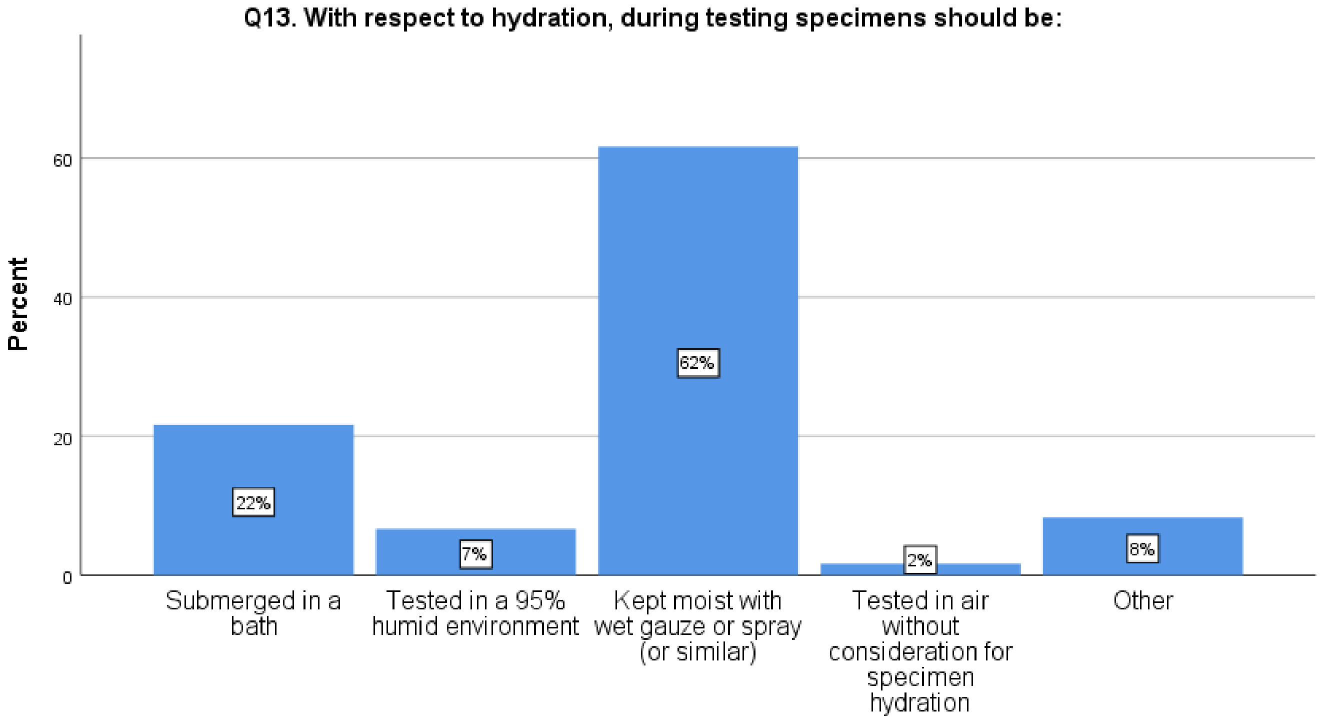
Q13. With respect to hydration, during testing specimens should be:
Percent
0
20
40
60
22%
7%
62%
2%
8%
Submerged in a bath
Tested in a 95% humid environment
Kept moist with wet gauze or spray (or similar)
Tested in air without consideration for specimen hydration
Other

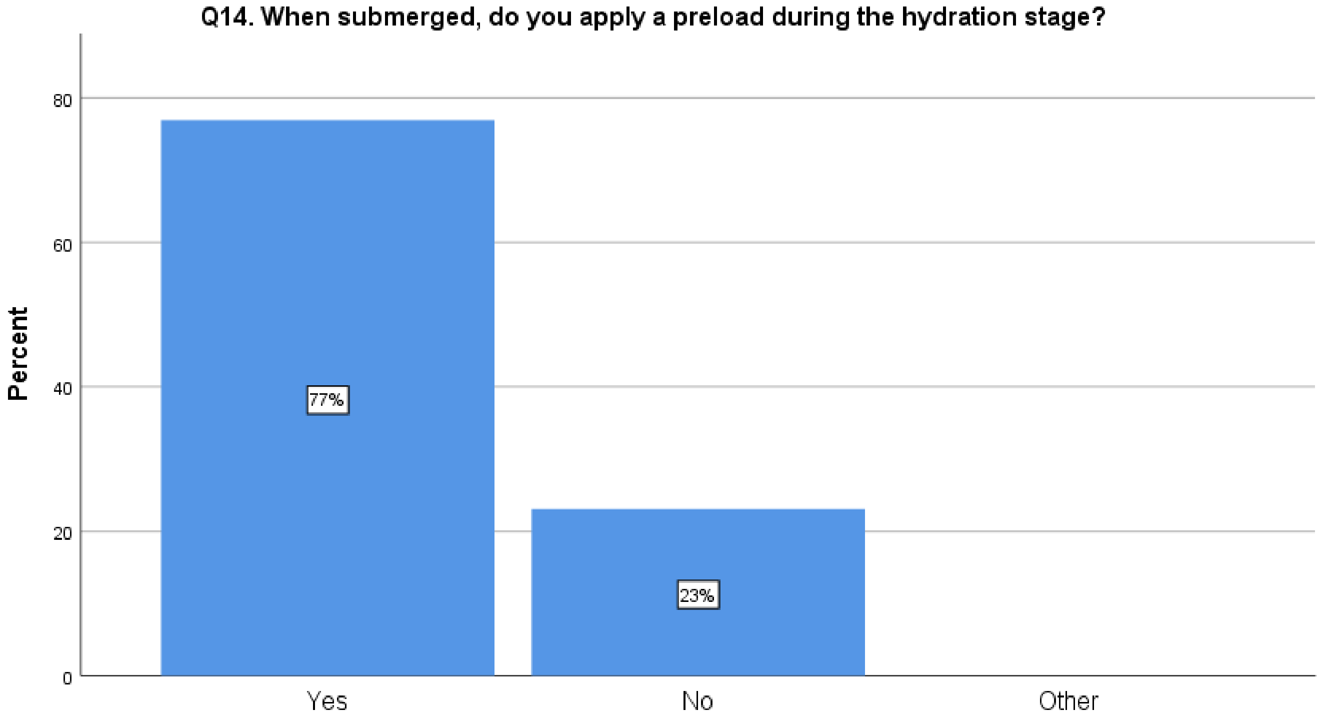
Q14. When submerged, do you apply a preload during the hydration stage?
Percent
0
20
40
60
80
77%
23%
Yes
No
Other

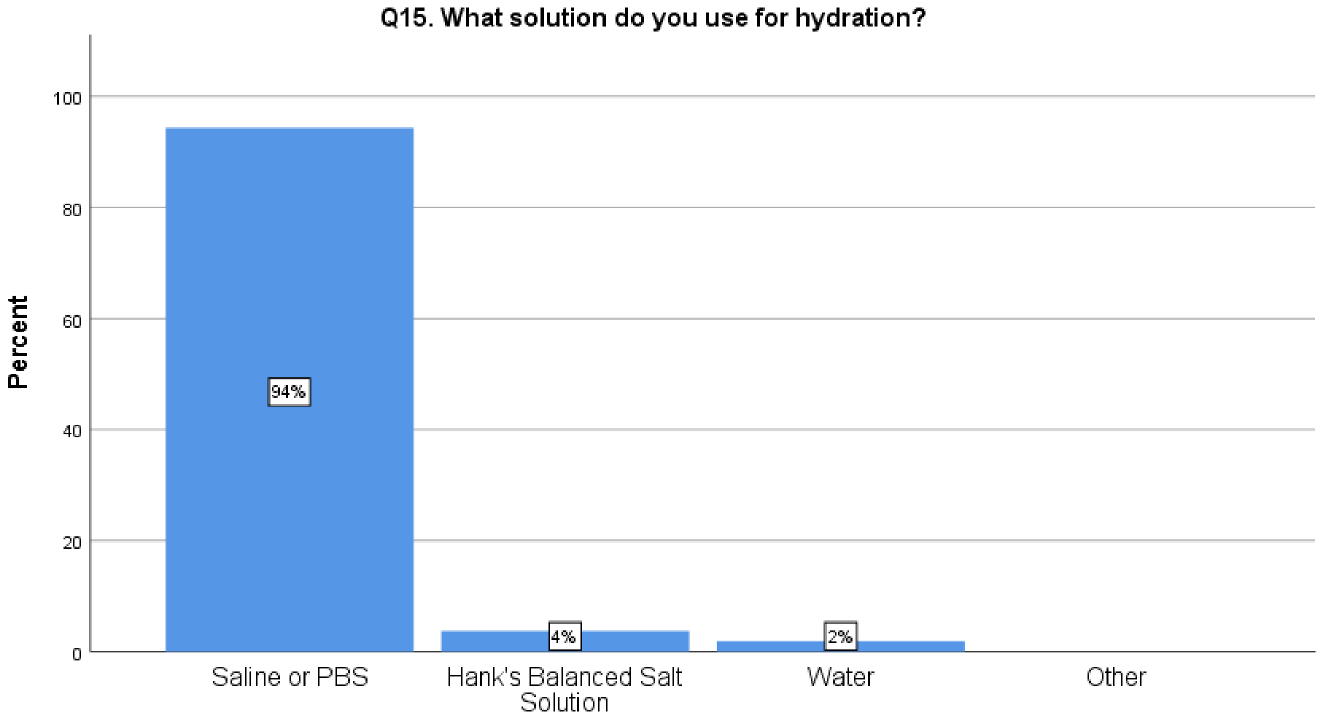

Q15. What solution do you use for hydration?
Percent
100
80
60
40
20
0
94%
4%
2%
Saline or PBS
Hank's Balanced Salt Solution
Water
Other

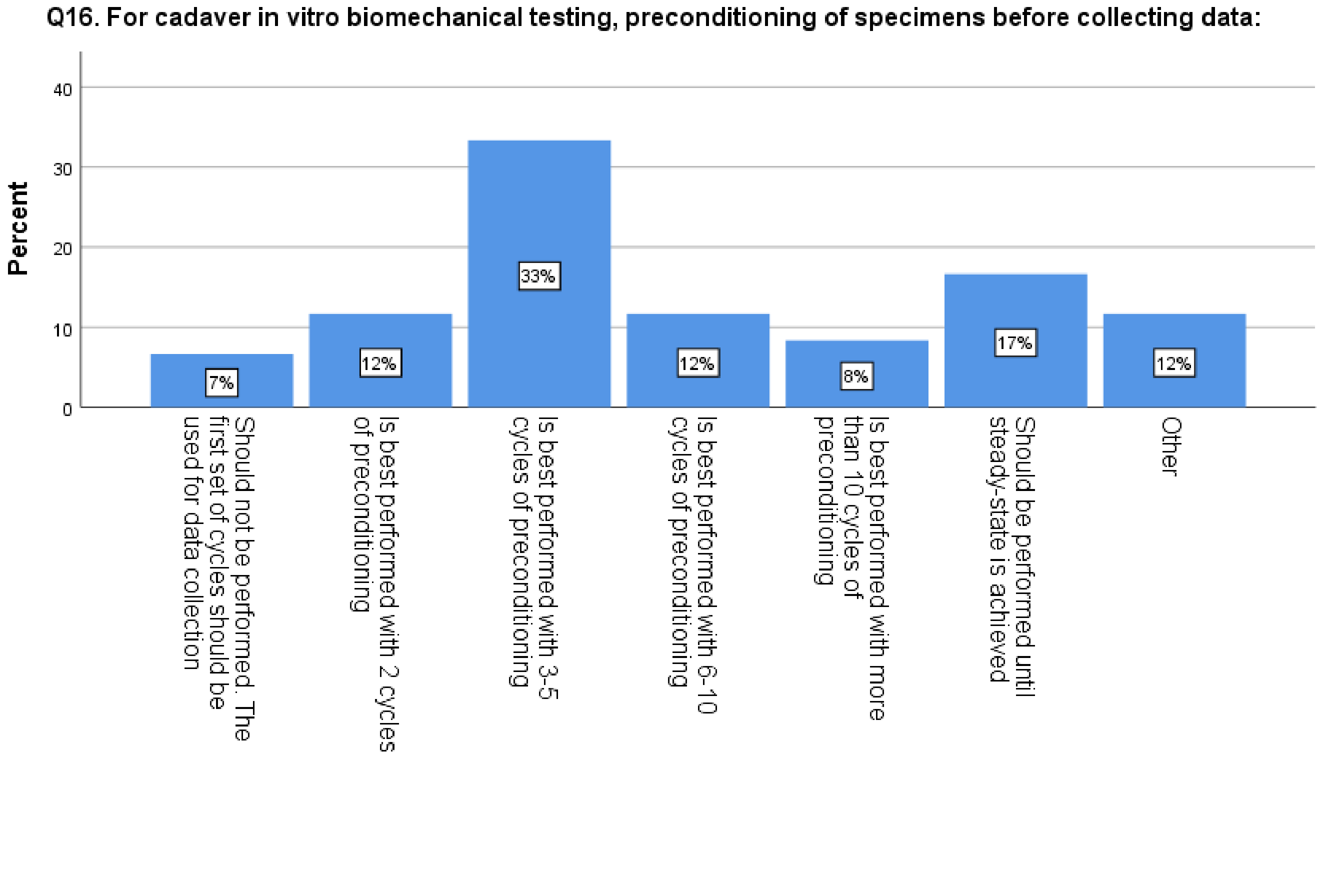

Q16. For cadaver in vitro biomechanical testing, preconditioning of specimens before collecting data:
Percent
40
30
20
10
0
7%
12%
33%
12%
8%
17%
12%
Should not be performed. The first set of cycles should be used for data collection
Is best performed with 2 cycles of preconditioning
Is best performed with 3-5 cycles of preconditioning
Is best performed with 6-10 cycles of preconditioning
Is best performed with more than 10 cycles of preconditioning
Should be performed until steady-state is achieved
Other

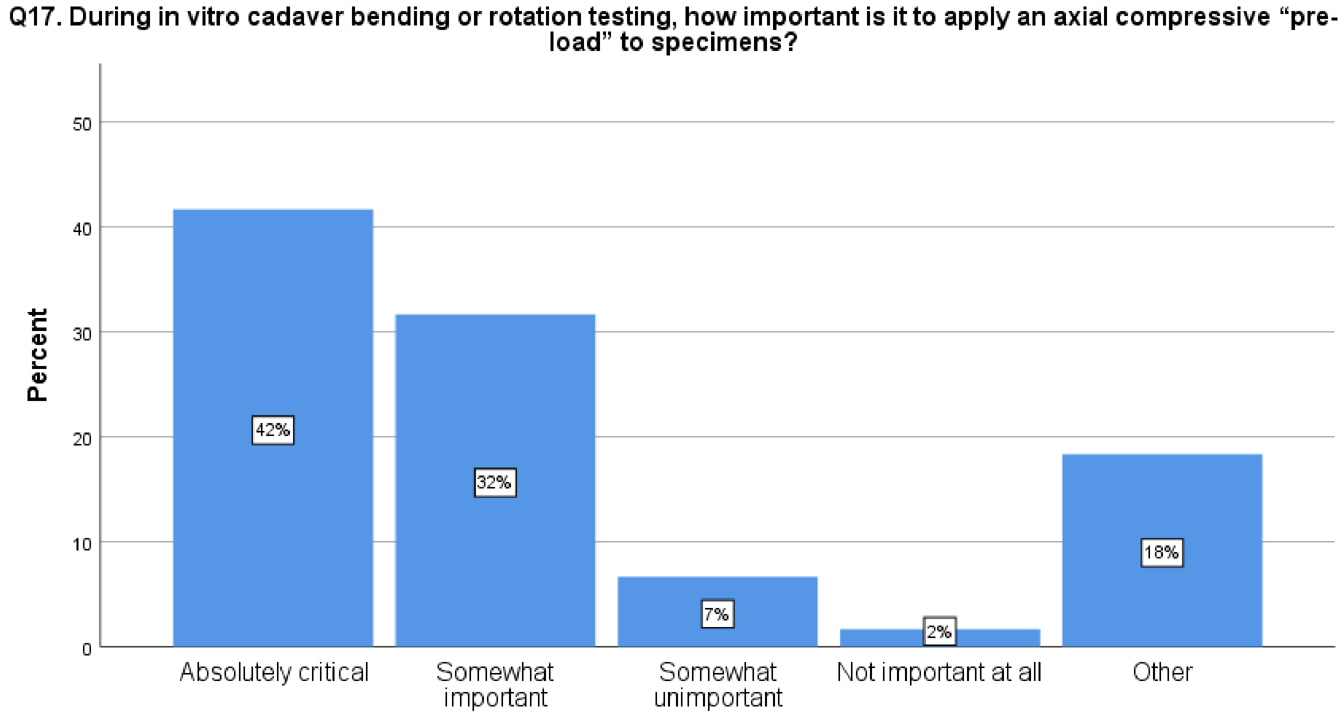
Q17. During in vitro cadaver bending or rotation testing, how important is it to apply an axial compressive "pre-load" to specimens?
Percent
50
40
30
20
10
0
42%
32%
7%
2%
18%
Absolutely critical
Somewhat important
Somewhat unimportant
Not important at all
Other

Q18. For testing LUMBAR spine specimens, what magnitude of axial compressive "pre-load" is most appropriate during bending or rotation testing?

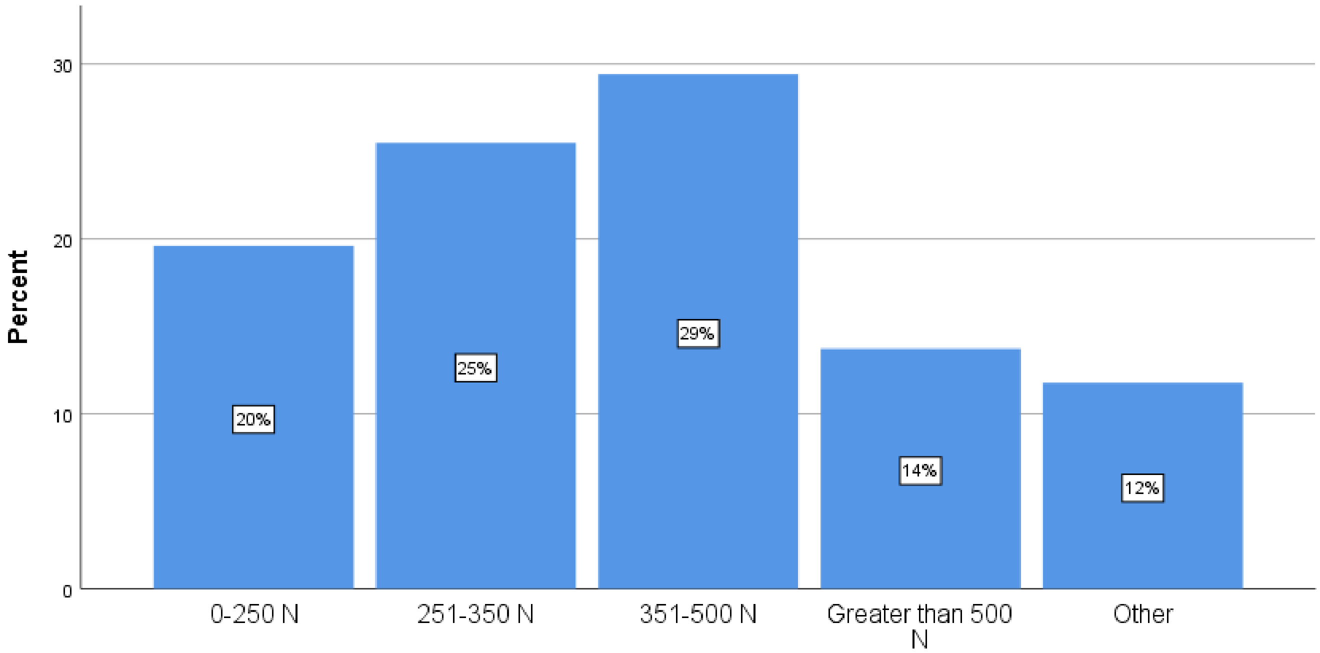

Q19. For testing CERVICAL spine specimens, what magnitude of axial compressive "pre-load" is most appropriate during bending or rotation testing?

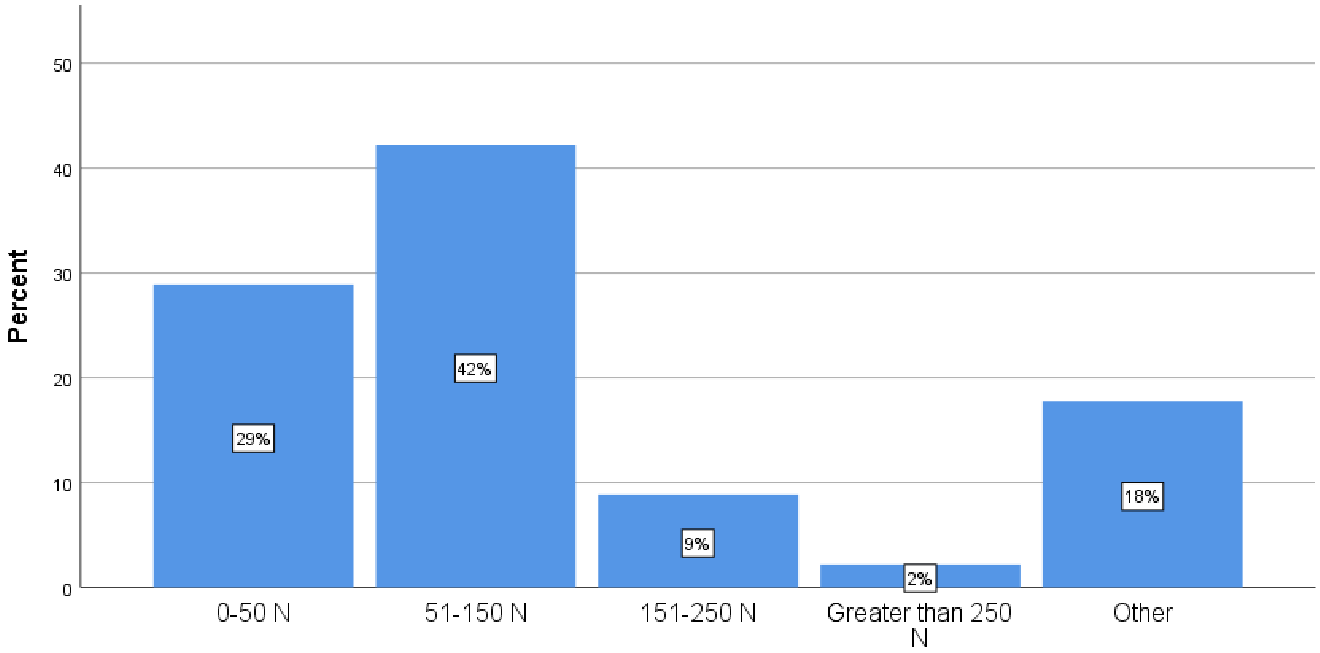

Q20. During in vitro range of motion testing, how important is it to apply pure bending to specimens?

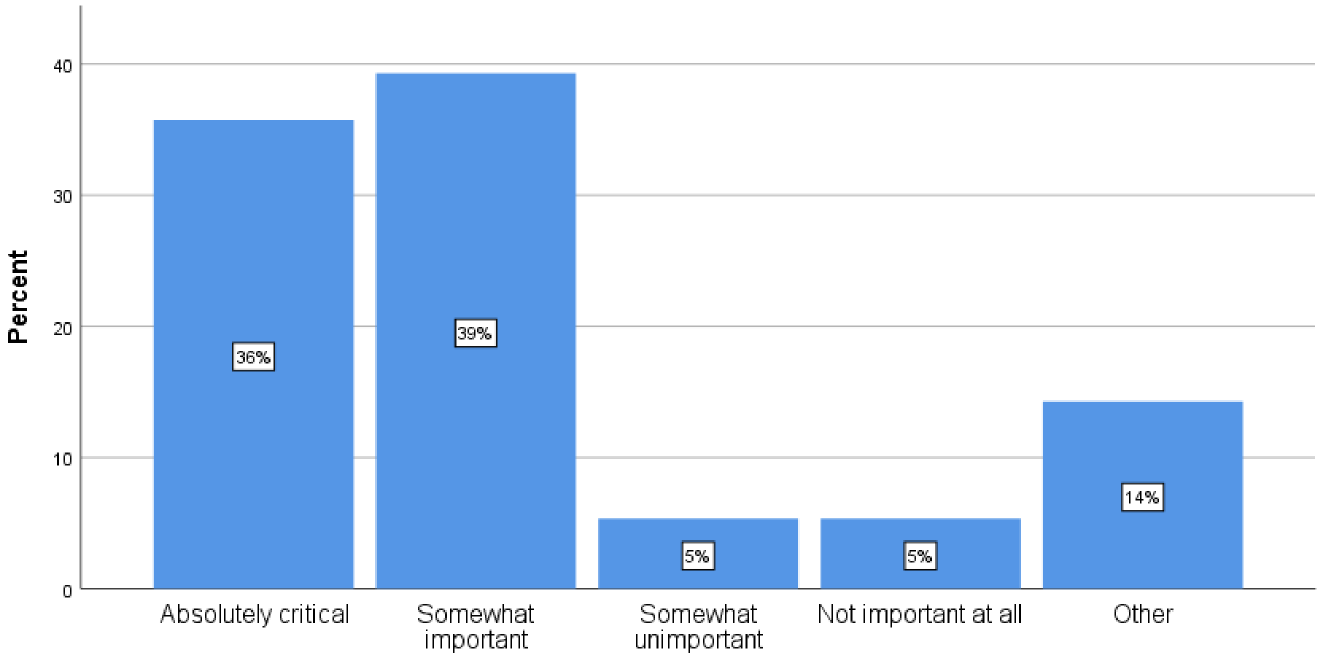

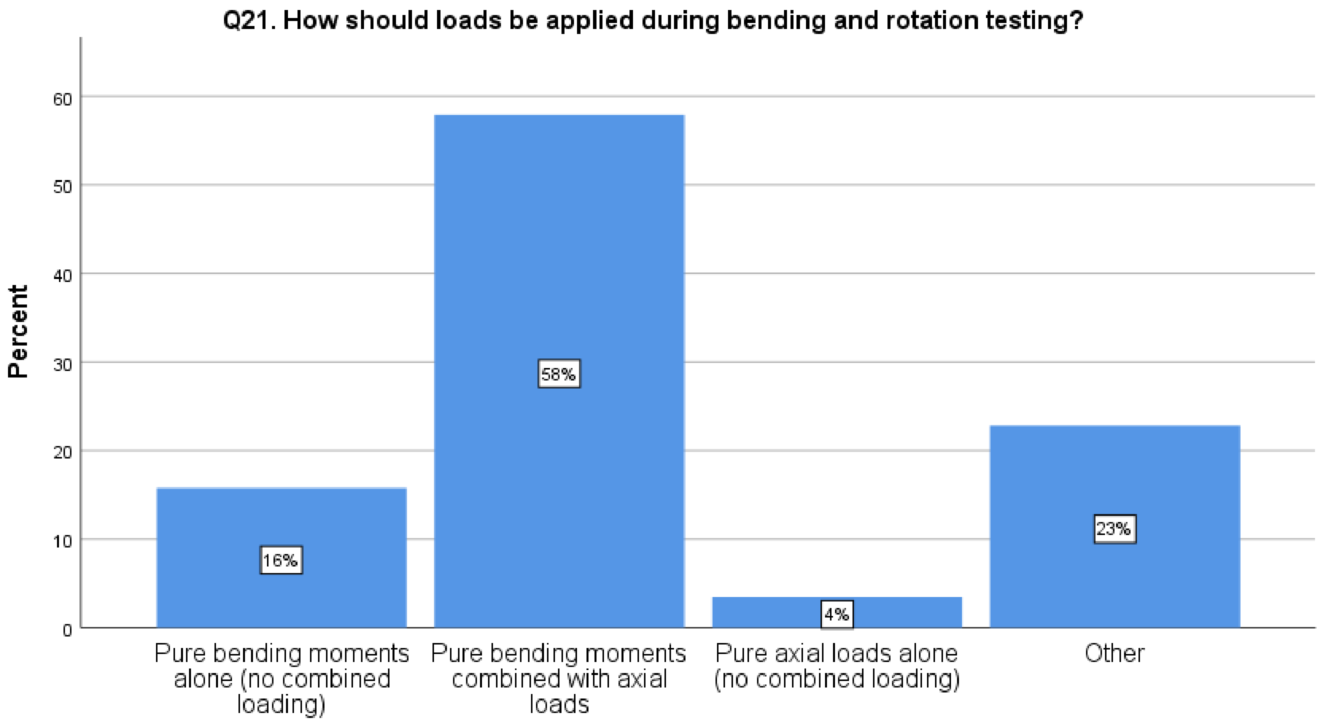
Q21. How should loads be applied during bending and rotation testing?
Percent
0
10
20
30
40
50
60
16%
58%
4%
23%
Pure bending moments alone (no combined loading)
Pure bending moments combined with axial loads
Pure axial loads alone (no combined loading)
Other

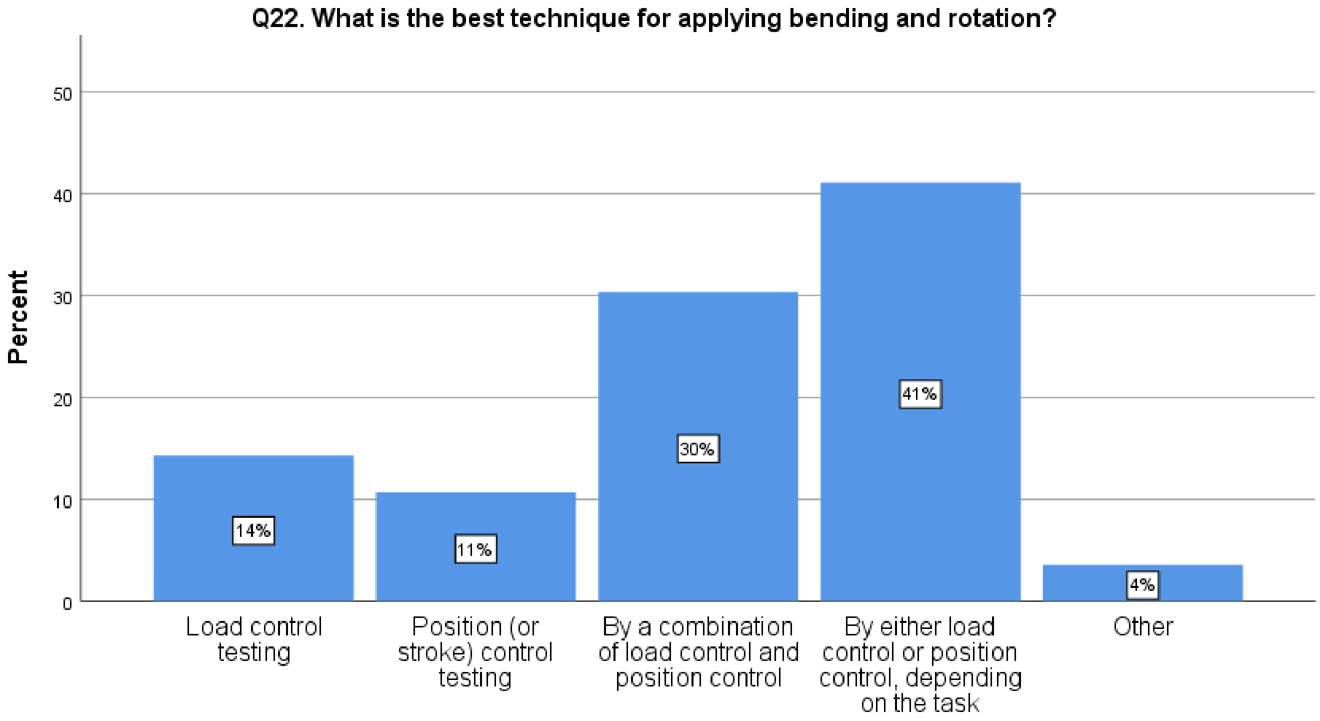
Q22. What is the best technique for applying bending and rotation?
Percent
0
10
20
30
40
50
14%
11%
30%
41%
4%
Load control testing
Position (or stroke) control testing
By a combination of load control and position control
By either load control or position control, depending on the task
Other

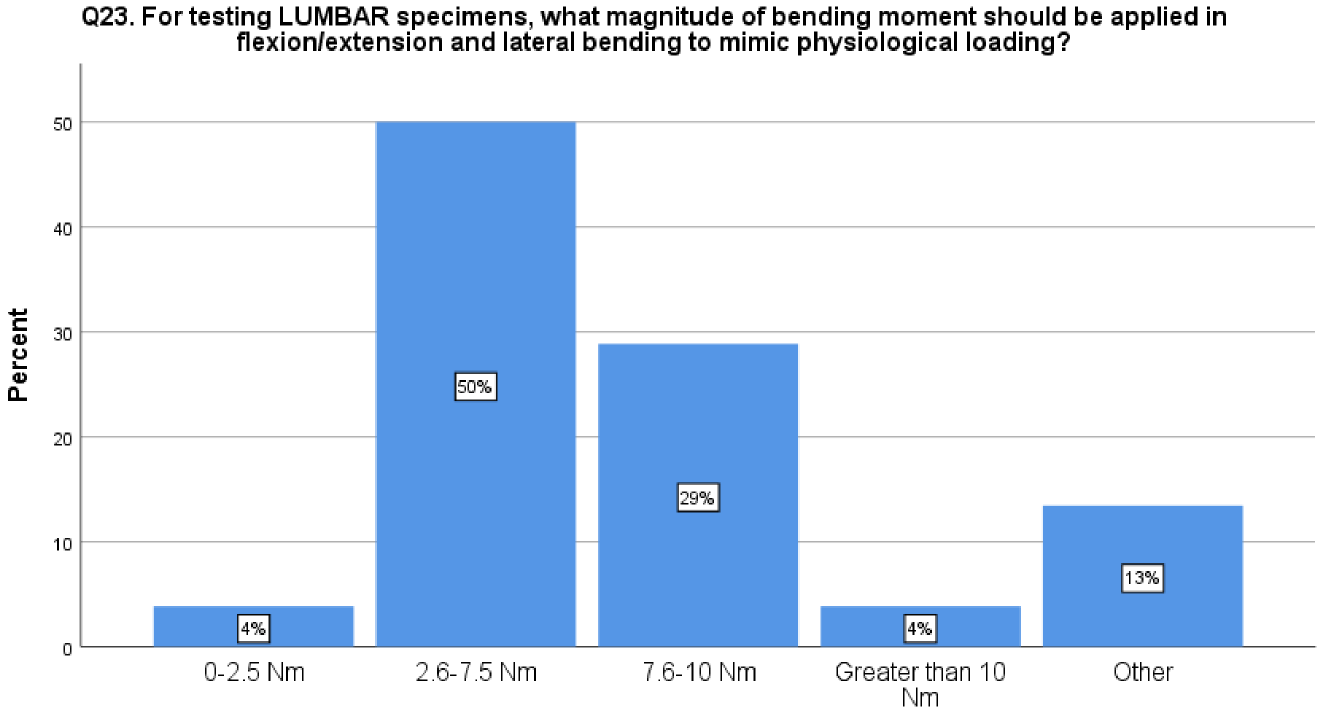
Q23. For testing LUMBAR specimens, what magnitude of bending moment should be applied in flexion/extension and lateral bending to mimic physiological loading?
Percent
0
10
20
30
40
50
4%
50%
29%
4%
13%
0-2.5 Nm
2.6-7.5 Nm
7.6-10 Nm
Greater than 10 Nm
Other

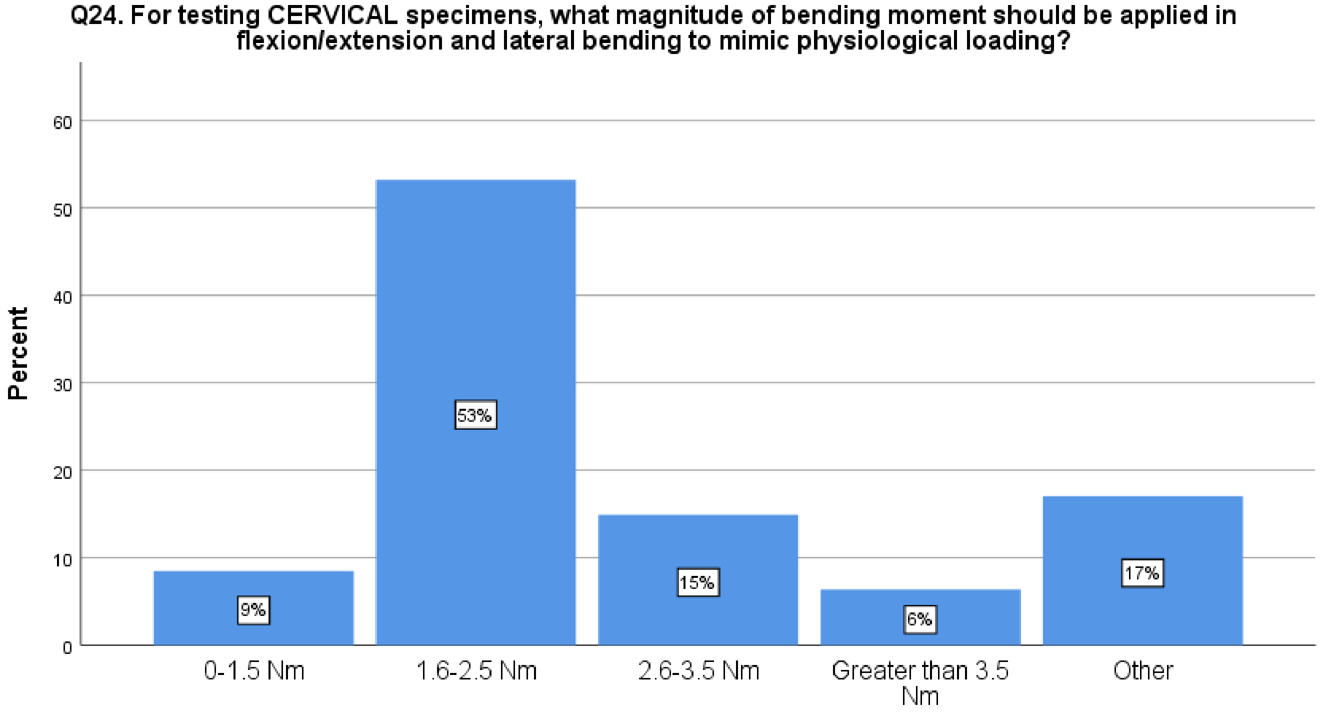
Q24. For testing CERVICAL specimens, what magnitude of bending moment should be applied in flexion/extension and lateral bending to mimic physiological loading?
Percent
0
10
20
30
40
50
60
9%
53%
15%
6%
17%
0-1.5 Nm
1.6-2.5 Nm
2.6-3.5 Nm
Greater than 3.5 Nm
Other

Q25. When testing viscoelastic properties of the disc:

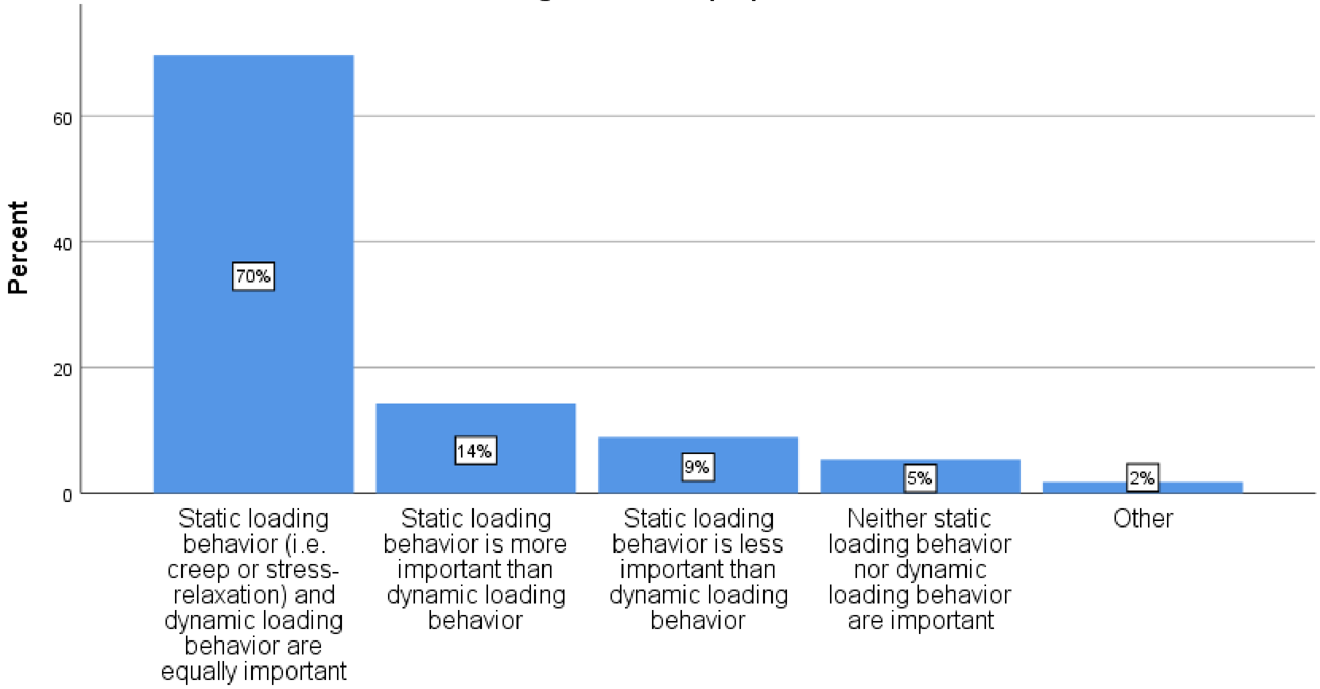

Q26. Do you have experience performing static loading (i.e. creep or stress-relaxation) to study the time-dependent behavior of the disc?

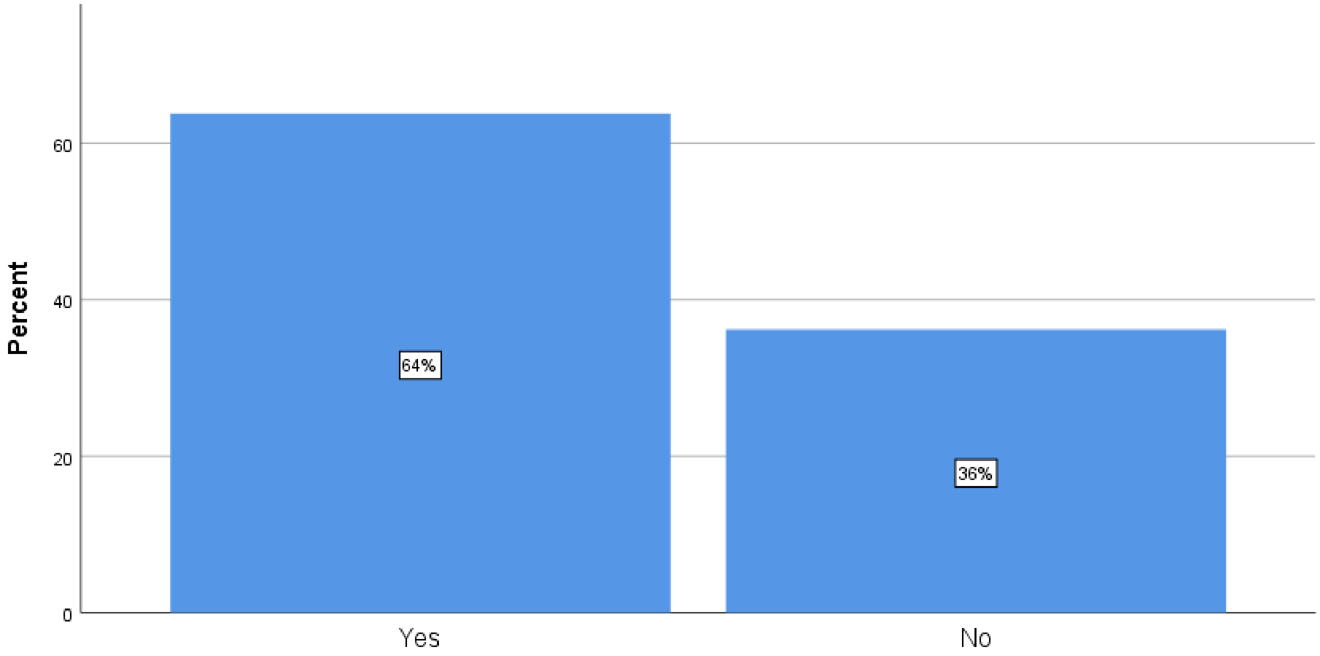

Q27. How long should static loading be applied to study the time-dependent behavior of the disc?

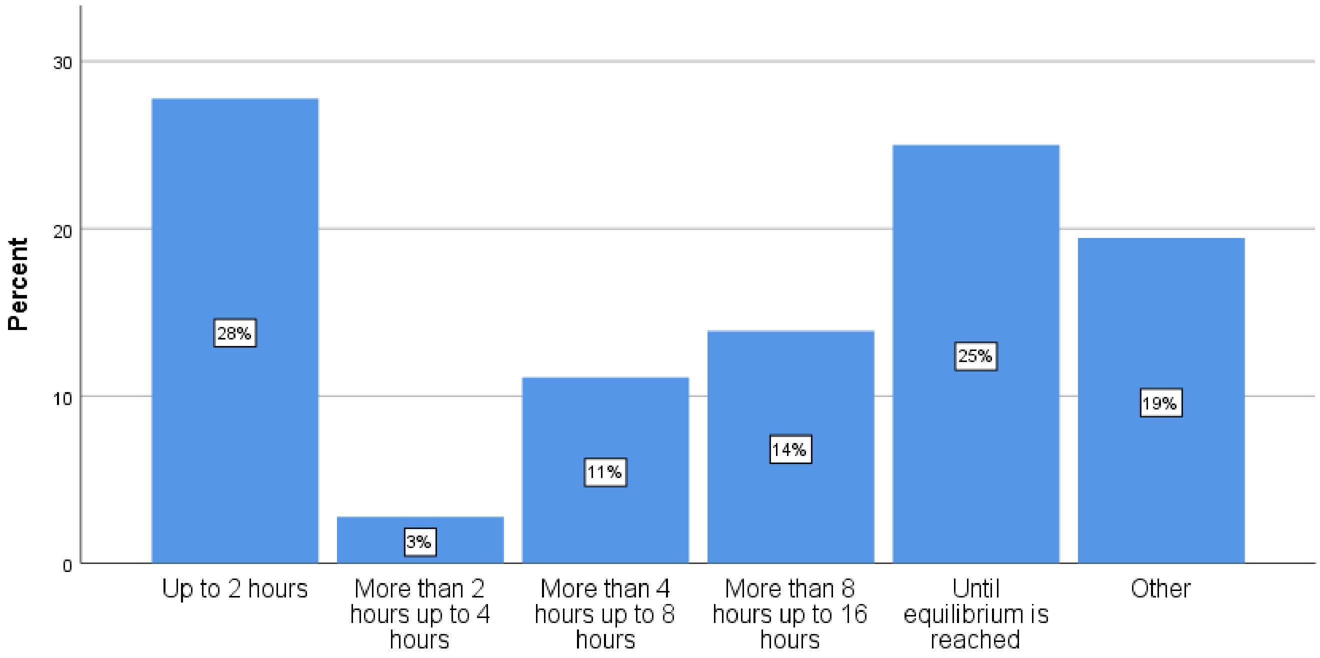

Q28. For reporting the effect that a treatment has on the mechanical properties of a motion segment, it is best:

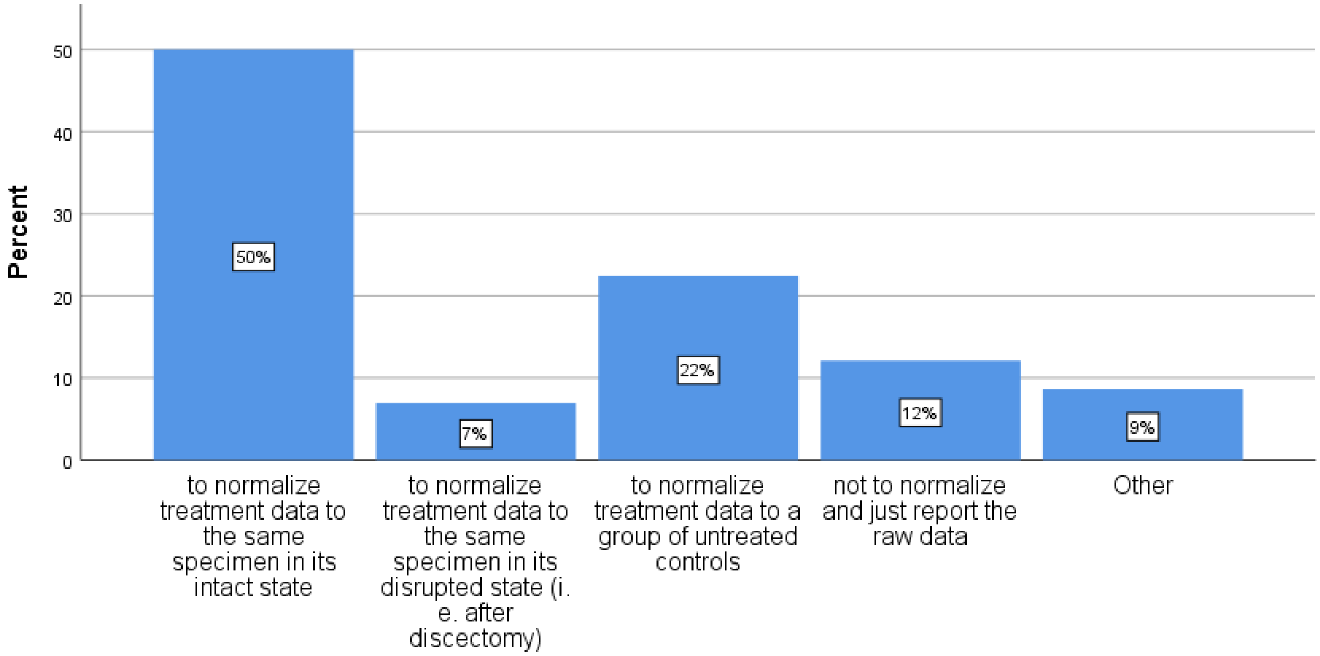

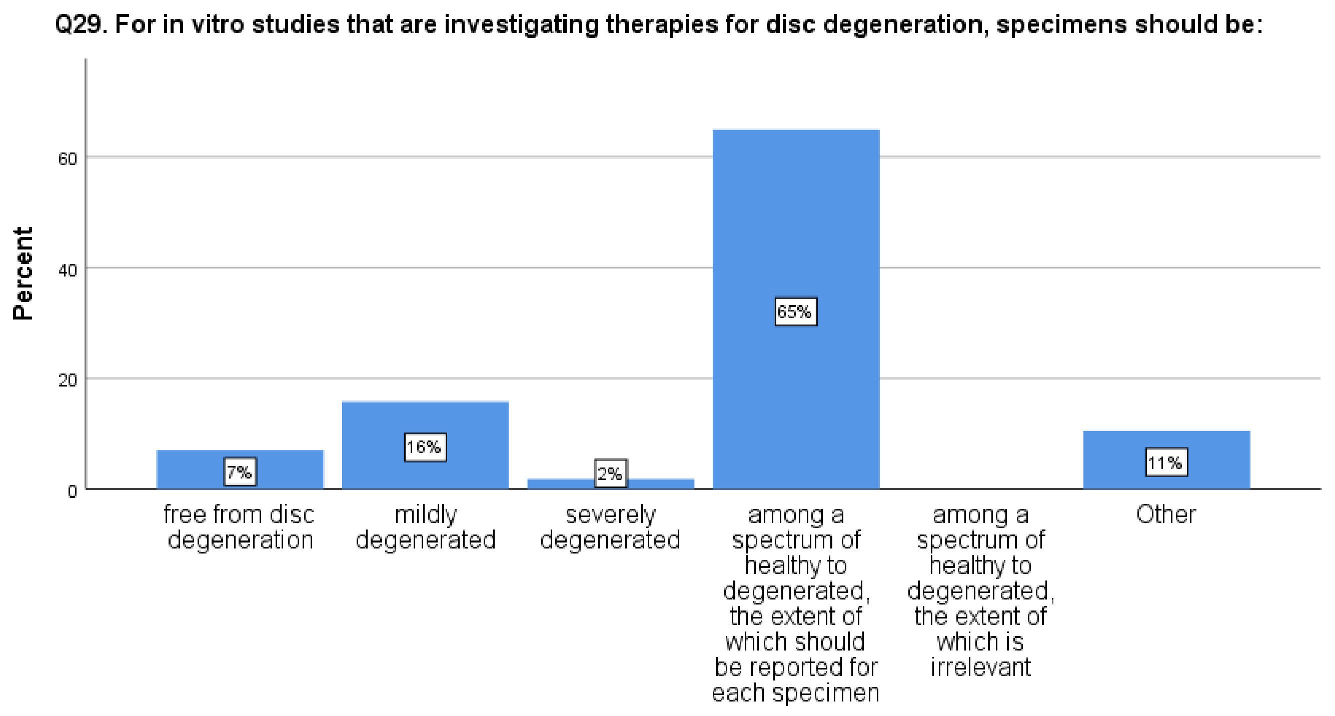

Q30 [Sample Selection & Preparation] For in vitro cadaver spine specimens, the following information should be REPORTED (check all that apply): (60 responses)

1. Donor age
2. Grade/stage of degeneration
3. Disc level (i.e. L45)
4. Disc geometry (height and area).
5. Donor weight or BMI.
6. Bone mineral density.
7. Other

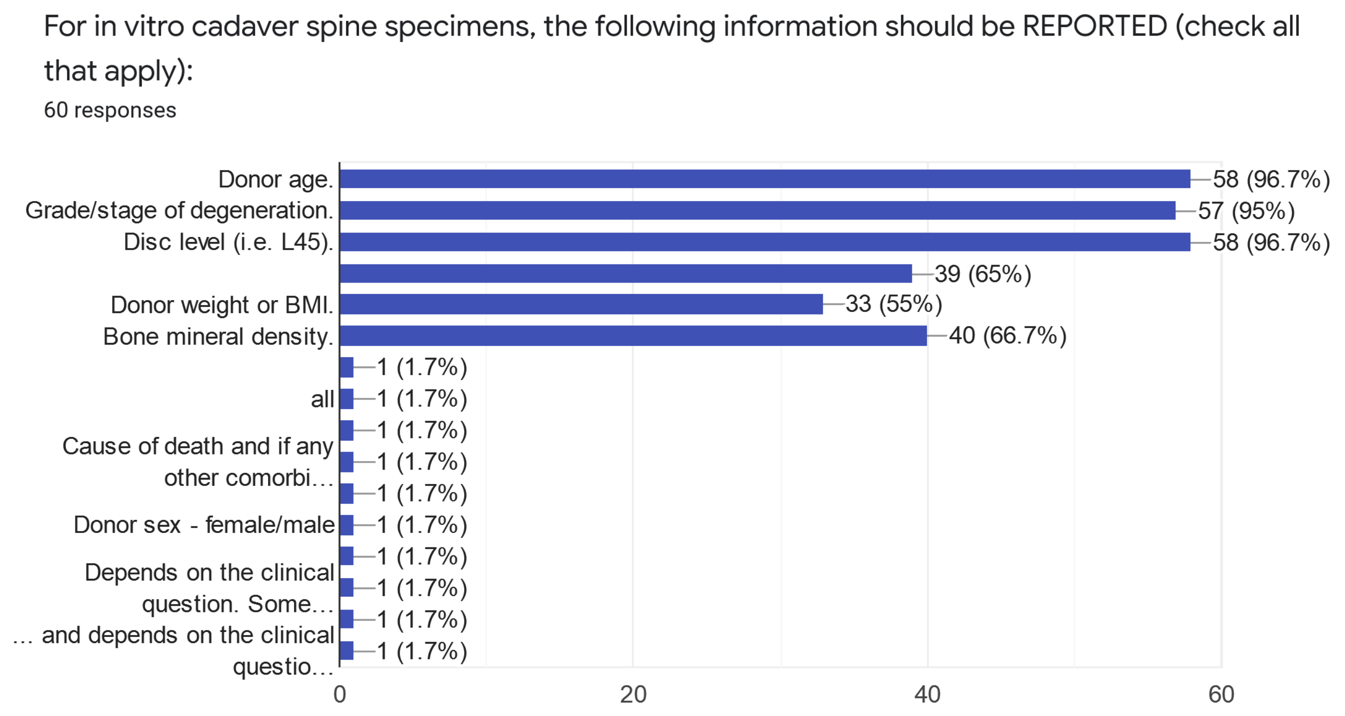

Q31 [Sample Selection & Preparation] For in vitro cadaver spine specimens, the following information should be CONTROLLED (check all that apply): (51 responses)

1. Donor age
2. Grade/stage of degeneration
3. Disc level (i.e. L45)
4. Disc geometry (height and area).
5. Donor weight or BMI.
6. Bone mineral density.
7. Other

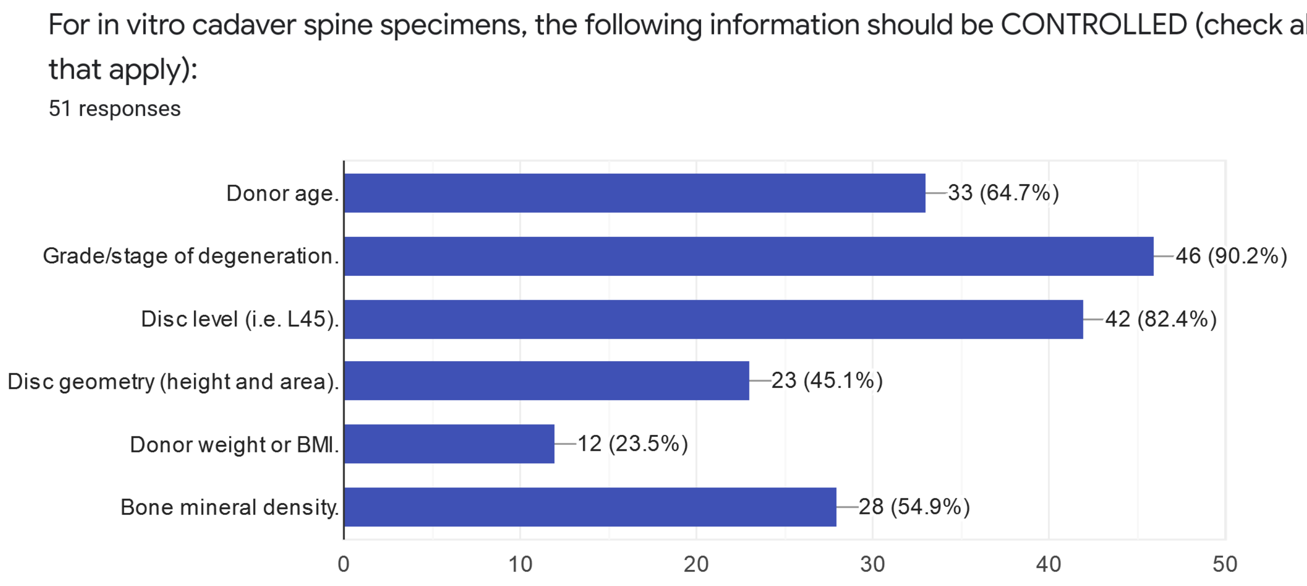

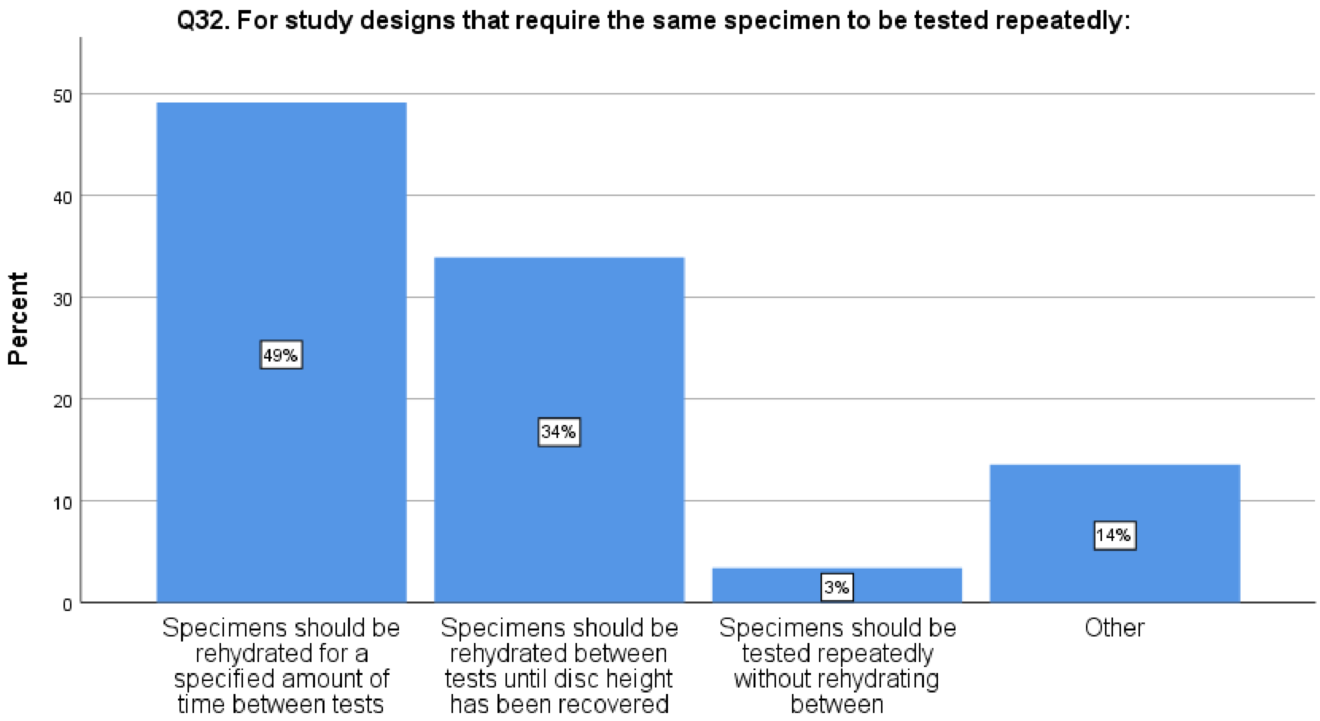
Q32. For study designs that require the same specimen to be tested repeatedly:
Percent
0
10
20
30
40
50
49%
34%
3%
14%
Specimens should be rehydrated for a specified amount of time between tests
Specimens should be rehydrated between tests until disc height has been recovered
Specimens should be tested repeatedly without rehydrating between
Other

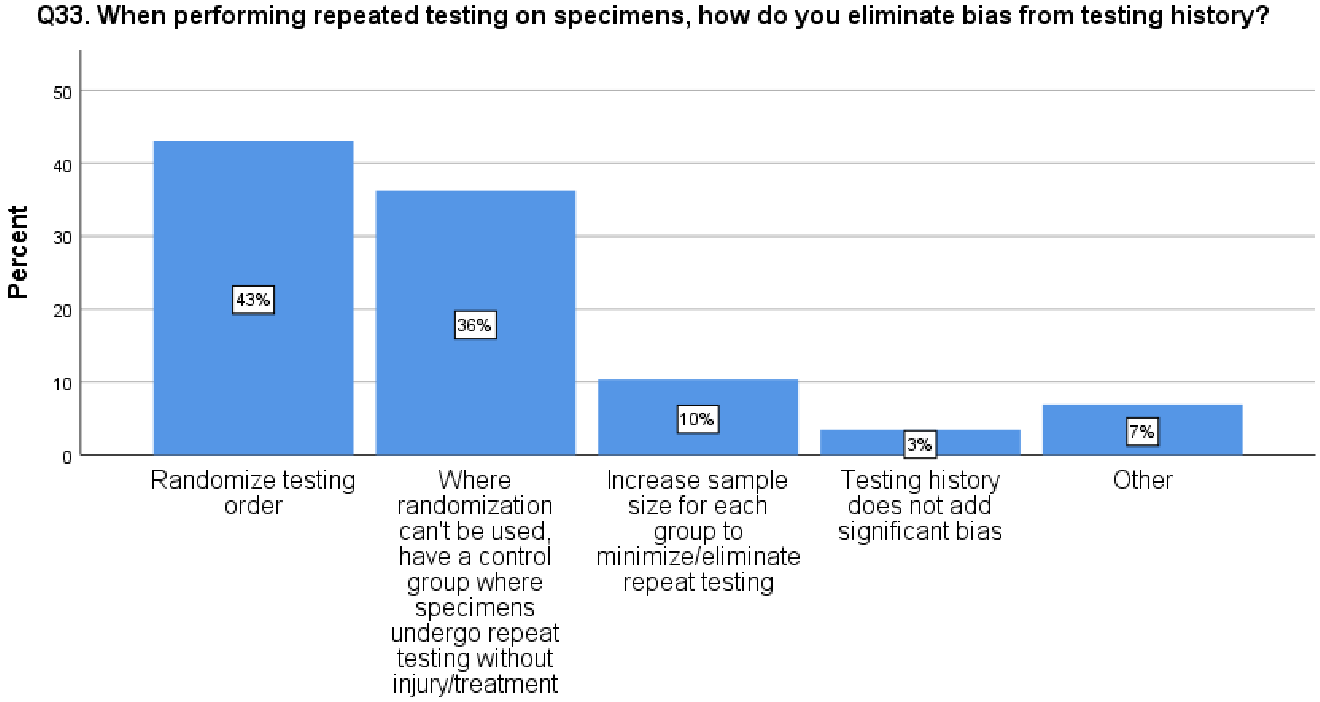
Q33. When performing repeated testing on specimens, how do you eliminate bias from testing history?
Percent
0
10
20
30
40
50
43%
36%
10%
3%
7%
Randomize testing order
Where randomization can't be used, have a control group where specimens undergo repeat testing without injury/treatment
Increase sample size for each group to minimize/eliminate repeat testing
Testing history does not add significant bias
Other

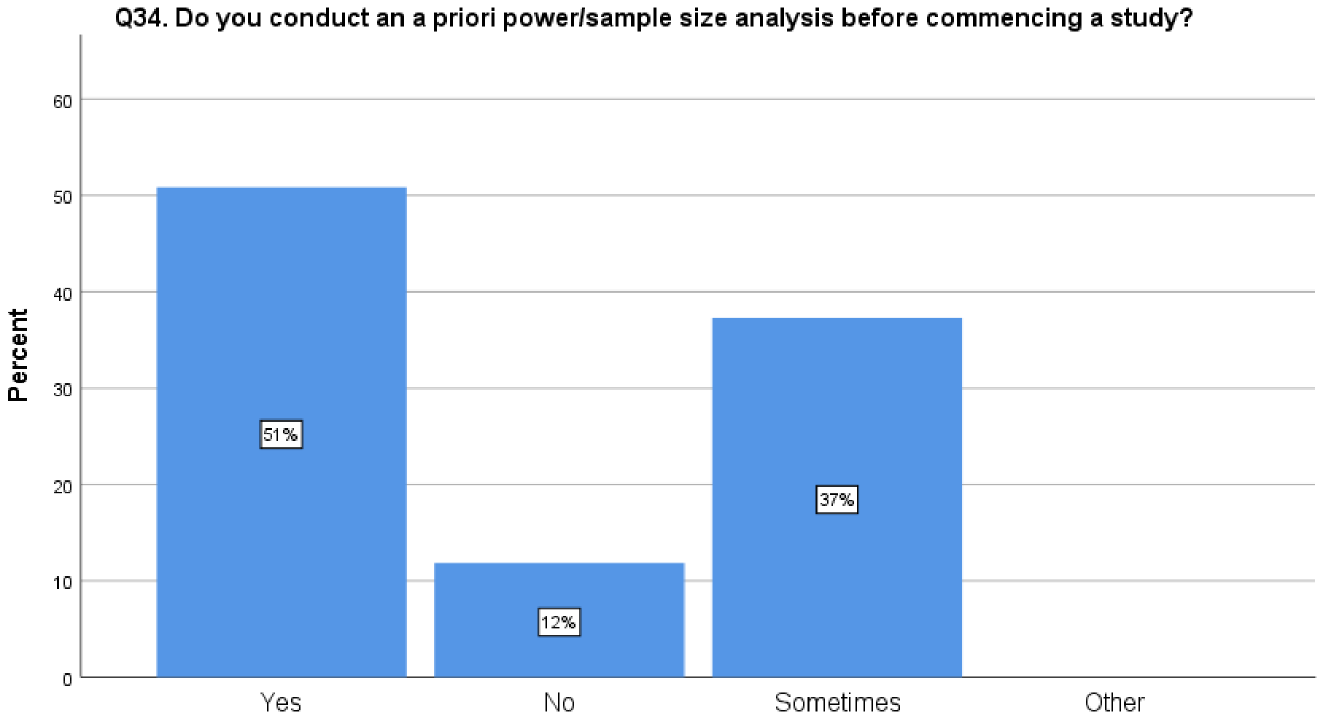
Q34. Do you conduct an a priori power/sample size analysis before commencing a study?
Percent
0
10
20
30
40
50
60
51%
12%
37%
Yes
No
Sometimes
Other

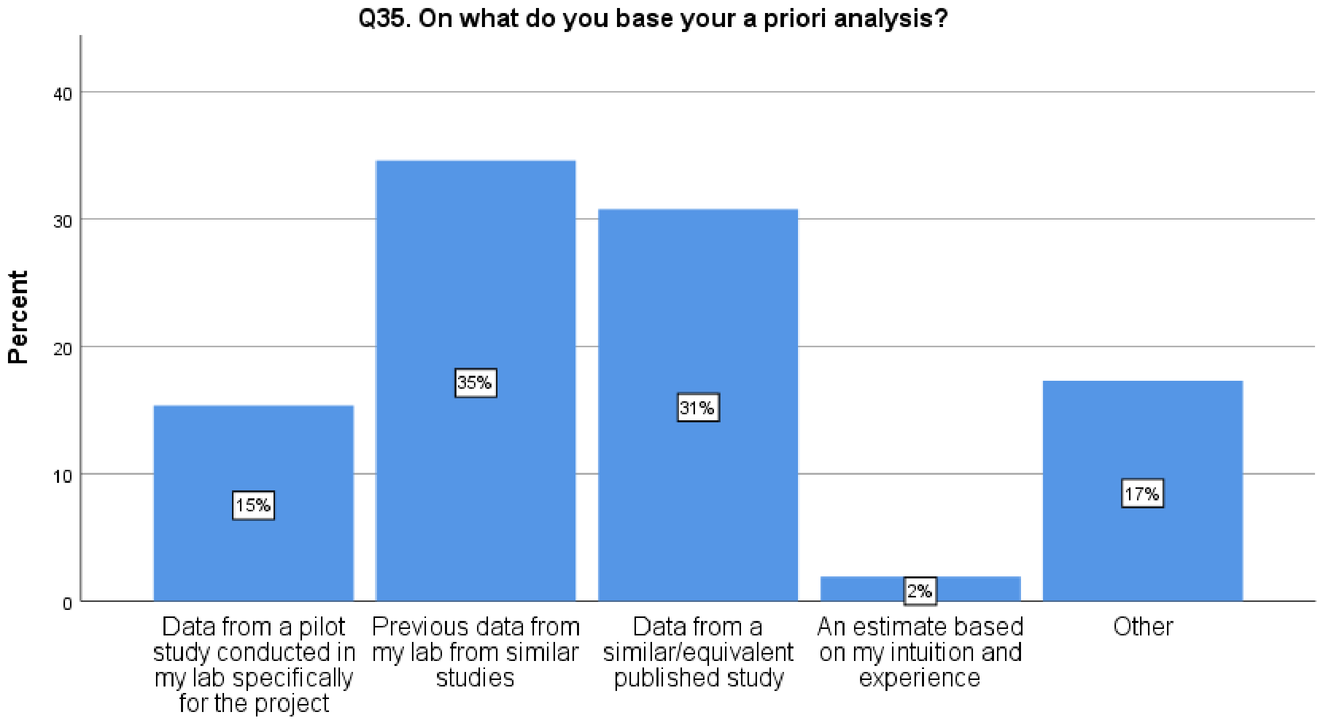
Q35. On what do you base your a priori analysis?
Percent
0
10
20
30
40
15%
35%
31%
2%
17%
Data from a pilot study conducted in my lab specifically for the project
Previous data from my lab from similar studies
Data from a similar/equivalent published study
An estimate based on my intuition and experience
Other

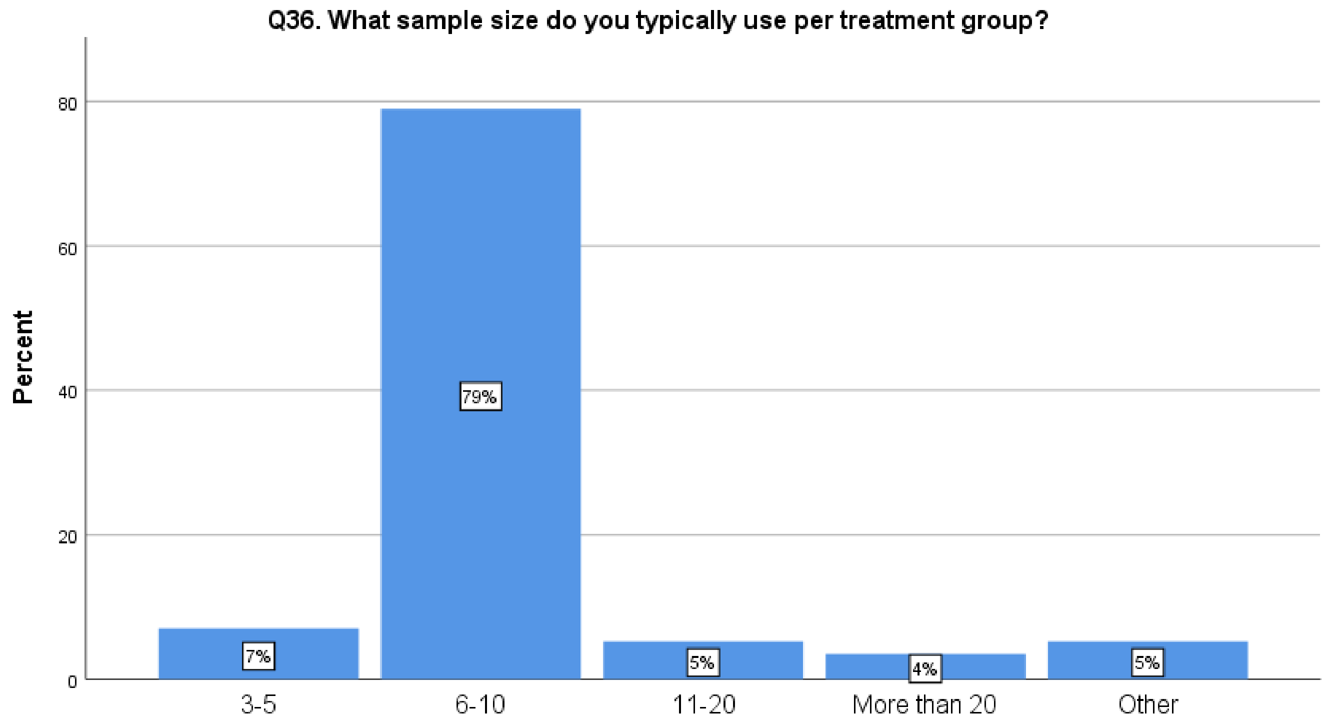
Q36. What sample size do you typically use per treatment group?
Percent
80
60
40
20
0
79%
7%
5%
4%
5%
3-5
6-10
11-20
More than 20
Other

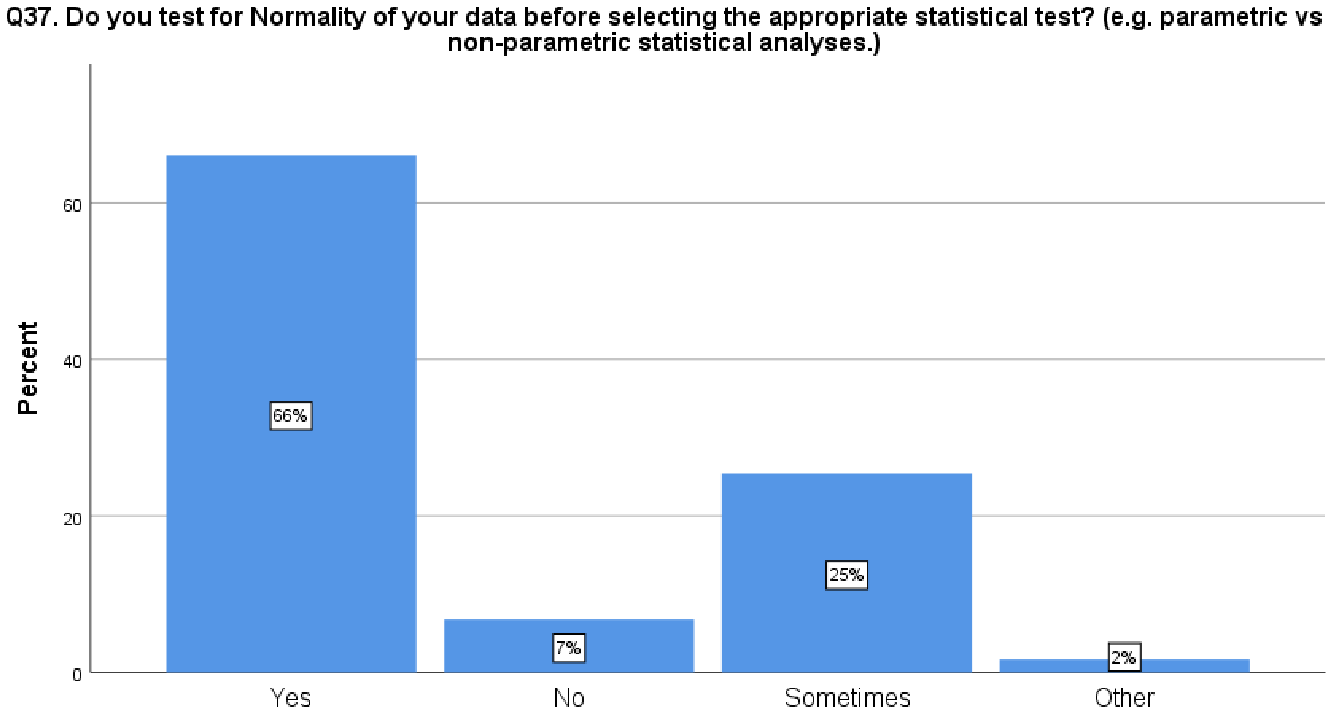
Q37. Do you test for Normality of your data before selecting the appropriate statistical test? (e.g. parametric vs non-parametric statistical analyses.)
Percent
60
40
20
0
66%
7%
25%
2%
Yes
No
Sometimes
Other

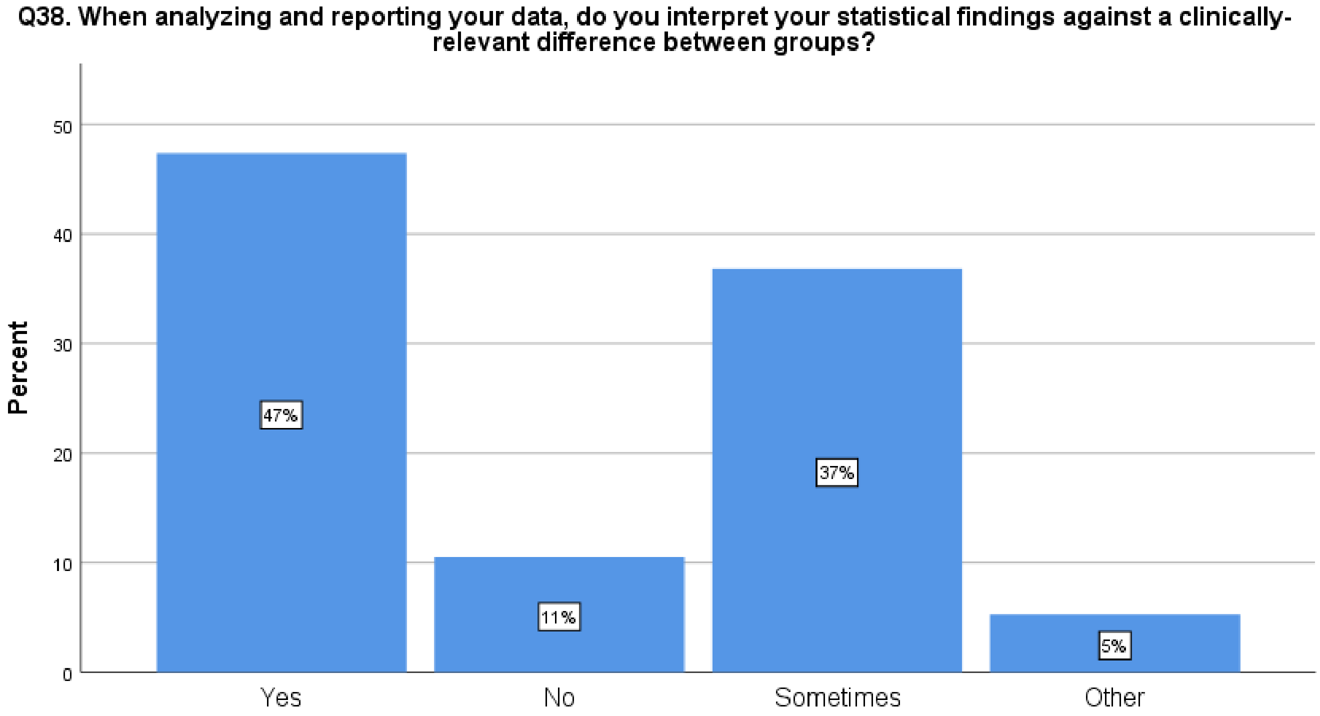
Q38. When analyzing and reporting your data, do you interpret your statistical findings against a clinically-relevant difference between groups?
Percent
50
40
30
20
10
0
47%
11%
37%
5%
Yes
No
Sometimes
Other

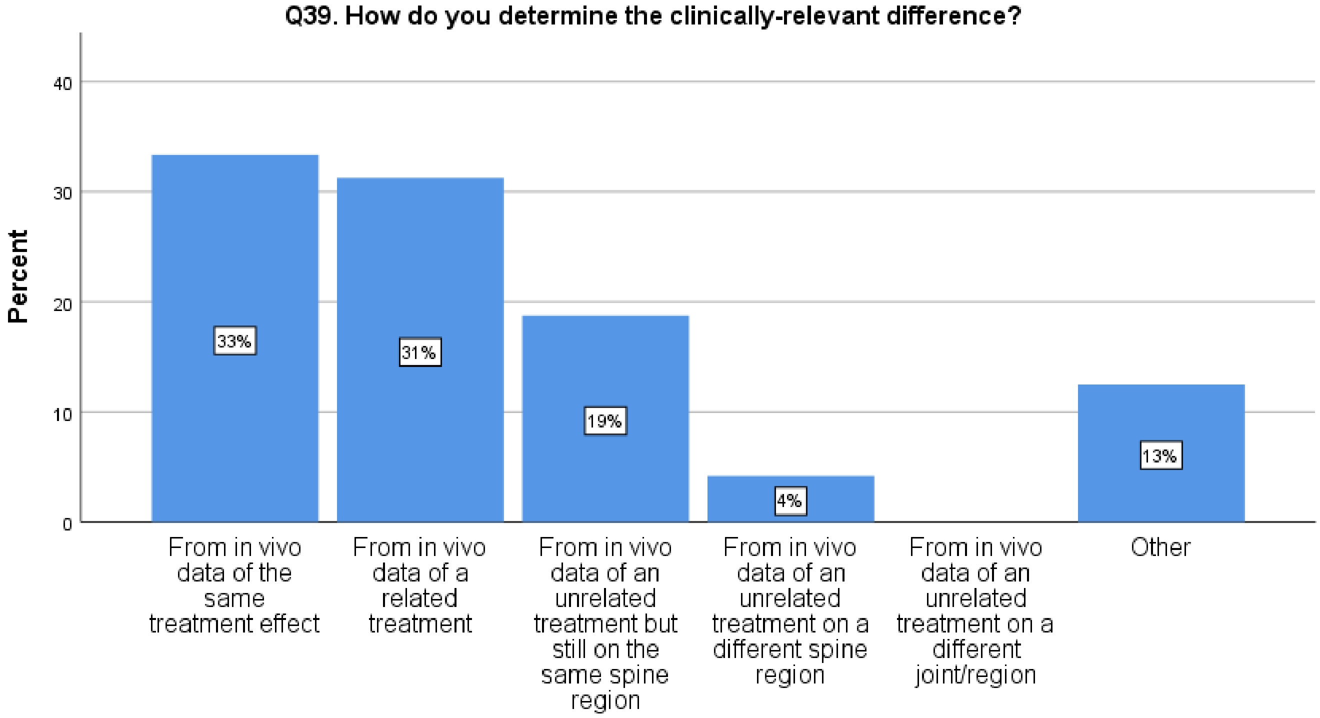
Q39. How do you determine the clinically-relevant difference?
Percent
40
30
20
10
0
33%
31%
19%
4%
13%
From in vivo data of the same treatment effect
From in vivo data of a related treatment
From in vivo data of an unrelated treatment but still on the same spine region
From in vivo data of an unrelated treatment on a different spine region
From in vivo data of an unrelated treatment on a different joint/region
Other

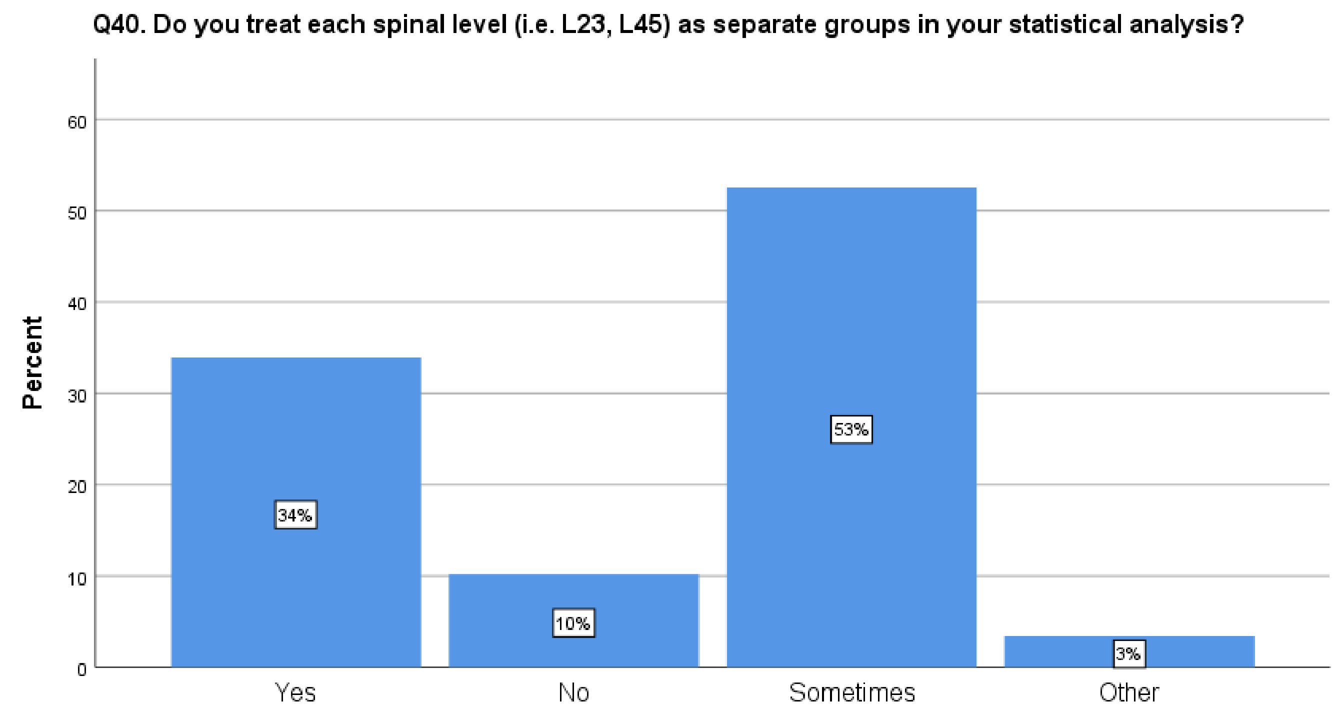
Q40. Do you treat each spinal level (i.e. L23, L45) as separate groups in your statistical analysis?
Percent
60
50
40
30
20
10
0
34%
10%
53%
3%
Yes
No
Sometimes
Other

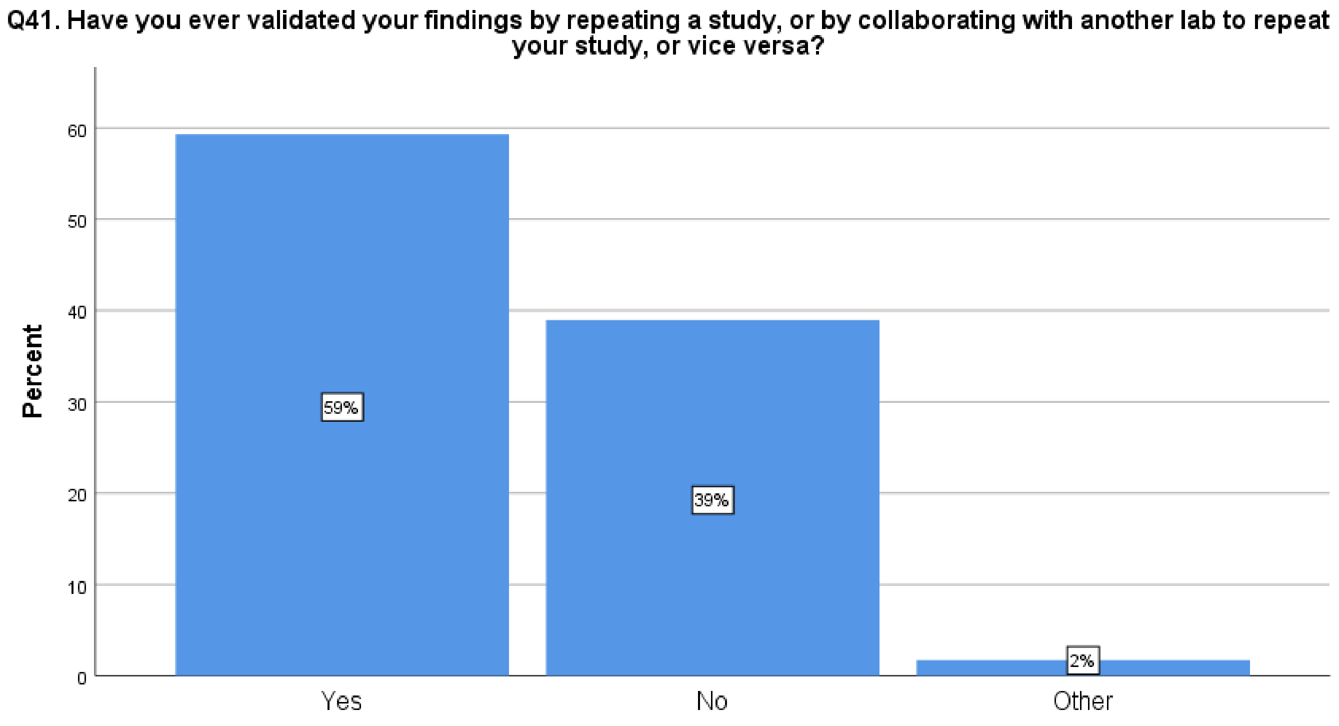
Q41. Have you ever validated your findings by repeating a study, or by collaborating with another lab to repeat your study, or vice versa?
Percent
60
50
40
30
20
10
0
59%
39%
2%
Yes
No
Other

Q42 [General Information] I have personally been involved in the following types of research (check all that apply): (84 responses)

1. Clinical trials.
2. Computer simulations including finite element analysis.
3. In vivo small animal (rodent) studies.
4. In vivo large animal (non-rodent) studies.
5. In vitro (cadaver tissue) studies.
6. In vitro (cell) studies.
7. Bench mechanical testing (non-tissue based, i.e. ASTM).
8. None.
9. Other.

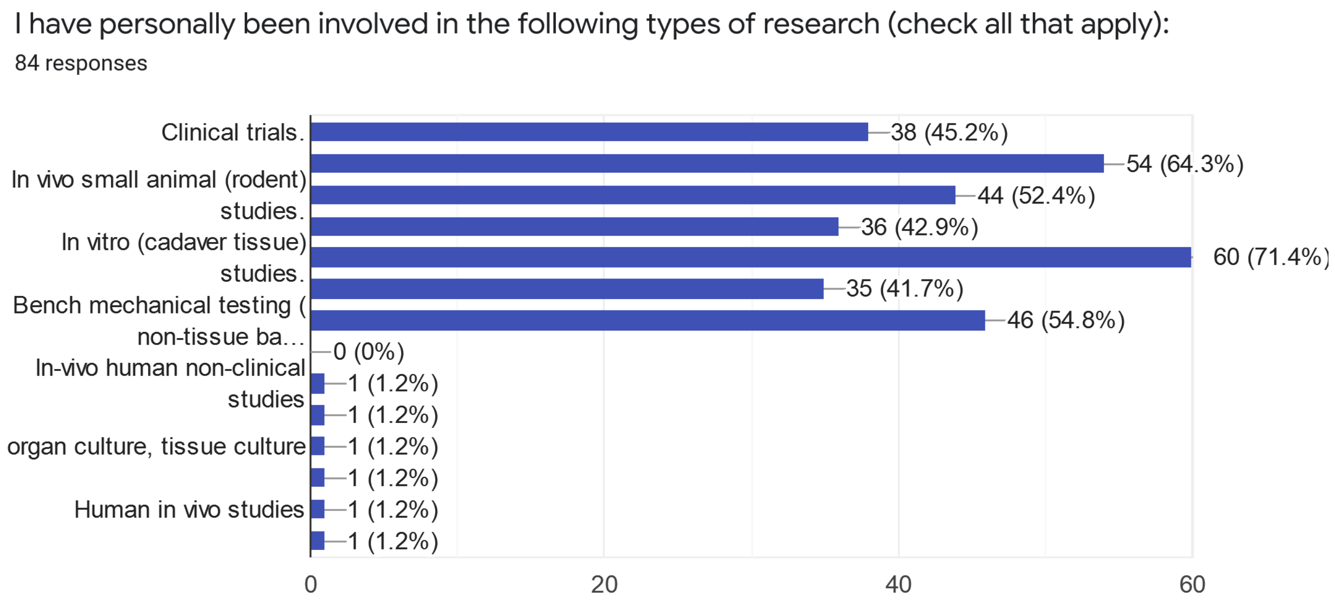

Thank you for taking the time to complete the survey. If you would like to have your name included in the consensus paper as participating in this survey, please include your name and affiliation. (64 responses)
