# Supplementary material for: Spine biomechanical testing methodologies: The controversy of consensus vs scientific evidence
Source: JOR Spine. 2021 Jan 5;4(1):e1138. doi: 10.1002/jsp2.1138 (PMC7984003; doi:10.1002/jsp2.1138)
Supplement: Supplementary file 2 — Appendix S2: List of survey responders [file JSP2-4-e1138-s001.pdf]

Supplemental Material S2: List of Survey Responders

Surname	First Name	Affiliation	Email
Anderson	Dennis E.	Harvard Medical School / Beth Israel Deaconess Medical Center	danders7@bidmc.harvard.edu
Andersson	Gunnar	Rush University Medical Center	Gunnar_andersson@rush.edu
Bowden	Anton	Brigham Young University	abowden@byu.edu
Buckley	Conor	Trinity College Dublin Ireland	conor.buckley@tcd.ie
Caprara	Sebastiano	ETH Zurich	sebastiano.caprara@hest.ethz.ch
Chiba	Kazuhiro	National Defense Medical College	kchiba5726@gmail.com
Cholewicki	Jacek	Michigan State University Center for Orthopedic Research	cholewic@msu.edu
Colbrunn	Robb	Cleveland Clinic	colbrur@ccf.org
Costi	John J.	Flinders University	john.costi@flinders.edu.au
Cristofolini	Luca	Alma Mater Studiorum - Università di Bologna	luca.cristofolini@unibo.it
Diwan	Ashish	University of New South Wales	A.diwan@unsw.edu.au
Dvorak	Jiri	Schulthess Clinic Zurich	jiri@dvorakmedical.com
Ellingson	Arin	University of Minnesota	ellin224@umn.edu
El-Rich	Marwan	Khalifa University	marwan.elrich@ku.ac.ae
Espinoza	Alejandro	Rush University Medical Center	Alejandro_Espinoza@rush.edu
Farahpour	Nader	Bu Ali Sina University	naderfarahpour1@gmail.com
Ferguson	Stephen	ERH Zurich	fstephen@ethz.ch
Fields	Aaron	University of California, San Francisco	aaron.fields@ucsf.edu
Gadomski	Ben	Colorado State University	ben.gadomski@colostate.edu
Gardner-Morse	Mack	University of Vermont	mack.gardner-morse@uvm.edu
Gill	Kevin	UT Southwestern Medical Center-Dallas	kevin.gill@utsouthwestern.edu
Goel	Vijay	University of Toledo	Vijay.Goel@utoledo.edu
Grad	Sibylle	AO Research Institute Davos	sibylle.grad@aofoundation.org
Gregory	Diane	Wilfrid Laurier University	dgregory@wlu.ca
Higashino	Kosaku	Shikoku Medical Center for Children and Adults	kosahigasino@gmail.com
Holguin	Nilsson	IUPUI	nholguin@iupui.edu
Holsgrove	TImothy	University of Exeter	t.holsgrove@exeter.ac.uk
Hoon	Jeon Chang	AJOU Univ. Medical Center	chjeon@ajou.ac.kr
Hukins	David		david_hukins@yahoo.co.uk
Iatridis	James C.	Icahn School of Medicine at Mount Sinai	james.iatridis@mssm.edu
Kawakami	Mamoru	Wakayama Medical University Kihoku Hospital	kawakami@wakayama-med.ac.jp
Kennedy	David J.	Vanderbilt University Medical Center	david.j.kennedy@vumc.org

Kim	Joohan	Korea University	nskjh94@korea.ac.kr
La Barbera	Luigi	Politecnico di Milano. 2: Polytechnique Montréal.	luigi.labarbera@polimi.it
Ledet	Eric	Rensselaer Polytechnic Institute	ledete@rpi.edu
Liebsch	Christian	Ulm University	christian.liebsch@uni-ulm.de
Macedo	Luciana	McMaster University	macedol@mcmaster.ca
Mannen	Erin	University of Arkansas for Medical Sciences	emannen@uams.edu
Martin	John	Duke University	jtm56@duke.edu
Mauck	Robert L.	University of Pennsylvania	lemauck@mail.med.upenn.edu
Newell	Nicolas	Imperial College London	n.newell09@imperial.ac.uk
O'Connell	Grace	University of California - Berkeley	g.oconnell@berkeley.edu
Palanca	Marco	University of Sheffield	m.palanca@sheffield.ac.uk
Punt	Michiel		michiel.punt@hu.nl
Puttlitz	Christian	Colorado State University	christian.puttlitz@colostate.edu
Ramos Pascual	Sonia	University of Bath	soniaramospascual@gmail.com
Raphaël	Vialle	Armand Trousseau Hospital	raphael.vialle@aphp.fr
Russo	Michael P.	Flinders University	Michael.russo@flinders.edu.au
Sakai	Toshinori	Tokushima university	norinoridowluck@yahoo.co.jp
Shirado	Osamu	AMEC at Fukushima Medical University	oshirado@gmail.com
Sloan	Stephen	Cornell University	srs387@cornell.edu
Smit	Theodoor H.	Amsterdam UMC	th.smit@amsterdamumc.nl
Stokes	Ian	University of Vermont	ian.stokes@uvm.edu
Stone	Laura S .	McGill University	laura.s.stone@mcgill.ca
Suzuki	Miyako		miyakosuzuki170@chiba-u.jp
Turner	Alex		aturner@atecspine.com
Vadalà	Gianluca	Campus Bio-Medico University of Rome	G.vadala@gmail.com
Walsh	William	UNSW Sydney	w.walsh@unsw.edu.au
Wilke	Hans-Joachim	Ulm University	hans-joachim.wilke@uni-ulm.de
Yamada	Kentaro	Fuchu Hospital	yamachen@msic.med.osaka-cu.ac.jp
Yoon	Tim	Emory	styoon@emory.edu
Yurube	Takashi	Kobe University Graduate School of Medicine	takayuru-0215@umin.ac.jp
Zander	Thomas	Charité - Universitätsmedizin Berlin, Julius Wolff Institut	thomas.zander@charite.de
